# 4-aroylpiperidines and 4-(α-hydroxyphenyl)piperidines as selective sigma-1 receptor ligands: synthesis, preliminary pharmacological evaluation and computational studies

**DOI:** 10.1186/s13065-016-0200-1

**Published:** 2016-08-23

**Authors:** Hermia N. Ikome, Fidele Ntie-Kang, Moses N. Ngemenya, Zhude Tu, Robert H. Mach, Simon M. N. Efange

**Affiliations:** 1 Department of Chemistry, Faculty of Science, University of Buea, P.O. Box 63, Buea, South West Region Cameroon; 2 Department of Pharmaceutical Chemistry, Martin-Luther University of Halle-Wittenberg, Wolfgang-Langenbeck-Str. 4, 06120 Halle (Saale), Germany; 3 Biotechnology Unit, Department of Biochemistry and Molecular Biology, Faculty of Science, University of Buea, P.O.Box 63, Buea, South West Region Cameroon; 4 Department of Radiology, University of Washington School of Medicine, Seattle, USA

**Keywords:** Sigma-1 binding, Piperidines, QSAR, Pharmacophore

## Abstract

**Background:**

Sigma (σ) receptors are membrane-bound proteins characterised by an unusual promiscuous ability to bind a wide variety of drugs and their high affinity for typical neuroleptic drugs, such as haloperidol, and their potential as alternative targets for antipsychotic agents. Sigma receptors display diverse biological activities and represent potential fruitful targets for therapeutic development in combating many human diseases. Therefore, they present an interesting avenue for further exploration. It was our goal to evaluate the potential of ring opened spipethiane (**1**) analogues as functional ligands (agonists) for σ receptors by chemical modification.

**Results:**

Chemical modification of the core structure of the lead compound, (**1**), by replacement of the sulphur atom with a carbonyl group, hydroxyl group and 3-bromobenzylamine with the simultaneous presence of 4-fluorobenzoyl replacing the spirofusion afforded novel potent sigma-1 receptor ligands **7a–f**, **8a–f** and **9d–e**. The sigma-1 receptor affinities of **7e**, **8a** and **8f** were slightly lower than that of **1** and their selectivities for this receptor two to threefold greater than that of **1**.

**Conclusions:**

It was found that these compounds have higher selectivities for sigma-1 receptors compared to **1**. Quantitatitive structure–activity relationship studies revealed that sigma-1 binding is driven by hydrophobic interactions.

**Graphical abstract:**
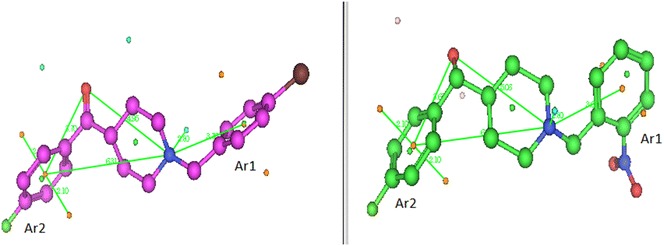
Identified pharmacophore features for sigma binding.

**Electronic supplementary material:**

The online version of this article (doi:10.1186/s13065-016-0200-1) contains supplementary material, which is available to authorized users.

## Background

Sigma (σ) receptors are membrane-bound proteins that bind several psychotropic drugs with high affinity [1]. They were initially proposed to be related to opioid receptors [2] but were later found to be a distinct pharmacological entity distinguished by an unusual promiscuous ability to bind a wide variety of drugs [3]. Initial interest in σ receptors came mainly from their high affinity for typical neuroleptic drugs, such as haloperidol, and their potential as alternative targets for antipsychotic agents [4, 5]. However, no endogenous functional ligand (agonist) for σ receptors has been conclusively identified.

These receptors are classified into two subtypes: subtype 1 (σ_1_ receptor) and subtype 2 (σ_2_ receptor) which are differentiated by their pharmacological profiles, distribution in tissues, functions, and molecular sizes [6], with the σ_1_ being the most documented. Basically, the σ_1_ receptor is believed to have a ligand binding profile such that (+)-benzomorphans are at least fivefold to tenfold more potent than their corresponding (−)-isomers [7]. On the other hand, for the σ_2_ subtype, the (−)-benzomorphans are more potent than their corresponding (+)-isomers in the binding assay. The gene coding the σ_1_ receptor has been isolated and cloned from guinea pig [8], mouse [9], rat [10], and human [11]. The protein coded by the σ_1_ receptor gene in rat brain consists of a 223 amino acid sequence (23 kDa). In contrast, the σ_2_ receptor has not been cloned yet and is estimated to have a molecular weight of 19–21.5 kDa [12]. The presence of a σ_3_ subtype has not been confirmed yet, even though its existence was proposed in a few papers [13–15].

The specific participation and character of σ receptors in the processes of the psychiatric and neurological disorders is still not clear [16]. Nevertheless, some of the ligands have drawn attention as potentially useful antipsychotics, antidepressants [17, 18], anxiolytics [19], anti-amnesics, for mental improvement [20], analgesics [21], anti-epileptics, anticonvulsants, and as seizure reducing neuroprotective agents [22]. Apart from their involvement in psychiatric disorders and nervous system diseases, σ receptors and their ligands offer a plethora of means for dealing with several cancer cell types through a variety of strategies [23]. A typical endogenous σ_1_ receptor regulator is the hallucinogen *N*,*N*-dimethyltryptamine [24]. Moreover, σ_1_ receptor ligands have recently been shown to be potent noncompetitive antagonists at the *N*-methyl-d-aspartate (NMDA) receptor with IC_50_ values similar to those of the dissociative anesthetic (S)-(+)-ketamine [25]. Ghandi et al. recently carried out a one pot synthesis of new spirocyclic-2,6-diketopiperazine derivatives, with benzylpiperidine and cycloalkane moieties, some of which showed up to a 95-fold σ_1_/σ_2_ selectivity ratio [26].

**Fig. 1:**
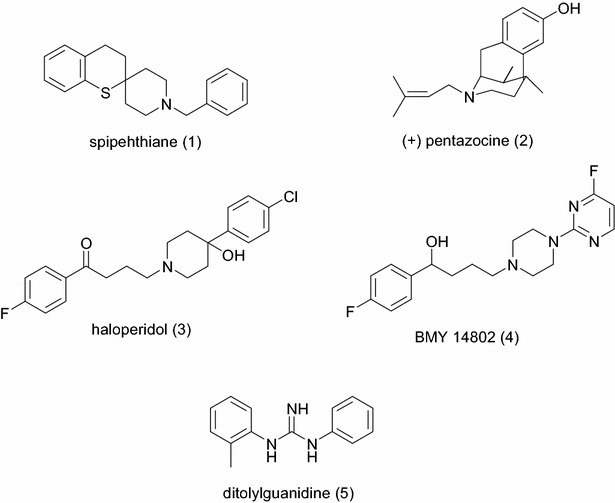
Some sigma receptor ligands

**Fig. 2:**
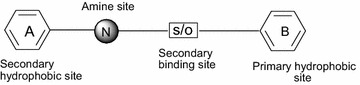
Gund’s pharmacophore model

**Fig. 3:**
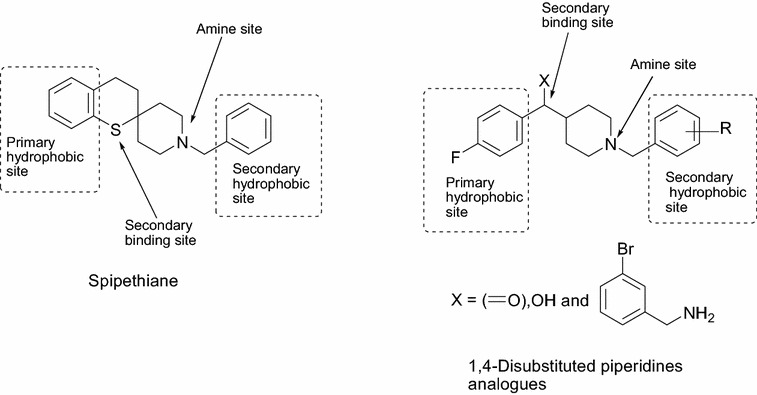
Design of 1,4-disubstituted piperidines from proposed pharmacophore model for sigma receptor ligands

Over the years, a large number of compounds with unrelated chemical structures have been reported to display affinity for σ receptors (Fig. 1). To explain the binding of these structurally diverse compounds to sigma receptors, a number of models or pharmacophores have been proposed [13, 27–33]. Generally, the pharmacophore (ph4) for σ_1_ receptor binding consists of three major sites: an amine site as an essential proton acceptor site, flanked by two hydrophobic domains, a primary hydrophobic site that binds phenyl group “B” from the central amine and a secondary binding site that binds phenyl group “A” from the central amine (Fig. 2). Gund and coworkers [13] suggested that the chains between the amine site and aromatic rings need not be simple alkyl chains. They could bear a polar substituent such as S or O, which could be considered as the second binding site (Fig. 3). Other functional groups can be piperidyl, guanidinyl, pyrrolidyl, piperazyl, thiochromanyl, and benzamidyl [7]. A pharmacophore for σ_2_ binding has also been proposed [24, 30–33]. The latter is also characterized by a central amine site flanked by two hydrophobic sites. However, the two models (σ_1_ and σ_2_) differ in a number of respects, such as the distance between the central amine site and the hydrophobic sites [13, 32].

Owing to the apparent involvement of σ receptors in a variety of biological processes, and the potential applications of σ ligands in pharmacology and medicine, interest in these receptors and their ligands has remained high, and there is a continuing search for new selective ligands that can serve either as agonists or antagonists at those biological processes that are mediated by σ receptors.

Among the compounds reported to bind σ receptors, a large number of benzylpiperidine and benzylpiperazine derivatives display remarkable affinity. In reviewing these collections of compounds, our attention was attracted to spipethiane (**1**), a spirocyclic compound that contains the elements of benzylpiperidine. Spipethiane is a very potent and selective ligand for σ_1_ receptors (*K*_*i*_: σ_1_, 0.5 nM; σ_2_, 416 nM) [34]. The design of this compound was inspired by the reported high affinity of the spirotetralins (**2**) for σ_1_ receptors. In contrast to the spirotetralins, spipethiane does not display high affinity for 5-HT_2_ receptors. Consequently, the compound was one of the most selective σ_1_ ligands reported at the time. Since this initial discovery, the work on this spirocyclic skeleton has been extended to include several compounds which are reported to display high affinity and selectivity for σ_1_ receptors, and potential antitumor activity [33, 35]. Spipethiane and it analogues therefore provide interesting targets for further investigation.

Deprived of conformational freedom, spirocyclic compounds such as spipethiane and the spirotetralins may derive their receptor selectivity from their ability to adopt only a restricted number of molecular conformations. The current study sought to investigate the role of spirofusion in the biological activity of the spipethiane/spirotetralin skeleton. The compounds obtained from the study were tested for binding to σ_1_ and σ_2_ receptors.

## Results and discussion

**Scheme 1:**
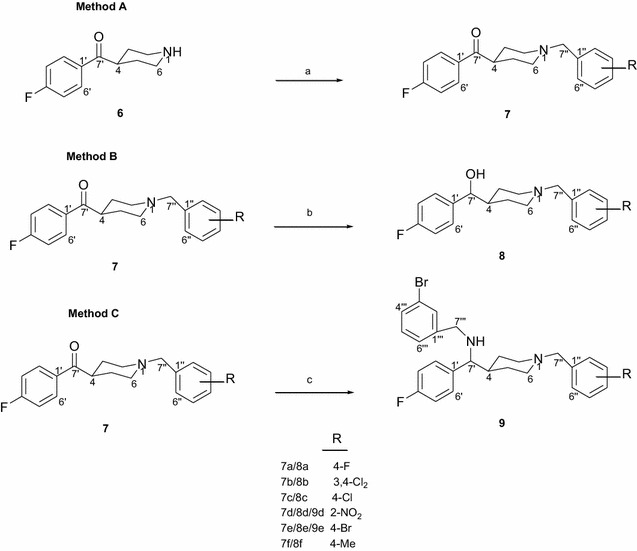
Reagents: **a** Substituted benzyl halide, EtOH, H_2_O, NaOAc reflux; **b** LiBH_4_, THF, reflux; **c** 3-bromobenzylamine hydrochloride, LiBH_4_, THF, HOAc, reflux

Compounds **7a–f**, **8a–f** and **9d–e** were synthesized according to methods A–C reported in Scheme 1. Reaction of 4-(4-fluorobenzoyl)piperidine with various substituted benzyl halides in the presence of sodium acetate, in aqueous ethanol afforded the methanone analogues **7a–f** [36] (Scheme 1, method A). Reduction of the methanone analogues with LiBH_4_ in THF provided the corresponding alcohols **8a–f** [33] (Scheme 1, method B). Reductive amination of the methanone analogues **7d** and **7e** with 3-bromobenzylamine afforded **9d** and **9e** (Scheme 1, method C) [37]. The synthesized 1,4-disubstituted piperidine derivatives were evaluated for their affinity at both σ_1_ and σ_2_ receptors.

### Sigma receptor binding

Overall, the majority of target compounds displayed significantly higher affinity for σ_1_ receptors than for σ_2_ receptors (Tables 1, 2, 3). *K*_*i*_ values at σ_1_ receptors were below 15 nM for all the compounds except **7b**, **7d**, **8b** and **8d**. In contrast, all compounds except **7a** and **7f** were found to have *K*_*i*_ values greater than 500 nM at the σ_2_ receptor. Among the ketones, **7e** and **7a** emerged as the most potent σ_1_ receptor ligands followed closely by **7c** and **7f**. All four compounds are *para* substituted, suggesting that this substitution pattern is favored. In contrast, substitution at the *ortho* or disubstituted at *meta* and *para* positions was disfavoured, as both the 2″-nitro compound (**7d**) and 3″, 4″-dichloro substituted analogue (**7b**) displayed equally poor affinity for the σ_1_ receptor. Reduction of the carbonyl compounds to the corresponding alcohols led to a significant increase in σ_1_ receptor affinity for the most potent ligands: 11-fold for **7f** versus **8f**; twofold for both **7a** versus **8a** and **7c** versus **8c**. In contrast, for compounds **7e** versus **8e** the σ_1_ binding affinity decreased fivefold upon reduction of the carbonyl to the corresponding alcohol. Compounds **7e**, **8a** and **8f** exhibited the highest selectivity (ki-σ_2_/σ_1_ = 610, 606 and 589 respectively) for σ_1_ receptors among all compounds tested, with *K*_*i*_ values between 1.00 to 2.00 nM and >500 nM for σ_2_ receptors; their affinities were slightly lower than that of spipethiane (*K*_*i*_: σ_1_, 0.50 nM; σ_2_, 416 nM; ki-σ_2_/σ_1_ = 208) [34] but greater than that of (+)-pentazocine (*K*_*i*_: σ_1_, 3.58 ± 0.20 nM; σ_2_, 1923 nM; ki-σ_2_/σ_1_ = 540) [38]. Therefore, these compounds are more selective than spipethiane. Compounds **7b**, **7d**, **8b**, **8d** and **9d** interact non-selectively with both receptor subtypes but with mediocre binding affinities (*K*_*i*_: σ_2_/σ_1_ = 2). In particular, *ortho* substitution with the nitro group results in mediocre binding affinity for both receptor subtypes (σ_1_: **7d** vs **8d** vs **9d**; σ_2_: **7d**, **8d** and **9d**), 2″-N). As a result, the nitro substituted analogues were found to be the least σ_1_/σ_2_ receptor selective ligands (*K*_*i*_: σ_2_/σ_1_ = 2). We conclude that replacement of the spirofusion in spipethiane with a hydroxymethylene or carbonyl group preserves affinity and selectivity for σ_1_ receptors.

**Table 1:** Binding affinity of methanone analogues

Compound	R	σ_1_ (*K*_*i*_ nM)	σ_2_ (*K*_*i*_ nM)	Selectivity ratio (*K*_*i*_ σ_2_/σ_1_)
**7a**	4″-F	2.96 ± 0.5	221.64 ± 8.0	75
**7b**	3″,4″-Cl_2_	>434	>854	2
**7c**	4″-Cl	5.98 ± 0.41	554.03 ± 34.22	93
**7d**	2″-NO_2_	>434	>854	2
**7e**	4″-Br	1.40 ± 0.5	>854	610
**7f**	4″-Me	11.58 ± 0.26	151.47 ± 7.79	13
Spipethiane^a^		0.50	416	208
(+)-pentazozcine^b^		3.58	1932	540

^a^Data available from Ref. [24]

^b^Data available from Ref. [24]

**Table 2:** Binding affinity of methanol analogues

Compound	R	σ_1_ (*K*_*i*_ nM)	σ_2_ (*K*_*i*_ nM)	Selectivity ratio (*K*_*i*_ σ_2_/σ_1_)
**8a**	4″-F	1.41 ± 0.22	>854	606
**8b**	3″,4″-Cl_2_	>434	>854	2
**8c**	4″-Cl	2.49 ± 0.24	>854	343
**8d**	2″-NO_2_	526.53 ± 69	>854	2
**8e**	4″-Br	5.22 ± 0.3	>854	164
**8f**	4″-Me	1.45 ± 0.4	>854	589

**Table 3:** Binding affinity of bromobenzylamine analogues

Compound	R	σ_1_ (*K*_*i*_ nM)	σ_2_ (*K*_*i*_ nM)	Selectivity ratio (*K*_*i*_ σ_2_/σ_1_)
**9d**	2″-NO_2_	>434	>854	2
**9e**	4″-Br	2.95 ± 0.57	>854	289

### SAR and QSAR study

Gund et al. have reported the molecular modeling of several σ_1_ receptor specific ligands: PD144418, spipethiane, haloperidol and pentazocine in a bid to develop a ph_4_ for σ_1_ receptor-ligand binding under the assumption that all the compounds interact at the same receptor site [13]. The primary ph_4_ for the σ binding sites was defined by mapping the topographic arrangements of the phenyl ring, the N-atom, and N lone pair vector; a point was placed 2.8 Å tetrahedrally from N atoms to represent an interaction between a protonated N atom and its binding site; *dummy* atoms were built 3.5 Å above and below a phenyl ring to represent hydrophobic binding to a receptor. The distance from the C-center to the N atom was 7.14 Å, while that from O and C-center was 3.68 Å and from O to N atom was 4.17 Å. The choice of ligands used in the study was based on their potency, selectivity and structural diversity with their affinity ranging from 0.08 to 5.8 nM.

### Correlation of binding affinity to σ_1_ receptor and van der Waals surface areas, dipole moments and water accessible surface areas of target compounds

Table 4 shows the computed values for 3D van der Waals surface areas (*S*_*vdW*_), the 2D van der Waals surface areas (*A*_*vdW*_), the AM1 dipole moments (*μ*_*D*(*AM*1)_), the densities (*d*) and 3D water accessible surface areas (*S*_*wat*_), as well as the experimentally derived binding affinities (Δ*G*^exp^) and the predicted binding affinities (Δ*G*^*pred*^) obtained from the most significant derived QSAR equation. The three most significant QSAR Eqs. (1) to (3), were derived for 14 molecules (*N* = 14) and three molecular descriptors (*k* = 3):

**Table 4:** Computed molecular descriptors, experimental and theoretically obtained binding affinities for σ_1_ receptor (obtained with the best model, Eq. 3)

Compd	*d*	*S*_*vdW*_	*S*_*wat*_	*A*_*vdW*_	*μ*_*D*(*AM*1)_	Δ*G* ^exp^	Δ*G* ^*pred*^	Δ*G* ^*res*^
**7a**	1.03	328.65	550.09	304.36	1.55	−0.47	−1.02	0.55
**7b**	1.01	356.64	591.24	335.12	2.16	−2.64	−1.95	−0.69
**7c**	1.05	343.45	565.21	317.53	1.69	−0.78	−1.16	0.38
**7d**	1.07	347.51	564.98	328.09	6.67	−2.64	−2.78	0.14
**7e**	1.14	354.30	588.50	329.31	1.58	−0.15	−1.19	1.04
**7f**	0.97	345.98	575.78	317.19	3.13	−1.06	−0.33	−0.73
**8a**	1.03	341.05	554.61	309.59	1.64	−0.15	−0.30	0.15
**8b**	1.09	365.46	594.35	340.35	2.07	−2.64	−1.75	−0.89
**8c**	1.03	353.34	575.63	322.77	1.39	−0.39	−0.53	0.14
**8d**	1.05	356.39	568.95	333.33	4.69	−2.72	−2.54	−0.18
**8e**	1.12	363.84	588.79	334.55	1.55	−0.72	−1.02	0.3
**8f**	0.97	356.88	581.58	322.42	0.36	−0.16	0.24	0.4
**9d**	1.11	491.05	756.77	345.12	4.60	−2.64	−2.79	0.15
**9e**	1.18	502.38	783.44	459.34	1.64	−0.47	−0.49	0.02

These are the structures of the compounds with the assigned positions. Preferable to be inserted in the scheme

1 $$\Delta G^{\exp } = 0.11 + 0.19S_{vdW} - 0.21A_{vdW} - 0.001d;R^{2} = 0.71,RMSE = 0.58,F = 7.9$$

2 $$\Delta G^{\exp } = 0.22\,+\,0.14S_{vdW}\,-\,0.16A_{vdW}\,-\,0.018\mu_{D(AM1)} ; R^{2} = 0.74, RMSE = 0.54, F = 9.4$$

3 $$\Delta G^{\exp } = - 4.19\,+\,0.13S_{vdW}\,-\,0.20A_{vdW}\,+\,0.04S_{wat} ;R^{2} = 0.77, RMSE = 0.51, F = 11.2$$

where *RMSE* is the root mean square error and *F* is the Fischer statistic level of significance. It was observed that there was more than 50 % correlation with the different descriptors combined. This implies that there is a relationship between the σ_1_ receptor binding affinity of the target compounds and the selected parameters for the study. Multilinear regression analysis showed that the three dimensional hydrophobic (*S*_*vdW*_) and solvent accessible surface (*S*_*wat*_) parameters are important factors in the binding affinity of the 1,4-disubstituted piperidine analogues towards the σ_1_ receptor, because they have positive coefficients compared to densities and dipole moments. The influence of hydrophobic constants confirms the presence of a hydrophobic binding site at the σ_1_ receptor. The respective *R*^2^ values of 0.71, 0.74 and 0.77 indicate that we can account for about 70–80 % of the variability in binding affinity and the remaining 20–30 % of the variability in affinity cannot be accounted for by the use of the two to four parameters.

**Fig. 4:**
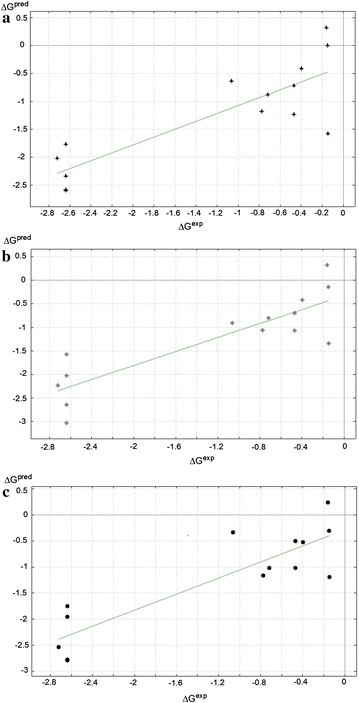
Correlation plot for three-descriptor QSAR relations **a** relation 1, **b** relation 2 and **c** relation 3

The correlation plots for QSAR Eqs. (1), (2) and (3) have been respectively shown in Fig. 4a–c. Interestingly, these plots showed similarity wherein points are grouped into two clusters. The clusters are formed such that the least potent σ_1_ ligands (characterized by substitution at the *ortho* and *meta* positions) are at the bottom left while the most potent ligands (characterized by substitution at the *para* position) are at the top right. Thus, the QSAR equations are able to discriminate between the active and inactive σ_1_ binders.

### Molecular electrostatic potential maps

**Fig. 5:**
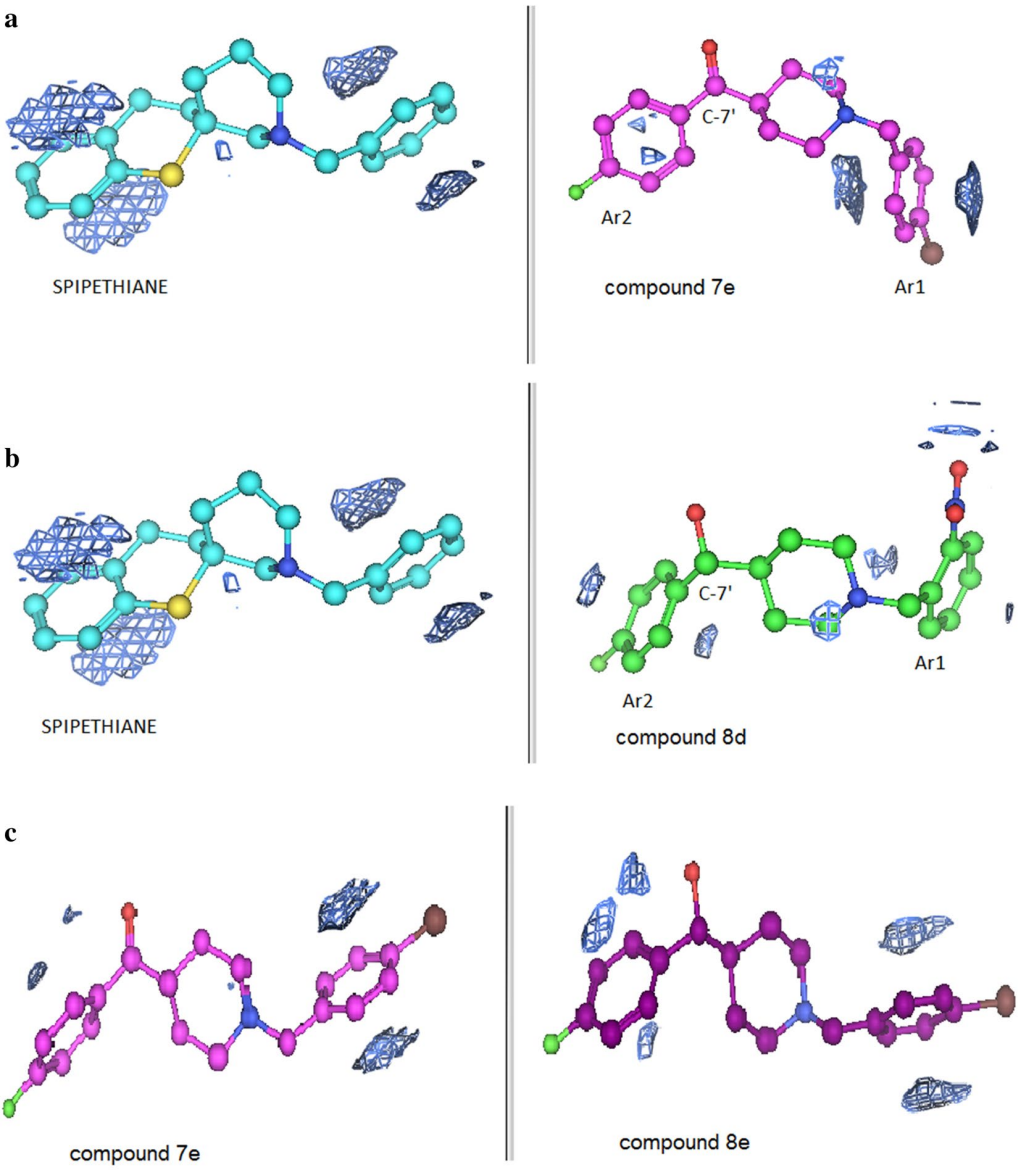
MEP maps for **a** spipethiane and the most potent σ_1_ ligand **7e**, **b** spipethiane and the least potent σ_1_ ligand **8d** and **c** the most potent **7e** and its corresponding alcohol, **8e**

Further structure–activity evaluation was performed by studying the electronic distribution of analogues through the use of molecular electrostatic potentials (MEPs). Electrostatic potential is the energy of interaction of a positive charge with the nuclei and electrons of a molecule. The MEP surfaces are color coded, with light brown indicating the hydrophobic regions, red the acceptor regions and blue, the donor regions (availability of lone pairs of electrons). The MEPs will be subsequently discussed for spipethiane (cyan carbons), the most potent ligand (purple carbons) (**7e**) and the least potent ligand (green carbons) (**8d**) for the σ_1_ binding affinity. These are illustrated in Fig. 5.

The main difference between the MEPs of spipethiane, the most potent and least potent ligands is observed around the secondary hydrophobic site (Ar1 region) where there is an additional field generated around the substituent of the pendant phenyl ring of the least potent ligand. This could be probably due to the fact that the nitro-group on the pendant phenyl group of the least potent ligand is strongly electron withdrawing and *ortho* substituted thereby pulling the electrons from the pendant phenyl group onto itself and as a result deactivating the ring.

**Fig. 6:**
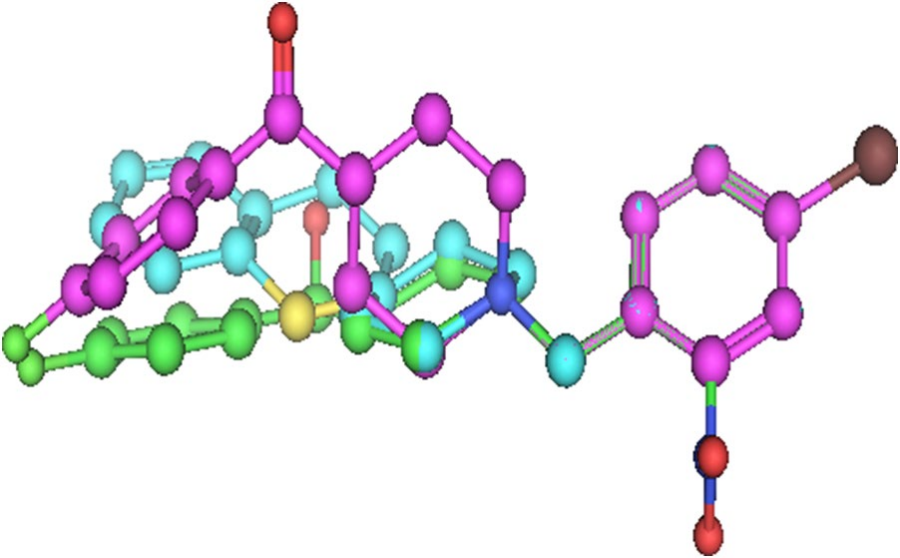
Superposition of spipethiane (*cyan*), the most potent (*purple*) and least potent (*green*) σ_1_ receptor ligands

MEP maps generated for the most potent ligand (**7e**) and its corresponding alcohol (**8e**) show no significant difference (Fig. 5c), in agreement with the observation that both ligands are potent σ_1_ receptor binders. Therefore, the difference between the most potent and least potent ligands lies in the type of substituent and position of substitution on the pendant phenyl group. The superposition of spipethiane (cyan), the most potent (purple) and least potent (green) σ_1_ receptor ligands is shown in Fig. 6. A difference observed when spipethiane (cyan) and the most potent σ_1_ ligand (purple) are superimposed is at the Ar2 portion (the primary hydrophobic site). Although the superposition around this site is not perfect, both ligands remain potent binders to the σ_1_ receptor, with high affinity and selectivity. This is possible because phamacophore studies for σ_1_ receptor binding have shown that this site is associated with much bulk tolerance [13].

### Molecular surfaces of ligands

**Fig. 7:**
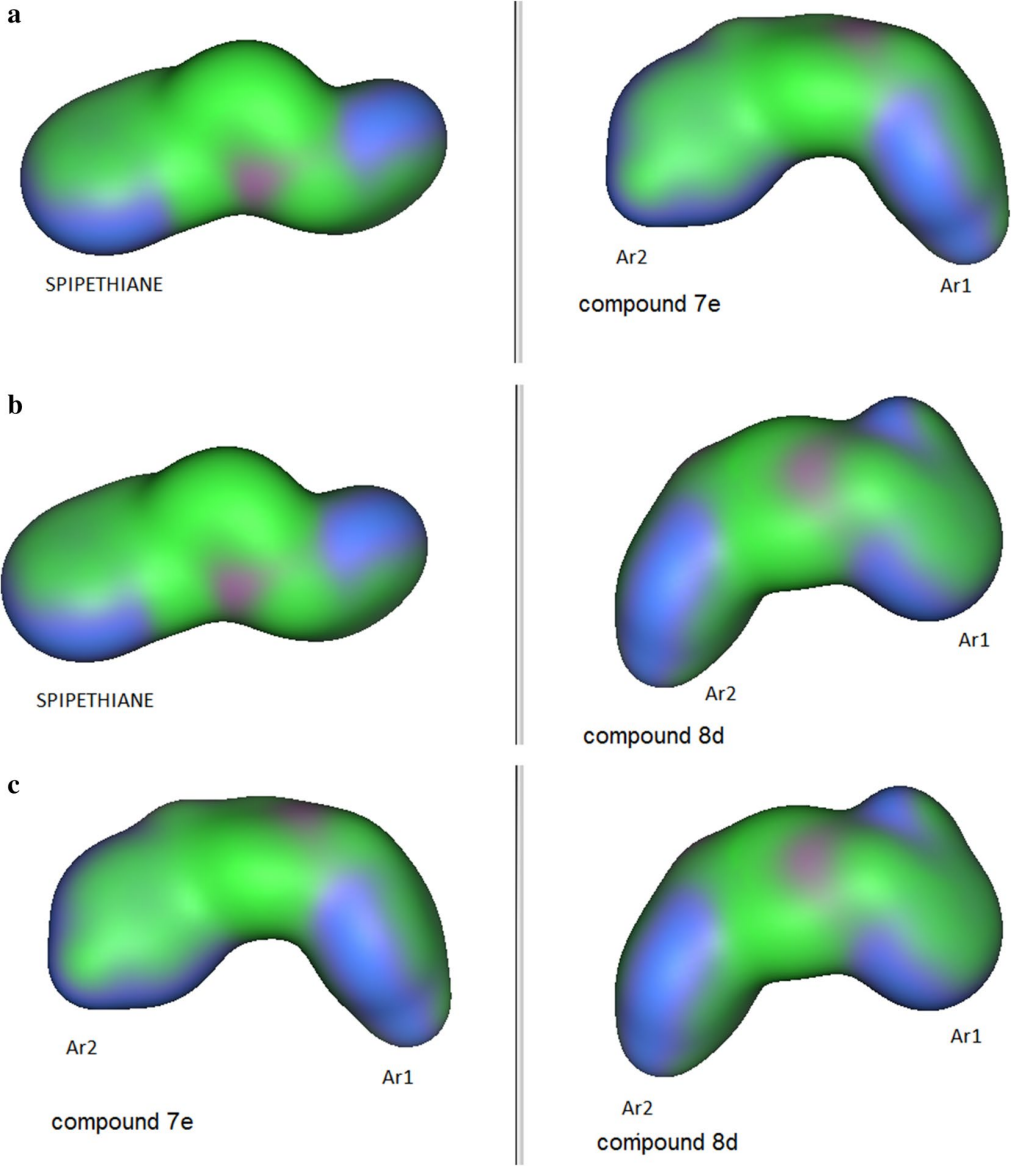
Molecular surfaces map for **a** spipethiane and most potent σ_1_ ligand, **b** spipethiane and least potent σ_1_ ligand and **c** most potent and least potent σ_1_ ligands

The molecular surfaces of 1,4-disubstituted piperidine analogues were studied to further evaluate the structure–activity relationships. Molecular surface maps provide an efficient way of comparing molecular shape and property. They are color coded, with blue indicating the mildly polar regions, green indicating the hydrophobic regions and purple indicating the H-bonding regions. Discussion on the MEPs will be for spipethiane, the most potent (**7e**) and the least potent σ_1_ ligand (**8d**), illustrated in Fig. 7.

The molecular shape of the geometry optimized spipethiane structure is different from those of the most potent and least potent ligands in that, the former is linear while the latter are curved. However, the direction of the curvature is not identical for geometry optimized structures of the two compounds. Interestingly, there is some consistency in the hydrophobic regions of spipethiane and the most potent ligand (Fig. 7a), compared to the least potent ligand and spipethiane (Fig. 7b). The molecular surface of the least potent ligand is characterized mostly by the mild polar and H-bond regions instead of the hydrophobic regions as seen for spipethiane and the most potent ligand. Therefore, we can conclude that molecular shape has minimal influence on affinity for this series of compounds since the most potent ligand is different from spipethiane in shape but similar to the least potent ligand.

### Pharmacophore study

**Fig. 8:**
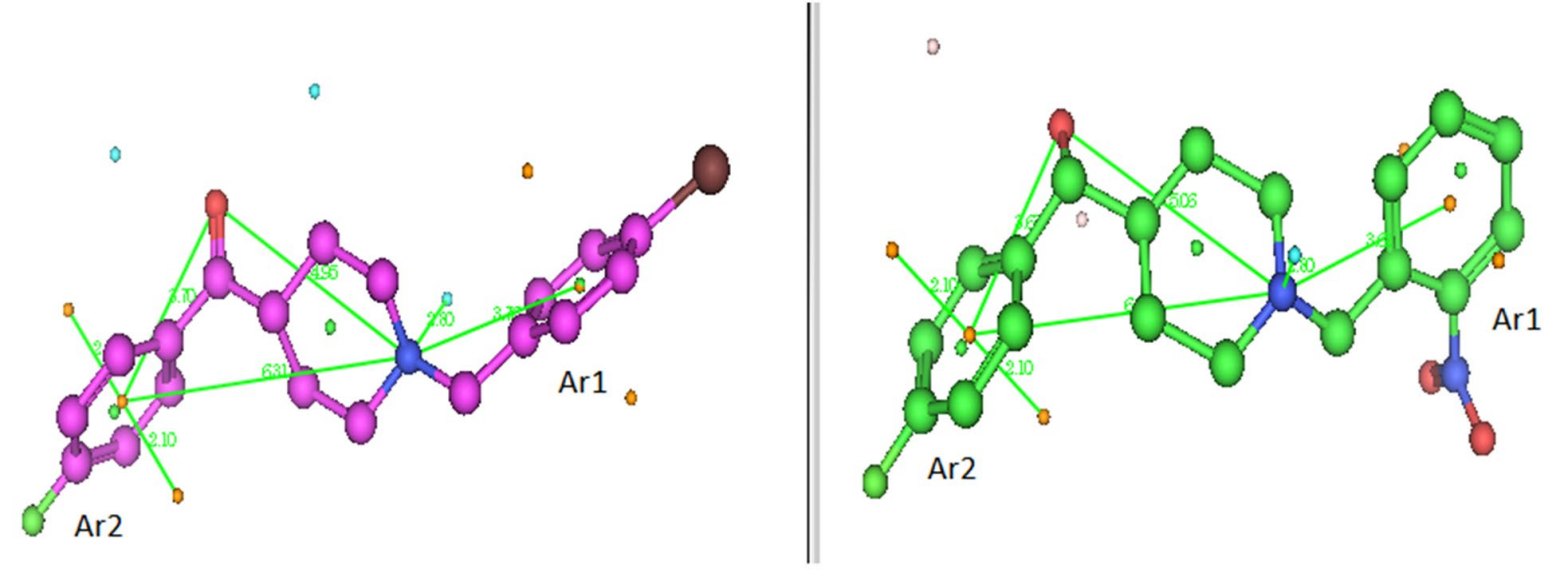
Pharmacophore generation from most potent and least potent σ_1_ ligands

In this study, a comparison between the ph_4_ features generated for the target compounds with the existing ph_4_ model for σ_1_ receptor ligands by Gund et al. [13] was carried out. Gund et al. had proposed that, the distance from the centroid of the primary hydrophobic site to the N atom was 7.14 Å; from the secondary binding site to centroid of the primary hydrophobic site was 3.68 Å and from the secondary binding site to N atom was 4.17 Å. In our model (Fig. 8), the centroids of the primary and secondary hydrophobic sites were chosen from the phenyl groups Ar2 and Ar1, respectively, and the following dimensions were obtained: the distance from the centroid to N atom is 6.30 Å for the most potent ligand and 6.02 Å for the least potent ligand. The distance from O to centroid of the most potent ligand is 3.71 Å and that of the least potent ligand is 3.68 Å; Distance from O to N atom for most potent ligand is 4.97 Å and that for the least potent ligand is 5.06 Å. Therefore, it could be concluded that the distance from the centroid of the primary hydrophobic site to the N atom may vary between 6.30 and 7.14 Å; between 3.68 and 3.71 Å from the secondary binding site to the centroid of the primary hydrophobic site and between 4.17 and 4.97 Å from O to N atom.

### Experimental section

#### Chemistry

The reactions described below were carried out with commercially available chemicals, of reagent grade, that were used without further purification. Reagents were purchased from Sigma-Aldrich Chemical Company, St. Louis, MO, USA. The silica gel (63–200 mesh) which was used as the stationary phase in column chromatography was obtained from Mallinckrodt Baker, Inc. Phillipsburg, New Jersey, USA and Melting points were determined on a Melt—temp II Laboratory device and are uncorrected. All the 1,4-disubstituted piperidine derivatives were converted to their HCl salts by treatment of the corresponding free base with methanolic HCl. Only the HCl salts were submitted for pharmacological evaluation [39–46]. ^1^H and ^13^C NMR spectra were recorded using VARIAN 400 MHz spectrometer (^1^H NMR at 399.75 MHz and ^13^C NMR at 100.53 MHz). Chemical shifts are presented in units of ppm relative to the solvent (^1^H NMR peak: 7.26 ppm for CDCl_3_, 3.3 ppm for CD_3_OD, and ^13^C NMR peak: 49.1 ppm for CD_3_OD and 77.2 ppm for CDCl_3_). Peak multiplicities and characteristics are represented by the following abbreviations: s (singlet), d (doublet), dd (doublet of doublets), t (triplet), q (quartet), m (multiplet). Mass spectra were performed by direct infusion of target compounds. The data was recorded in ESI mode, either ES+ or ES−. TLC analyses were carried out on aluminium plates (Merck) coated with silica gel 60 F254 (0.2 mm thickness). Visualization of spots was performed with UV light and by treatment with iodine. The MS and NMR data are available in the supplementary data (Additional files 1, 2 and 3).

### General method for the preparation of compounds

#### General methods of synthesis for **7a–f**

The synthesis followed the procedure described by Wang et al. [36] with some modification. A mixture containing equimolar quantities (8.6 mmol) of 4-(4-fluorobenzoyl)piperidine hydrochloride, the substituted benzyl chloride in EtOH (15 mL) and NaOAc in distilled water (10 mL) was stirred and heated under reflux overnight. The mixture was allowed to cool to room temperature and concentrated under reduced pressure to provide a residue. The residue was neutralized with a saturated solution of NaHCO_3_ (2 N, 50 mL) and extracted with CH_2_Cl_2_ (2 × 50 mL). The organic extracts were subsequently dried over anhydrous CaCl_2_, concentrated and set aside to give a residue. The product was purified using a short column of silica gel (hexane–ethyl acetate, 3:1). Reaction conditions for compounds: compounds **7a–f** were refluxed at 120 °C, while compounds **8a–f** were refluxed at 60 °C and compounds **9d–f** were refluxed at 120 °C.

#### 4-(4-fluorobenzoyl)-1-[(4-fluorophenyl)methyl]piperidine (**7a**)

Yield [from 4-(4-fluorobenzoyl)piperidine hydrochloride (2.0 g, 8.6 mmol), 4-fluorobenzyl chloride (1.2 g, 8.6 mmol) and NaOAc (1.8 g, 8.6 mmol): sweet smelling shiny cream solid (0.7 g, 51 %). m.p. 103–105 °C. ^1^H NMR (CDCl_3_) δ 1.86 (br. s., 4H, H-3/H-5), 2.17 (br. s., 2H, H_ax_-2/H_ax_-6), 2.97 (d, 2H, J = 11.2 Hz, H_eq_-2/H_eq_-6), 3.22 (br. s., 1H, H-4), 3.54 (br. s., 2H, H-7″), 6.99 (t, 2H, J = 8.5 Hz, H-3′/H-5′), 7.13 (t, 2H, J = 8.5 Hz, H-3″/H-5″), 7.31 (br. s., 2H, H-2″/H-6″), 7.95 (dd, 2H, J = 8.2, 5.7 Hz, H-2′/H-6′). ^13^C NMR (CDCl_3_) δ 28.4 (C-3/C-5), 43.9 (C-4), 52.7 (C-2/C-6), 62.2 (C-7″), 115.2 (C-3′/C-5′), 115.9 (C-3″/C-5″), 130.7 (C-2″/C-6″), 130.8 (C-2′/C-6′), 130.9 (C-1′), 132.4 (C-1″), 164.4 (C-4″), 166.9 (C-4′), 201.0 (C-7′). [TOF MS ES+] calcd for C_19_H_19_F_2_NO *m/z* 315.14, found 338.16 (M + Na)^+^.

#### 1-[(3,4-dichlorophenyl)methyl]-4-(4-fluorobenzoyl)piperidine (**7b**)

Yield [from 4-(4-fluorobenzoyl)piperidine hydrochloride (2.0 g, 8.6 mmol), 3,4-dichlorobenzyl chloride (1.7 g, 8.6 mmol) and NaOAc (1.8 g, 8.6 mmol): sweet smelling shiny white solid (1.4 g, 44 %). m.p. 104–108 °C. ^1^H NMR (CDCl_3_) δ 1.77 (br. s., 4H, H-3/H-5), 2.07 (br. s., 2H, H_ax_-2/H_ax_-6), 2.85 (d, 2H, J = 11.3 Hz, H_eq_-2/H_eq_-6), 3.14 (m, 1H, H-4), 3.41 (s, 2H, H-7″), 7.03–7.15 (m, 3H, H-3′/H-5′, H-6″), 7.31 (d, 1H, J = 8.2 Hz, H-5″), 7.37 (s, 1H, H-2″), 7.89 (dd, 2H, J = 8.4, 5.7 Hz, H-2′/H-6′). ^13^C NMR (CDCl_3_) δ 28.6 (C-3/C-5), 43.5 (C-4), 53.0 (C-2/C-6), 61.8 (C-7″), 115.9 (C-3′/C-5′), 128.1 (C-3″), 130.2 (C-6″), 130.6 (C-5″), 130.8 (C-2′/C-2″), 130.9 (C-1′), 132.3 (C-4″), 132.4 (C-1″), 166.9 (C-4′), 201.0 (C-7′).

#### 1-[(4-chlorophenyl)methyl]-4-(4-fluorobenzoyl)piperidine (**7c**)

Yield [from 4-(4-fluorobenzoyl)piperidine hydrochloride (2.0 g, 8.6 mmol), 4-chlorobenzyl chloride (1.4 g, 8.6 mmol) and NaOAc (1.8 g, 8.6 mmol): sweet smelling shiny white solid (1.4 g, 44 %). m.p. 115–117 °C. ^1^H NMR (CDCl_3_) δ 1.77 (m, 4H, H-3/H-5), 2.05 (br. s., 2H, H_ax_-2/H_ax_-6), 2.87 (d, 2H, J = 11.7 Hz, H_eq_-2/H_eq_-6), 3.13 (m, 1H, H-4), 3.43 (s, 2H, H-7″), 7.06 (t, 2H, J = 8.4 Hz, H-3′/H-5′), 7.21 (m, 4H, H-2″/H-6″, H-3″/H-5″), 7.89 (dd, 2H, J = 8.6, 5.5 Hz, H-2′/H-6′). ^13^C NMR (CDCl_3_) δ 28.7 (C-3/C-5), 43.6 (C-4), 53.0 (C-2/C-6), 62.4 (C-7″), 115.9 (C-3′/C-5′), 128.4 (C-3″/C-5″), 130.3 (C-2′/C-6′), 130.8 (C-1′), 130.9 (C-2″/C-6″), 132.5 (C-1″), 132.7 (C-4″), 166.9 (C-4′), 201.0 (C-7′). [TOF MS ES+] calcd for C_19_H_19_ClFNO *m/z* 331.11, found 354.14 (M + Na)^+^.

#### 4-(4-fluorobenzoyl)-1-[(2-nitrophenyl)methyl]piperidine (**7d**)

Yield [from 4-(4-fluorobenzoyl)piperidine hydrochloride (2.0 g, 8.6 mmol), 2-nitrobenzyl bromide (1.9 g, 8.6 mmol) and NaOAc (1.8 g, 8.6 mmol): sweet smelling shiny brownish-yellow solid (1.3 g, 46 %). m.p. 95–97 °C. ^1^H NMR (CDCl_3_) δ 1.80 (br. s., 4H, H-3/H-5), 2.19 (br. s., 2H, H_ax_-2/H_ax_-6), 2.87 (d, 2H, J = 11.0 Hz, H_eq_-2/H_eq_-6), 3.18 (m, 1H, H-4), 3.80 (s, 2H, H-7″), 7.11 (t, 2H, J = 8.6 Hz, H-3′/H-5′), 7.37 (t, 1H, J = 7.4 Hz, H-4″), 7.53 (t, 1H, J = 7.4 Hz, H-5″), 7.68 (d, 1H, J = 7.4 Hz, H-6″), 7.82 (d, 1H, J = 7.8 Hz, H-3″), 7.94 (dd, 2H, J = 8.6, 5.5 Hz, H-2′/H-6′). ^13^C NMR (CDCl_3_) δ 28.7 (C-3/C-5), 43.4 (C-4), 53.3 (C-2/C-6), 59.0 (C-7″), 115.9 (C-3′/C-5′), 124.3 (C-3″), 127.7 (C-5″), 130.7 (C-6″), 130.8 (C-1′), 130.9 (C-2′/C-6′), 132.4 (C-1″), 132.5 (C-4″), 149.6 (C-2″), 166.9 (C-4′), 201.0 (C-7′). [TOF MS ES +] calcd for C_19_H_19_FN_2_O_3_*m/z* 342.14, found 365.14 (M + Na)^+^.

#### 1-[(4-bromophenyl)methyl]-4-(4-fluorobenzoyl)piperidine (**7e**)

Yield [from 4-(4-fluorobenzoyl)piperidine hydrochloride (2.0 g, 8.6 mmol), 4-bromobenzyl chloride (1.2 g, 8.6 mmol) in EtOH (15 mL) and NaOAc (1.8 g, 8.6 mmol): shiny white solid (0.8 g, 48 %). m.p. 125–126 °C. ^1^H NMR (CDCl_3_) δ 1.77 (m, 4H, H-3/H-5), 2.05 (br. s., 2H, H_ax_-2/H_ax_-6), 2.86 (d, 2H, J = 11.3 Hz, H_eq_-2/H_eq_-6), 3.13 (m, 1H, H-4), 3.41 (s, 2H, H-7″), 7.05 (t, 2H, J = 8.6 Hz, H-3′/H-5′), 7.14 (d, 2H, J = 8.2 Hz, H-3″/H-5″), 7.36 (d, 2H, J = 8.2 Hz, H-2″/H-6″), 7.88 (dd, 2H, J = 8.4, 5.7 Hz, H-2′/H-6′). ^13^C NMR (CDCl_3_) δ 28.7 (C-3/C-5), 43.5 (C-4), 53.0 (C-2/C-6), 62.4 (C-7″), 115.8 (C-3′/C-5′), 120.8 (C-4″), 130.6 (C-2′/C-6′), 130.9 (C-2″/C-6″), 131.3 (C-3″/C-5″), 132.4 (C-1′), 137.4 (C-1″), 166.9 (C-4′), 201.0 (C-7′). [TOF MS ES+] calcd for C_19_H_19_BrFNO *m/z* 375.06, found 400.08 (M + 2 + Na)^+^.

#### 4-(4-fluorobenzoyl)-1-[(4-methylphenyl)methyl]piperidine (**7f**)

Yield [from4-(4-fluorobenzoyl)piperidine hydrochloride (2.0 g, 8.6 mmol), 4- methylbenzyl chloride(1.7 g, 8.6 mmol) and NaOAc (1.8 g, 8.6 mmol): sweet smelling colorless shiny solid (0.7 g, 46 %). m.p. 108–110 °C. ^1^H NMR (CDCl_3_) δ 1.82 (m, 4H, H-3/H-5), 2.09 (td., 2H, J = 10.8, 3.5 Hz, H_ax_-2/H_ax_-6), 2.32 (s, 3H, 4″-C**H**_**3**_), 2.95 (d, 2H, J = 11.7 Hz, H_eq_-2/H_eq_-6), 3.17 (m, 1H, H-4), 3.50 (s, 2H, H-7′), 7.11 (m, 4H, H-3′/H-5′, H-3″/H-5″), 7.20 (d, 2H, J = 7.3 Hz, H-2″/H-6″), 7.94 (dd, 2H, J = 8.4, 5.7 Hz, H-2′/H-6′). ^13^C NMR (CDCl_3_) δ 21.1 (4″-**C**H_3_), 28.7 (C-3/C-5), 43.7 (C-4), 53.0 (C-2/C-6), 62.9 (C-7″), 115.8 (C-3′/C-5′), 128.9 (C-3″/C-5″), 129.1 (C-2″/C-6″), 130.9 (C-2′/C-6′), 132.5 (C-1′), 135.1 (C-1″), 136.6 (C-4″), 166.8 (C-4′), 201.1 (C-7′). [TOF MS ES+] calcd for C_20_H_22_FNO *m/z* 311.17, found 334.16 (M + Na)^+^.

### General method for the preparation of compounds **8a–f**

The synthesis followed the procedure described by Mach et al. [33] with some modification.

Added to a suspension each of **7a–f** in THF (10 mL) was four equivalents of hydrogen from LiBH_4_ all in equimolar quantities in THF (10 mL). The mixture was stirred for 30 min, heated at reflux overnight and allowed to cool to room temperature. The mixture was then concentrated to remove the THF and then treated with distilled water. The organic phase extracted with CH_2_Cl_2_ (2 × 20 mL), washed with brine, dried over CaCl_2_ and evaporated to dryness. The product crystallized spontaneously, was washed with hexane, filtered and air dried.

#### (4-fluorophenyl)({1-[(4-fluorophenyl)methyl]piperidin-4-yl})methanol (**8a**)

Yield [from **7a** (0.4 g, 1.2 mmol), LiBH_4_ (0.03 g, 1.2 mmol). Solid (0.4 g, 97 %). m.p. 133–134 °C. ^1^H NMR (CD_3_OD) δ 1.14 (m, 2H, H_ax_-3/H_ax_-5), 1.29 (m, 2H, H_eq_-3/H_eq_-5), 1.46 (m, 1H, H-4), 1.81 (m, 2H, H_ax_-2/H_ax_-6), 2.71 (d, 1H, J = 11.3 Hz, H_eq_-2), 2.82 (d, 1H, J = 11.3 Hz H_eq_-6), 3.35 (s, 2H, H-7″), 4.20 (d, 1H, J = 7.8 Hz, H-7′), 6.93 (m, 4H, H-3′/H-5′, H-3″/H-5″), 7.20 (m, 4H, H-2′/H-6′, H-2″/H-6″). ^13^C NMR (CDCl_3_) δ 27.7 (C-3), 27.9 (C-5), 43.0 (C-4), 52.9 (C-2), 53.0 (C-6), 61.9 (C-7″), 77.2 (C-7′), 114.3 (C-3″/C-5″), 114.5 (C-3′/C-5′), 128.1 (C-2″/C-6″), 131.1 (C-2′/C-6′), 133.0 (C-1″), 139.5 (C-1′), 160.9 (C-4″), 163.4 (C-4′). [TOF MS ES+] calcd for C_19_H_21_F_2_NO *m/z* 317.16, found 318.18 (M + H)^+^.

#### {1-[(3,4-dichlorophenyl)methyl]piperidin-4-yl}(4-fluorophenyl)methanol (**8b**)

Yield [from **7b** (0.4 g, 1.0 mmol), LiBH_4_ (0.02 g, 1.0 mmol). Solid (0.4 g, 99 %). m.p. 84–86 °C.^1^H NMR (CD_3_OD) δ 1.14 (m, 2H, H_ax_-3/H_ax_-5), 1.27 (m, 2H, H_eq_-3/H_eq_-5), 1.46 (m, 1H, H-4), 1.83 (m, 2H, H_ax_-2/H_ax_-6), 2.69 (d, 1H, J = 11.3 Hz, H_eq_-2), 2.80 (d, 1H, J = 11.3 Hz H_eq_-6), 3.34 (s, 2H, H-7″), 4.21 (d, 1H, J = 7.4 Hz, H-7′), 6.93 (t, 2H, J = 8.6 Hz, H-3′/H-5′), 7.12 (d, 1H, J = 8.2 Hz, H-6″), 7.20 (dd, 2H, H-2′/H-6′), 7.34 (d, 1H, J = 8.2 Hz, H-5″), 7.38 (s, 1H, H-2″). ^13^C NMR (CDCl_3_) δ 29.5 (C-3), 29.6 (C-5), 44.6 (C-4), 54.6 (C-2), 54.8 (C-6), 62.9 (C-7″), 78.8 (C-7′), 115.9 (C-3′/C-5′), 129.7 (C-2′/C-6′), 130.5 (C-3″), 131.5 (C-6″), 132.2 (C-5″), 132.6 (C-2″), 133.3 (C-4″), 140.1 (C-1″), 141.1 (C-1′), 164.8 (C-4′). [TOF MS ES+] calcd for C_19_H_20_Cl_2_FNO *m/z* 367.09, found 388.12 (M + Na)^+^.

#### {1-[(4-chlorophenyl)methyl]piperidin-4-yl}(4-fluorophenyl)methanol (**8c**)

Yield [from **7c** (0.40 g, 1.1 mmol), LiBH_4_ (0.02 g, 1.1 mmol): Solid (0.4 g, 99 %). m.p. 113–116 °C. ^1^H NMR (CD_3_OD) δ 1.15 (m, 2H, H_ax_-3/H_ax_-5), 1.28 (m, 2H, H_eq_-3/H_eq_-5), 1.47 (m, 1H, H-4), 1.84 (m, 2H, H_ax_-2/H_ax_-6), 2.72 (d, 1H, J = 11.3 Hz, H_eq_-2), 2.83 (d, 1H, J = 11.3 Hz, H_eq_-6), 3.37 (s, 2H, H-7″), 4.21 (d, 1H, J = 7.4 Hz, H-7′), 6.93 (t, 2H, J = 8.6 Hz H-3′/H-5′), 7.20 (m, 6H, H-2′/H-6′, H-2″/H-6″, H-3″/H-5″). ^13^C NMR (CDCl_3_) δ 29.3 (C-3), 29.5 (C-5), 44.5 (C-4), 54.6 (C-2), 54.7 (C-6), 63.4 (C-7″), 78.7 (C-7′), 115.8 (C-3′/C-5′), 129.5 (C-3″/C-5″), 129.8 (C-2′/C-6′), 132.5 (C-2″/C-6″), 134.4 (C-4″), 137.3 (C-1″), 141.1(C-1′), 164.8 (C-4′).

#### (4-fluorophenyl)({1-[(2-nitrophenyl)methyl]piperidin-4-yl})methanol (**8d**)

Yield [from **7d** (0.5 g, 2.0 mmol), LiBH_4_ (0.03 g, 2.0 mmol): yellow oil (0.5 g, 98 %). was obtained, washed with hexane and air dried. ^1^H NMR (CD_3_OD) δ 1.08 (m, 2H, H_ax_-3/H_ax_-5), 1.21 (m, 2H, H_eq_-3/H_eq_-5), 1.40 (m, 1H, H-4), 1.83 (m, 2H, H_ax_-2/H_ax_-6), 2.59 (d, 1H, J = 11.0 Hz, H_eq_-2), 2.70 (d, 1H, J = 11.0 Hz, H_eq_-6), 3.60 (s, 2H, H-7″), 4.16 (d, 1H, J = 7.8 Hz, H-7′), 6.92 (t, 2H, J = 8.8 Hz H-3′/H-5′), 7.18 (dd, 2H, J = 8.0, 5.7 Hz, H-2′/H-6′), 7.33 (dt, 1H, J = 8.3, 4.3 Hz, H-4″), 7.46 (d, 2H, J = 4.3 Hz, H-5″/H-6″), 7.68 (d, 1H, J = 7.8 Hz, H-3″). ^13^C NMR (CDCl_3_) δ 29.8 (C-3), 29.9 (C-5), 44.7 (C-4), 54.9 (C-2), 55.0 (C-6), 60.3 (C-7″), 78.9 (C-7′), 116.0 (C-3′/C-5′), 125.4 (C-3″), 129.4 (C-5″), 129.7 (C-2′/C-6′), 132.7 (C-6″), 133.5 (C-1″), 134.8 (C-4″), 141.2 (C-1′), 151.7 (C-2″), 164.8 (C-4′).

#### {1-[(4-bromophenyl)methyl]piperidin-4-yl}(4-fluorophenyl)methanol (**8e**)

Yield [from **7e** (0.5 g, 1.3 mmol), LiBH_4_ (0.03 g, 1.3 mmol): solid (0.4 g, 95 %). m.p. 75–78 °C. ^1^H NMR (CD_3_OD) δ 1.14 (m, 2H, H_ax_-3/H_ax_-5), 1.26 (m, 2H, H_eq_-3/H_eq_-5), 1.46 (m, 1H, H-4), 1.82 (m, 2H, H_ax_-2/H_ax_-6), 2.71 (d, 1H, J = 11.3 Hz, H_eq_-2), 2.82 (d, 1H, J = 11.3 Hz, H_eq_-6), 3.34 (s, 2H, H-7″), 4.20 (d, 1H, J = 7.8 Hz, H-7′), 6.93 (t, 2H, J = 8.8 Hz, H-3′/H-5′), 7.12 (d, 2H, J = 8.2 Hz, H-2″/H-6″), 7.19 (dd, 2H, J = 8.2, 5.5 Hz, H-2′/H-6′), 7.35 (d, J = 8.2 Hz, H-3″/H-5″). ^13^C NMR (CDCl_3_) δ 29.3 (C-3), 29.5 (C-5), 44.5 (C-4), 54.6 (C-2), 54.7 (C-6), 63.5 (C-7″), 78.8 (C-7′), 116.0 (C-3′/C-5′), 122.3 (C-4″), 129.8 (C-2′/C-6′), 132.5 (C-2″/C-6″), 132.8 (C-3″/C-5″), 138.0 (C-1″), 141.1(C-1′), 164.8 (C-4′). [TOF MS ES+] calcd for C_19_H_21_BrFNO *m/z* 377.08, found 378.11 (M + H)^+^.

#### (4-fluorophenyl)({1-[(4-methylphenyl)methyl]piperidin-4-yl})methanol (**8f**)

Yield [from **7f** (0.4 g, 1.2 mmol), LiBH_4_ (0.02 g, 1.2 mmol). Solid (0.4 g, 98 %). m.p. 94–95 °C. ^1^H NMR (CD_3_OD) δ 1.13 (m, 2H, H_ax_-3/H_ax_-5), 1.27 (m, 2H, H_eq_-3/H_eq_-5), 1.45 (m, 1H, H-4), 1.80 (m, 2H, H_ax_-2/H_ax_-6), 2.20 (s, 3H, 4″-C**H**_**3**_), 2.72 (d, 1H, J = 11.3 Hz, H_eq_-2), 2.83 (d, 1H, J = 11.3 Hz, H_eq_-6), 3.33 (s, 2H, H-7″), 4.19 (d, 1H, J = 7.4 Hz, H-7′), 6.93 (t, 2H, J = 8.6 Hz, H-3′/H-5′), 7.01 (d, 2H, J = 7.8 Hz, H-3″/H-5″), 7.06 (d, 2H, J = 7.8 Hz, H-2″/H-6″), 7.19 (dd, J = 8.2, 5.5 Hz, H-2′/H-6′). ^13^C NMR (CDCl_3_) δ 21.3 (4″-**C**H_3_), 29.2 (C-3), 29.4 (C-5), 44.6 (C-4), 54.5 (C-2), 54.6 (C-6), 64.1 (C-7″), 78.8 (C-7′), 116.0 (C-3′/C-5′), 129.8 (C-2′/C-6′), 130.0 (C-2″/C-6″), 131.0 (C-3″/C-5″), 135.1 (C-1″), 138.3 (C-4″), 141.1(C-1′), 164.8 (C-4′). [TOF MS ES+] calcd for C_20_H_24_FNO *m/z* 313.18, found 314.19 (M + H)^+^.

### General method for the preparation of compounds **9d–e**

The synthesis followed the procedure described by Abdel-Magid et al. [34] with some modification. Equimolar quantities of each **7d–e** and 3-bromobenzylamine hydrochloride were weighed in a round bottom flask. Added into the flask was THF (15 mL), equimolar quantity of LiBH_4_ and acetic acid (2 mL). The mixture was stirred and heated under reflux for 3 days and allowed to cool to room temperature. The mixture was then concentrated to remove the THF and then washed with NaHCO_3_ (2 N, 30 mL). The organic phase extracted with CH_2_Cl_2_ (2 × 30 mL) and dried over CaCl_2_ and evaporated to dryness. The product crystallized spontaneously, was washed with hexane, filtered and air dried.

#### [(3-bromophenyl)methyl][(4-fluorophenyl)({1-[(2-nitrophenyl)methyl]piperidin-4-yl})methyl]amine (**9d**)

Yield [from **7d** (0.4 g, 1.0 mmol), 3-bromobenzylamine hydrochloride (0.2 g, 1.0 mmol), LiBH_4_ (0.02 g, 1.0 mmol), AcOH (2 mL), THF (15 mL). Yellow oil (0.4 g, 48 %) was obtained, washed with hexane and air dried. ^1^H NMR (CD_3_OD) δ 1.15 (m, 2H, H_ax_-3/H_ax_-5), 1.31 (m, 2H, H_eq_-3/H_eq_-5), 1.49 (m, 1H, H-4), 1.92 (m, 2H, H_ax_-2/H_ax_-6), 2.68 (d, 1H, J = 11.0 Hz, H_eq_-2), 2.79 (d, 1H, J = 11.0 Hz, H_eq_-6), 3.69 (s, 2H, H-7″), 4.24 (d, 1H, J = 7.4 Hz, H-7′), 4.31 (s, 1H, H_a_-7‴), 4.76 (s, 1H, H_b_-7‴), 7.01 (t, 2H, J = 8.8 Hz, H-3′/H-5′), 7.19-7.44 (m, 7H, H-2′/H-6′, H-4″, H-5″, H-6″, H-5‴, H-6‴), 7.54 (m, 2H, H-3″, H-2‴), 7.77 (d, 1H, J = 8.2 Hz, H-4‴). ^13^C NMR (CDCl_3_) δ 29.9 (C-3/C-5), 44.7 (C-4), 54.9 (C-2), 55.0 (C-6), 60.3 (C-7″), 65.0 (C-7‴), 78.9 (C-7′), 116.0 (C-3′/C-5′), 125.4 (C-3″), 127.5 (C-3‴), 128.0 (C-6‴), 128.5 (C-5‴), 129.4 (C-5″), 129.7 (C-2′/C-6′), 131.4 (C-4‴), 132.2 (C-2‴), 132.7 (C-6″), 133.5 (C-1″), 134.8 (C-4″), 135.2 (C-1‴) 141.2 (C-1′), 151.7 (C-2″), 163.8 (C-4′).

#### [(3-bromophenyl)methyl]({1-[(4-bromophenyl)methyl]piperidin-4-yl}(4-fluorophenyl)methyl)amine (**9e**)

Yield [from **7e** (0.4 g, 1.1 mmol), 3-bromobenzylamine hydrochloride (0.3 g, 1.1 mmol), LiBH_4_ (0.02 g, 1.1 mmol), AcOH (2 mL), THF (15 mL). Yellow oil (0.3 g, 42 %) was obtained, washed with hexane and air dried. ^1^H NMR (CD_3_OD) δ 1.13 (m, 2H, H_ax_-3/H_ax_-5), 1.28 (m, 2H, H_eq_-3/H_eq_-5), 1.47 (m, 1H, H-4), 1.86 (m, 2H, H_ax_-2/H_ax_-6), 3.10 (d, 1H, J = 12.1 Hz, H_eq_-2), 3.18 (d, 1H, J = 12.1 Hz, H_eq_-6), 3.87 (s, 2H, H-7″), 4.09 (s, 1H, H_a_ -7‴), 4.24 (m, 2H, H-7′, H_b_ -7‴), 6.96 (t, 2H, J = 8.8 Hz, H-3′/H-5′), 7.12 (d, 1H, J = 6.7 Hz, H-6‴), 7.21-7.32 (m, 6H, H-2′/H-6′, H-2″/H-6″, H-3″/H-5″), 7.34 (m, 1H, H-5‴), 7.49 (m, 2H, H-2‴, H-4‴). ^13^C NMR (CDCl_3_) δ 27.5 (C-3), 27.8 (C-5), 43.7 (C-4), 52.1 (C-7‴), 53.6 (C-2), 53.8 (C-6), 61.5 (C-7″), 77.7 (C-7′), 116.1 (C-3′/C-5′), 124.4 (C-4″), 127.5 (C-3‴), 128.0 (C-6‴), 128.5 (C-5‴), 129.8 (C-2′/C-6′), 131.7 (C-4‴), 132,1 (C-2‴), 133.1 (C-2″/C-6″), 133.7 (C-1‴), 133.8 (C-3″/C-5″), 134.0 (C-1″), 143.8 (C-1′), 164.9 (C-4′).

#### Sigma receptor binding

These experiments were performed as described by Jinbin et al. [48] with some modification. Different concentrations of test samples were achieved by diluting stock solutions with a solution containing 50 mM Tris–HCl, 150 mM NaCl and 100 mM EDTA at pH 7.4. Rat liver membrane homogenates (~300 μg protein) were diluted with 50 mM Tris–HCl buffer, pH 8.0 and incubated in a total volume of 150 μL with the radioligand at 25 °C in 96 well plates. The incubation time was 60 min for test compounds and 120 min for [^3^H] DTG and [^3^H] (+)-pentazocine.

For determination of sigma 1 binding affinities, the σ_2_ sites were masked in the presence of 1 μM [^3^H]DTG to determine the σ_1_ receptor binding characteristics of [^3^H] (+)-pentazocine while the σ_1_ sites were masked in the presence of 1 μM (+)-pentazocine to determine the σ_2_ receptor binding characteristics of [^3^H]DTG. It is worth mentioning that, this was done one at a time. The final concentration of the radioligand in each assay was ~1 nM for [^3^H] test compounds and ~5 nM for [^3^H] (+)-pentazocine and [^3^H]DTG. Nonspecific binding was determined from samples that contained 10 μM of cold haloperidol.

The reaction was started by adding 0.2 mL of the membrane preparation to the 50 mM Tris–HCl (pH 8.0) buffer containing ^3^H-labeled ligand with a final concentration of 5 nM and cold ligand ranging from 0.01 to 0.1 mM in a final volume of 1.0 mL. Incubations were carried out at 37 °C for 150 min in the binding study with [^3^H] (+)-pentazocine and at 25 °C for 90 min in the study with [^3^H] DTG. Inhibitor concentrations ranging from 0.1 nM to 10 μM were added to acquire the inhibition curves. After the reaction was completed, the samples were harvested, washed three times, and the bound radioactivity counted and analyzed. Data from the competitive inhibition experiments were modeled using nonlinear regression analysis to determine the concentration of inhibitor that inhibits 50 % of the specific binding of the radioligand (IC_50_ value) and the competitive inhibition constants (K_i_ values) were calculated from the IC_50_.

### Computational methodology

All molecular modeling was carried out using the software, MOE [49]. Initially, each compound was sketched using the Builder module of MOE package. Energy minimization was carried out using the MOPAC module of MOE at the AM1 level of theory using a minimization gradient of 0.001 kcal/mol. For compounds with chiral centres, only the *R*-isomers were considered. In the generation of MEPs, the cut-offs were set at 1.62 Å. Pharmacophore models were generated using the polarity-charge-hydrophobicity (PCH) scheme implemented in MOE. The binding sites were defined by mapping the topographical arrangement of the phenyl rings, N-atoms and the electronegative atoms as described by Gund et al. [13].

The binding affinities to the σ_1_ receptor were computed using Eq. 4:

4 $$\Delta G^{\exp } = - RT \ln K_{i}$$

where *R* is the ideal gas constant and *T* is the absolute temperature. The residual binding affinities were computed as:

5 $$\Delta G^{res} = \Delta G^{\exp } - \Delta G^{pred}$$

These values give a measure of the error estimates for individual values calculated by the regression equation for the dataset. Similarly, the residual values for experimental and predicted activities were obtained from the difference between $pIC_{50}^{\exp }$ and $pIC_{50}^{{\text{pred}}}$. This value gives a measure of the error in estimates for individual values calculated by the regression equation for the data set.

## Conclusions

The replacement of spirofusion in the lead compound **1** by either a hydroxymethylene or carbonyl bridge led to 4-aroylpiperidines and 4-(α-hydroxyphenyl)piperidines. Most of the compounds have high affinity for σ_1_ receptors and fit well into the Gund’s pharmacophore model for σ_1_ receptor ligands; they also display poor affinity for σ_2_ receptors, and finally, some of them have a higher selectivity for the σ_1_ receptor compared to the lead compound **1**. Thus, spirofusion confers no particular advantage in **1** over its ring open analogues. These analogues with secondary binding sites like H-bond acceptors as well as H-bond donors both emerged as potent σ_1_ receptor ligands. Therefore, the secondary binding site proposed by Lu et al. [7], may either be a H-bond donor or acceptor. Following the ph4 models generated in this study, potential σ_1_ binders could be virtually screened from our recently developed natural product libraries from African medicinal plants [50–53].

## Additional files

10.1186/s13065-016-0200-1 MS data for synthesized compounds.
10.1186/s13065-016-0200-1 NMR data for synthesized compounds (part 1).
10.1186/s13065-016-0200-1 NMR data for synthesized compounds (part 2).
