# Supplementary material for: 4-aroylpiperidines and 4-(α-hydroxyphenyl)piperidines as selective sigma-1 receptor ligands: synthesis, preliminary pharmacological evaluation and computational studies
Source: Chem Cent J. 2016 Aug 23;10(1):53. doi: 10.1186/s13065-016-0200-1 (PMC4994268; doi:10.1186/s13065-016-0200-1)
Supplement: Supplementary file 1 — 10.1186/s13065-016-0200-1 MS data for synthesized compounds. [file 13065_2016_200_MOESM1_ESM.docx]

**4-Aroylpiperidines and 4-(α-Hydroxyphenyl)piperidines as Selective Sigma-1 Receptor Ligands: Synthesis, Preliminary Pharmacological Evaluation and Computational Studies.**

Hermia Nalova Ikome^a^,

Email: ikomenash@yahoo.com

Fidele Ntie-Kang^bc*^,

Email: ntiekfidele@gmail.com

Moses N. Ngemenya^d^,

Email: mnngemenya@yahoo.com

Zhude Tu^e^,

Email: tuz@mir.wustl.edu

Robert H. Mach^e^,

Email: rmach@mail.med.upenn.edu

Simon M. N. Efange^a*^

Email: smbuangalefange@gmail.com

*^a^Department of Chemistry, Faculty of Science, University of Buea, P.O.Box 63,Buea, South West Region, Cameroon,*

*^b^Department of Chemistry, University of Buea, P. O. Box 63, Buea, Cameroon,*

*^c^Department of Pharmaceutical Chemistry, Martin-Luther University of Halle-Wittenberg, Wolfgang-Langenbeck-Str. 4, 06122 Halle (Saale), Germany;*

*^d^Biotechnology Unit, Department of Biochemistry and Molecular Biology, Faculty of Science, University of Buea, P.O.Box 63, South West Region, Cameroon;*

*^e^Department of Radiology, University of Washington University School of Medicine, USA*

*^*^Corresponding authors (FNK and SMNE)*

SUPPLEMENTARY FILES

Table 1: Correspondence between compound numbering as in manuscript (7a, 7b, .... 8c, 8d, .... 9d and 9e) and those of codes of compounds subject to analysis (e.g. SE_UB-22_45)

| SE_UB-22_45=7a | SE_UB-22_52=8c | SE_UB-22_60=9d |
| --- | --- | --- |
| SE_UB-22_47=7b | SE_UB-22_53=8d | SE_UB-22_61=9e |
| SE_UB-22_48=7c | SE_UB-22_54=8e |  |
| SE_UB-22_49=7d | SE_UB-22_55=8b |  |
| SE_UB-22_50=7e | SE_UB-22_56=8a |  |
| SE_UB-22_51=7f | SE_UB-22_57=8f |  |

MS DATA

7a

7d

7e

7f

8a

8b

8c

8e

8f
