# Supplementary material for: 4-aroylpiperidines and 4-(α-hydroxyphenyl)piperidines as selective sigma-1 receptor ligands: synthesis, preliminary pharmacological evaluation and computational studies
Source: Chem Cent J. 2016 Aug 23;10(1):53. doi: 10.1186/s13065-016-0200-1 (PMC4994268; doi:10.1186/s13065-016-0200-1)
Supplement: Supplementary file 2 — 10.1186/s13065-016-0200-1 NMR data for synthesized compounds (part 1). [file 13065_2016_200_MOESM2_ESM.pdf]

SE_UB-22_45
Nucleus: 1H
Frequency: 399.75
Pulse Sequence: s2pul
Solvent: cdcl3
Title: PROTON_01
Acquisition Date: 2012-06-05T09:41:42

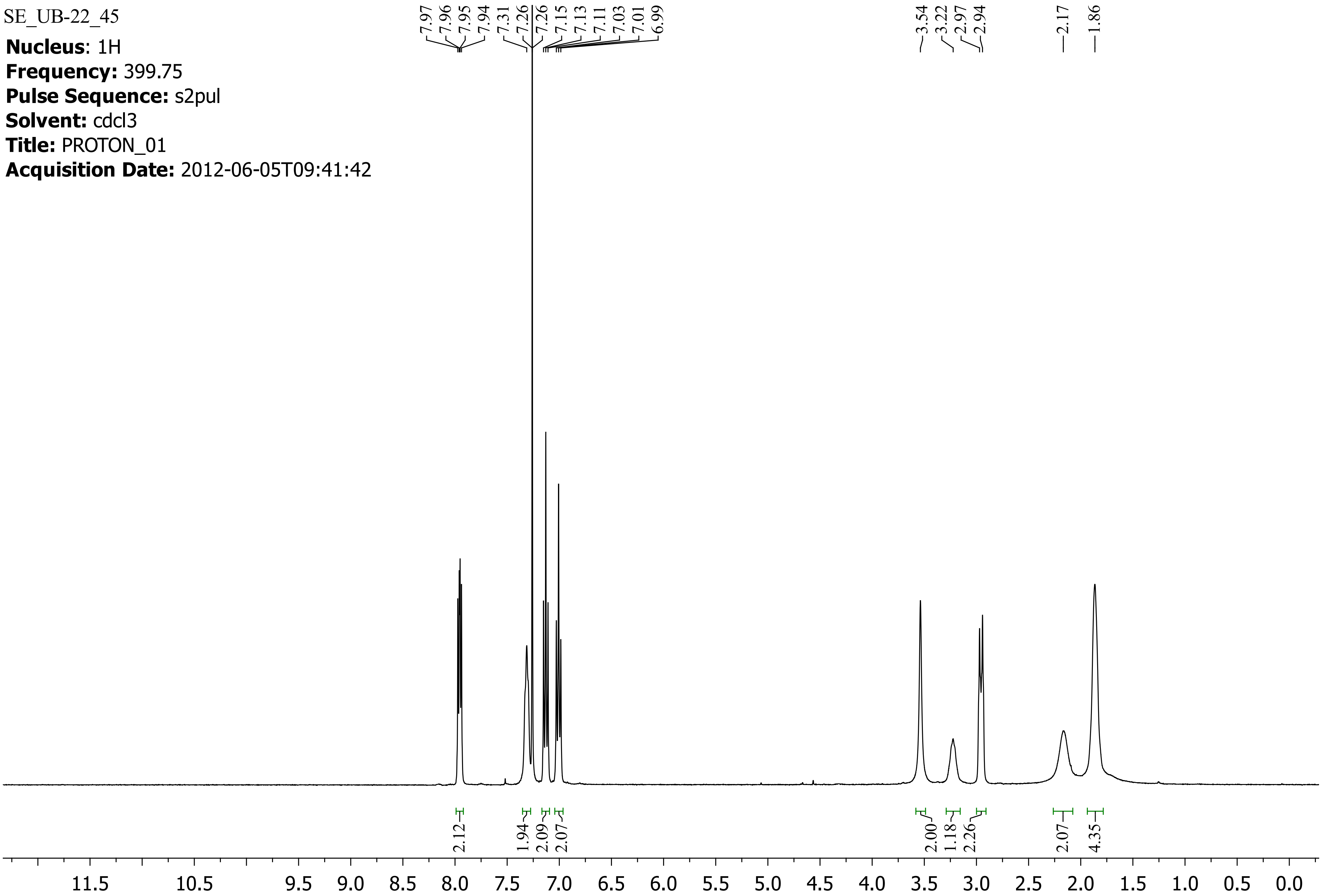

SE_UB-22_45
Nucleus: 1H
Frequency: 399.75
Pulse Sequence: s2pul
Solvent: cdcl3
Title: PROTON_01
Acquisition Date: 2012-06-05T09:41:42

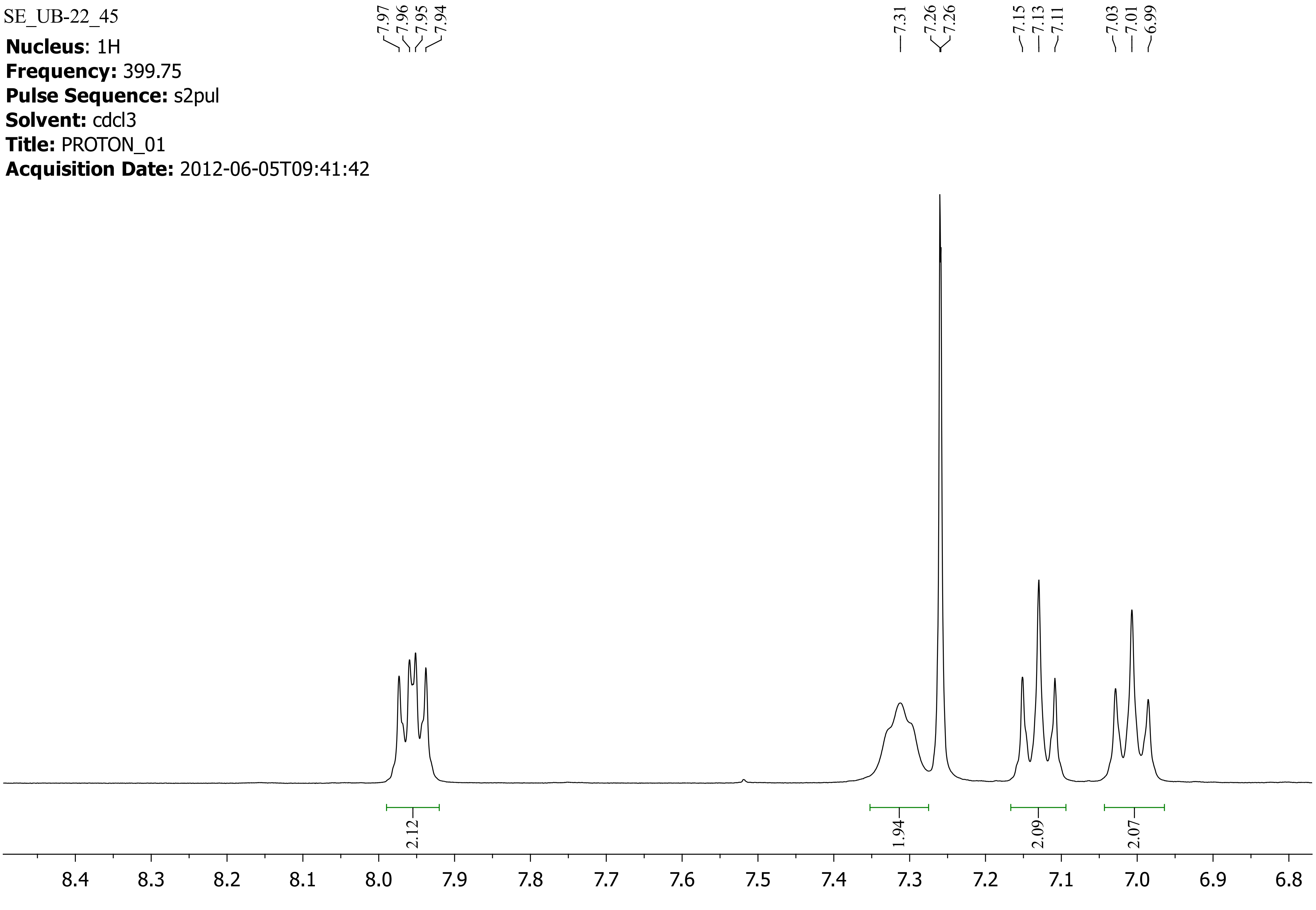

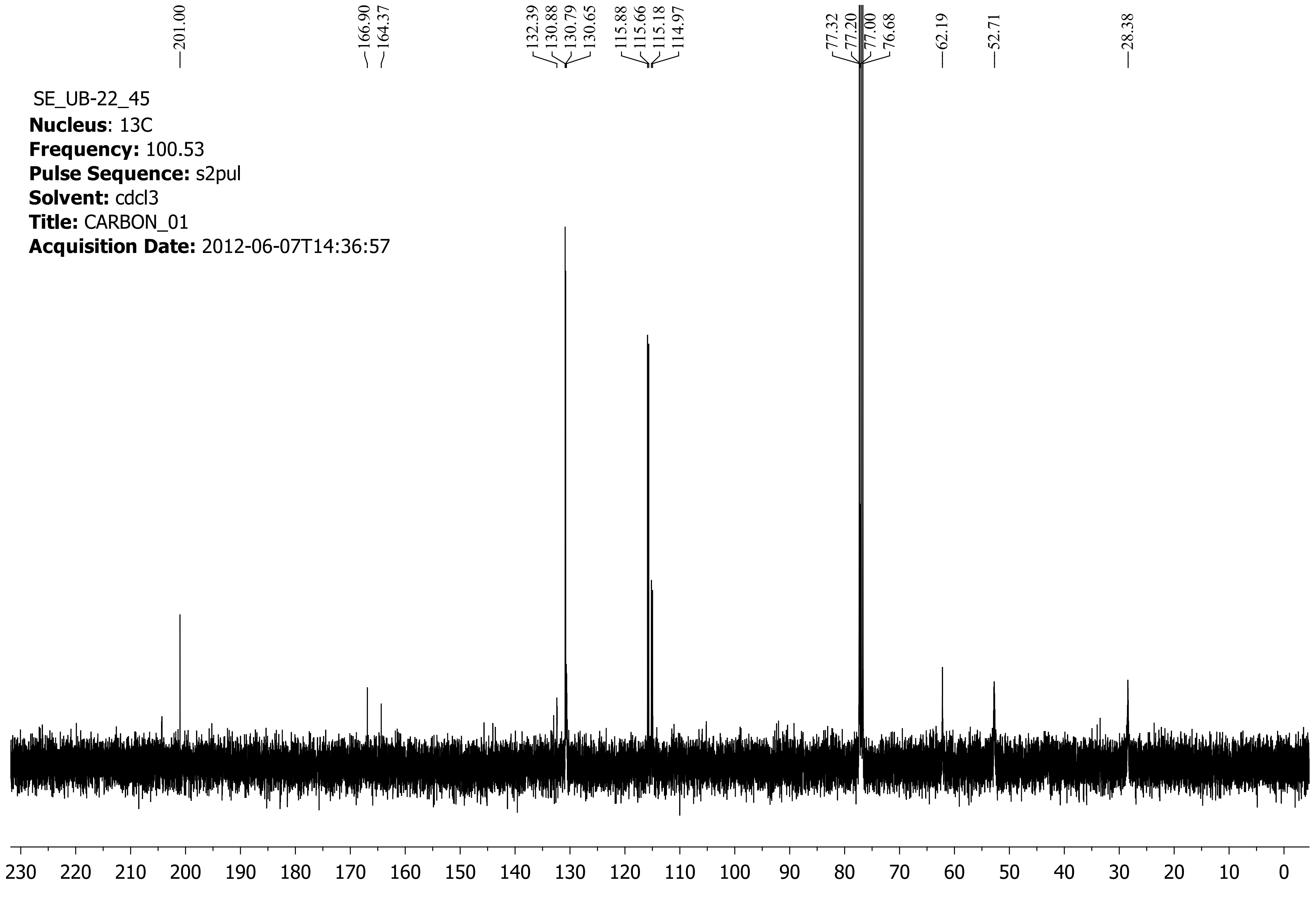
201.00
166.90
164.37
132.39
130.88
130.79
130.65
115.88
115.66
115.18
114.97
77.32
77.20
77.00
76.68
62.19
52.71
28.38
SE_UB-22_45
Nucleus: 13C
Frequency: 100.53
Pulse Sequence: s2pul
Solvent: cdcl3
Title: CARBON_01
Acquisition Date: 2012-06-07T14:36:57
230 220 210 200 190 180 170 160 150 140 130 120 110 100 90 80 70 60 50 40 30 20 10 0

SE_UB-22_47
Nucleus: 1H
Frequency: 399.75
Pulse Sequence: s2pul
Solvent: cdcl3
Title: PROTON_01
Acquisition Date: 2012-06-18T16:56:33

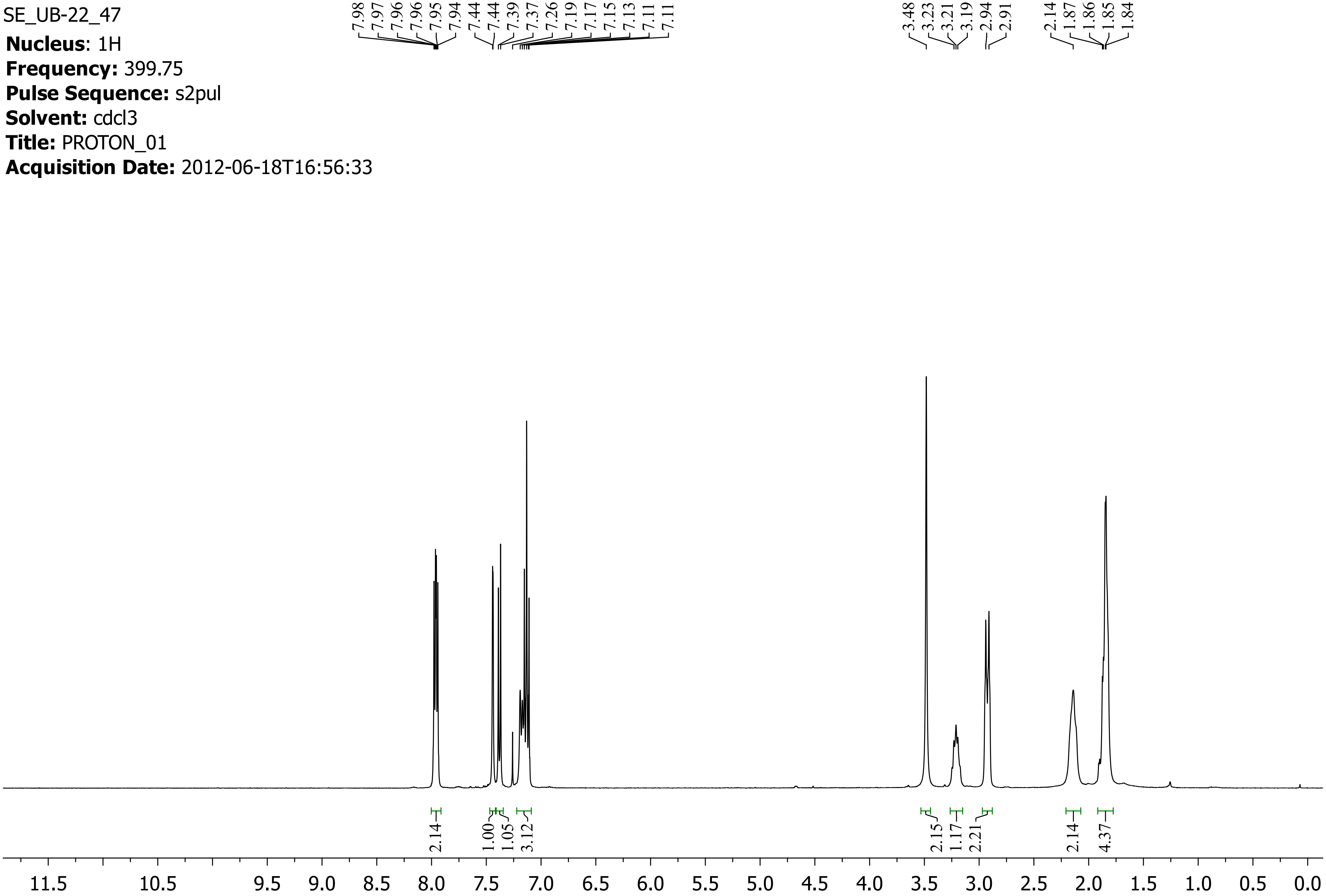

SE_UB-22_47
Nucleus: 1H
Frequency: 399.75
Pulse Sequence: s2pul
Solvent: cdcl3
Title: PROTON_01
Acquisition Date: 2012-06-18T16:56:33

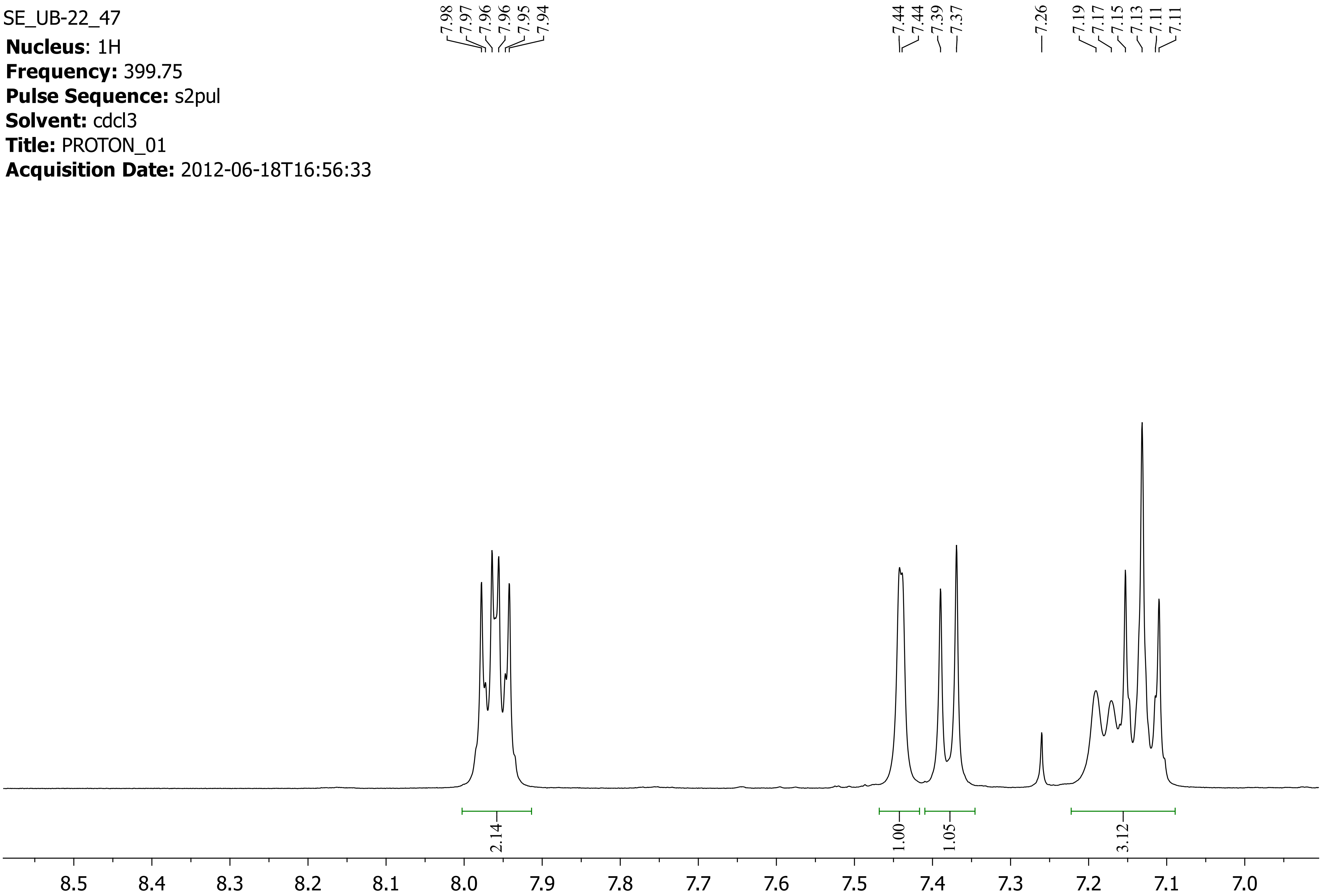

SE_UB-22_47

Nucleus: 13C
Frequency: 100.53
Pulse Sequence: s2pul
Solvent: cdcl3
Title: CARBON_01
Acquisition Date: 2012-06-18T16:57:12

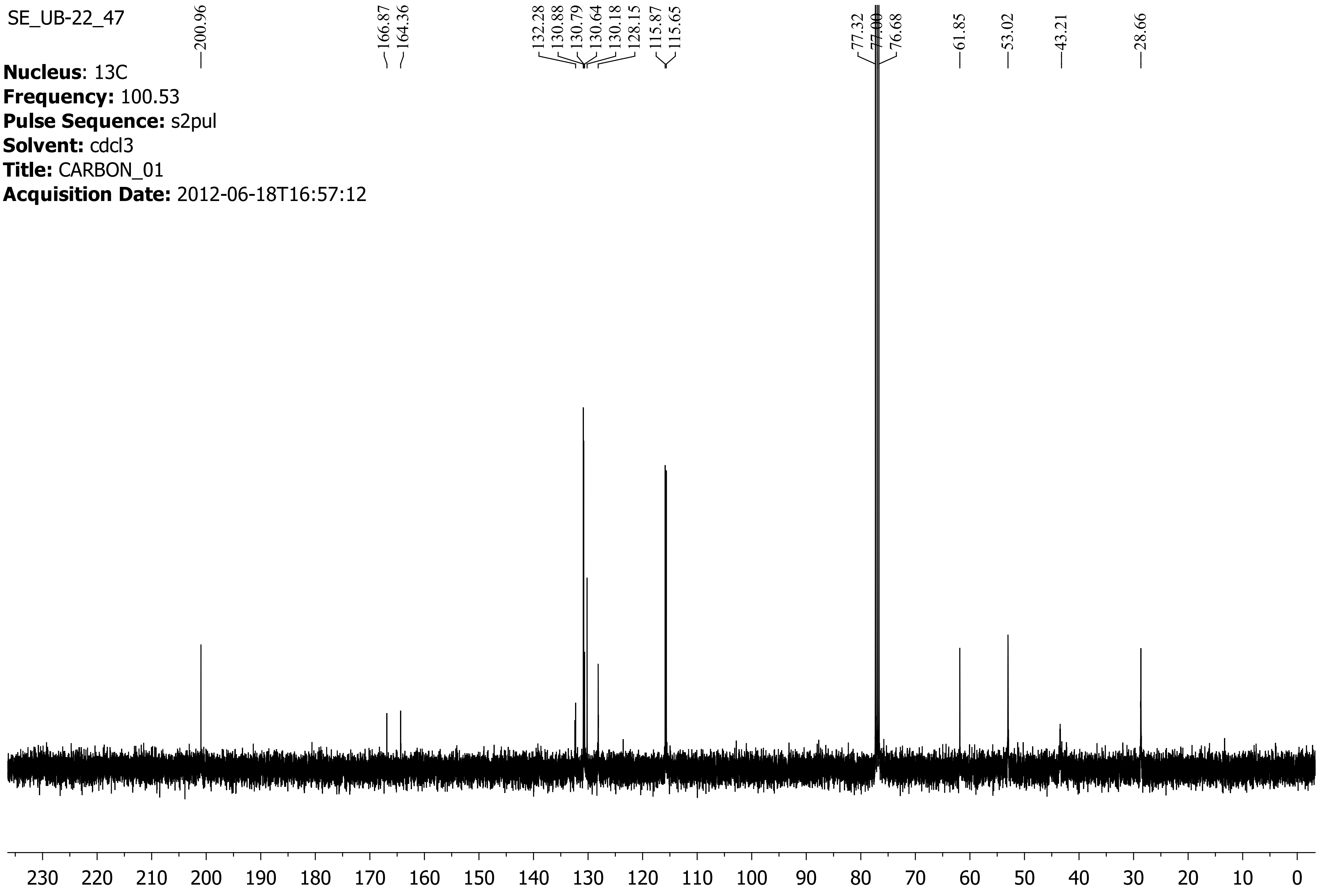

SE_UB-22_48
Nucleus: 1H
Frequency: 399.75
Pulse Sequence: s2pul
Solvent: cdcl3
Title: PROTON_01
Acquisition Date: 2012-06-18T17:11:08

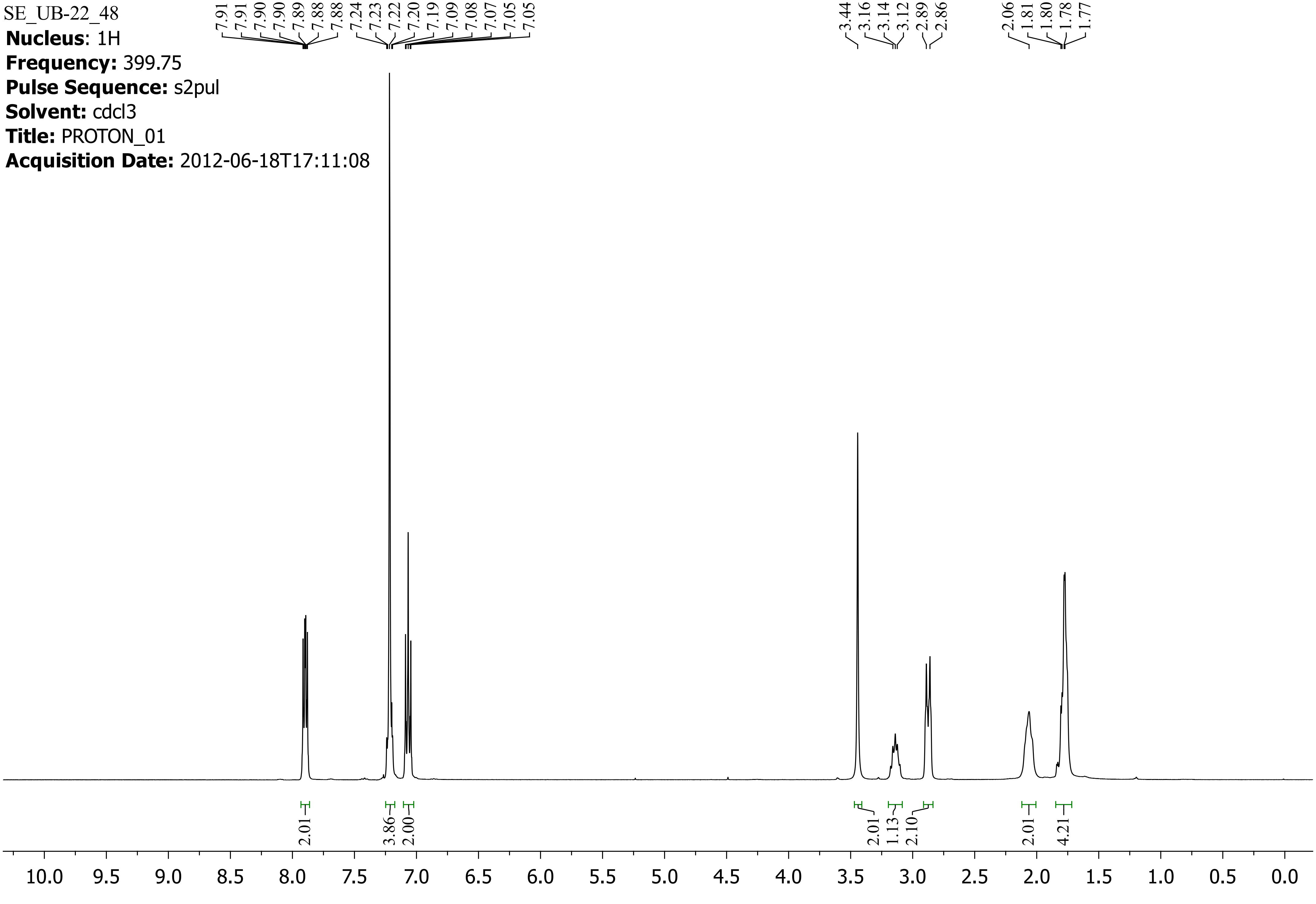

SE_UB-22_48
Nucleus: 1H
Frequency: 399.75
Pulse Sequence: s2pul
Solvent: cdcl3
Title: PROTON_01
Acquisition Date: 2012-06-18T17:11:08

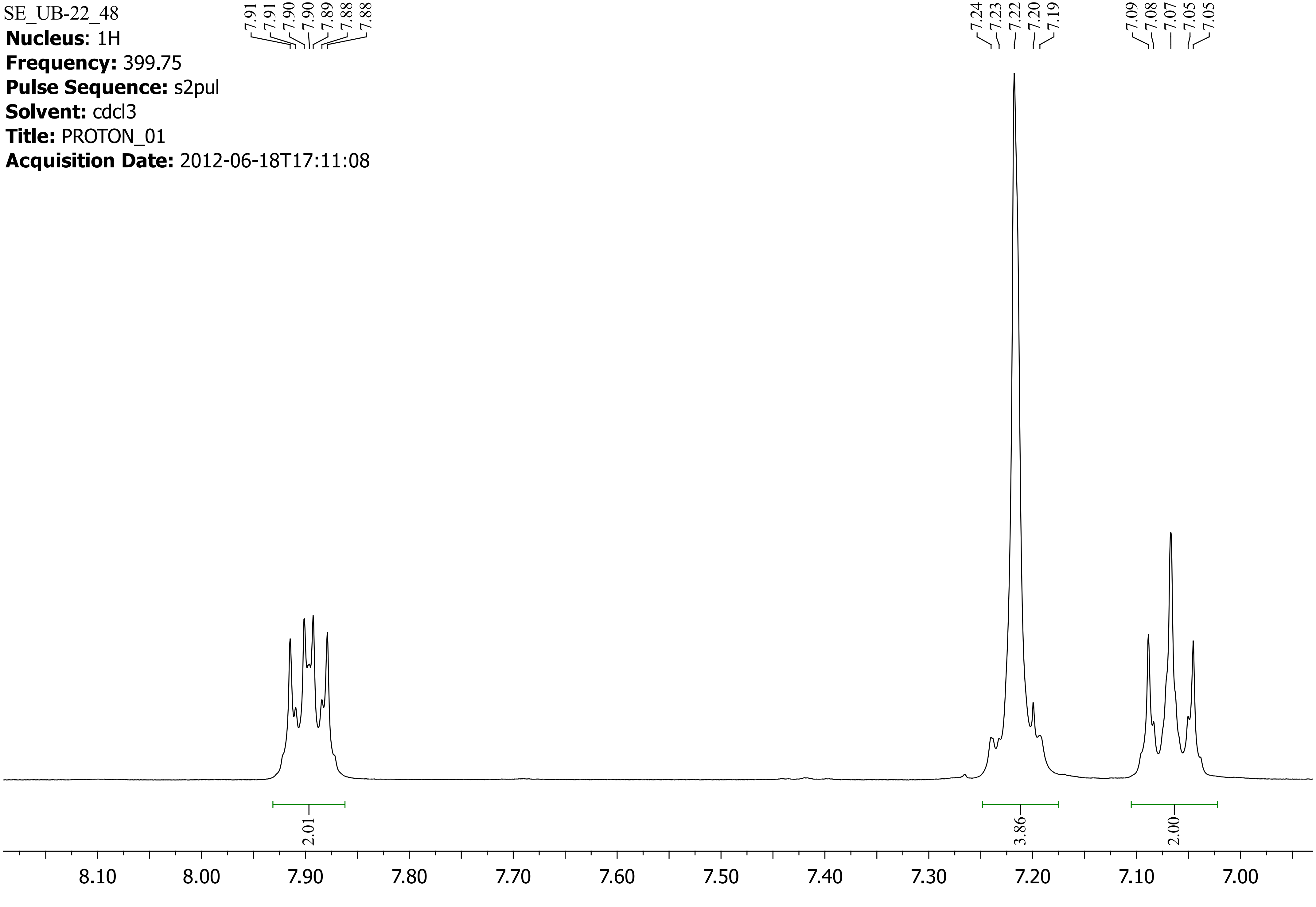

SE_UB-22_48

Nucleus: 13C
Frequency: 100.53
Pulse Sequence: s2pul
Solvent: cdcl3
Title: CARBON_01
Acquisition Date: 2012-06-18T17:11:47

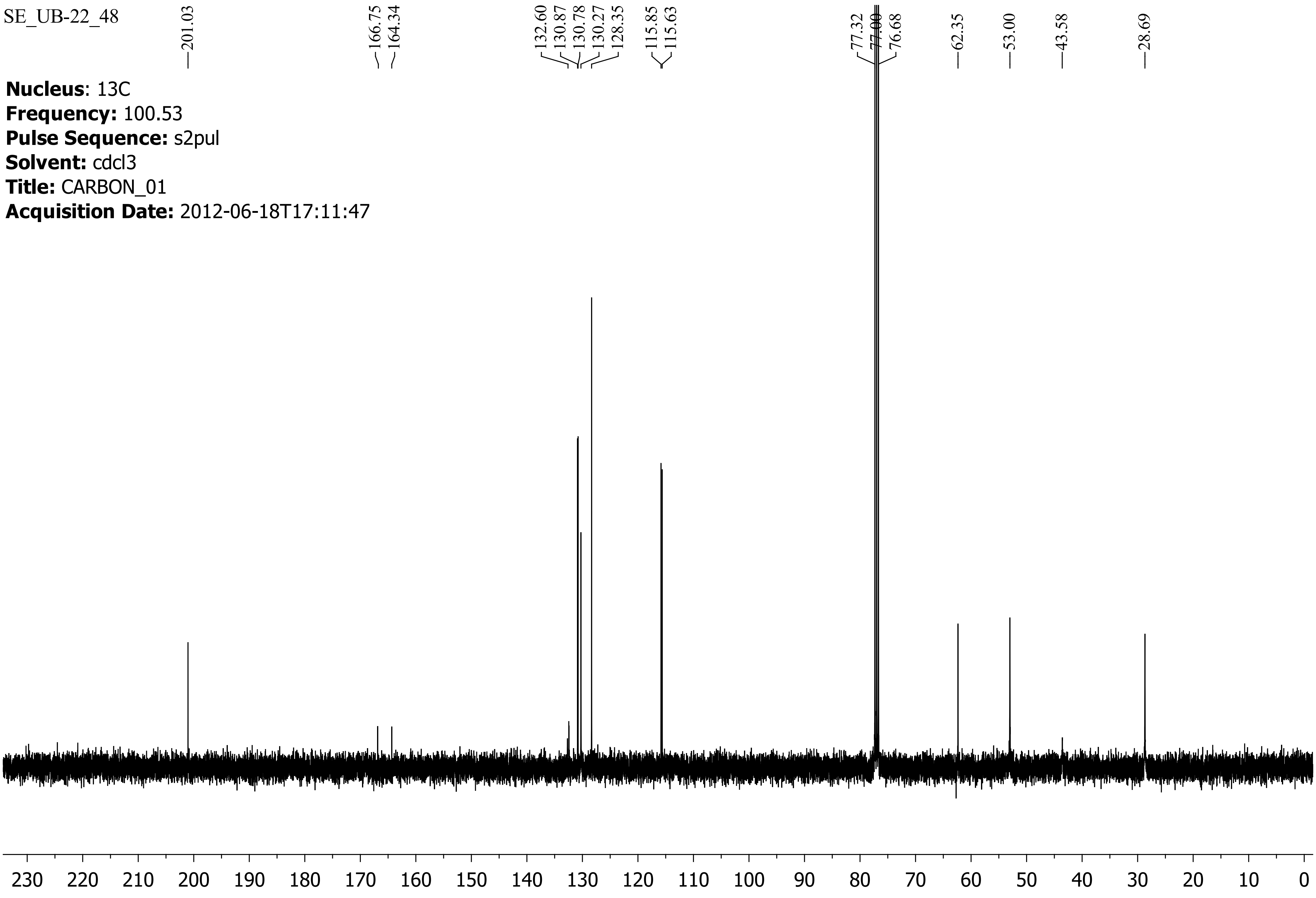

SE_UB-22_49

Nucleus: 1H
Frequency: 399.75
Pulse Sequence: s2pul
Solvent: cdcl3
Title: PROTON_01
Acquisition Date: 2012-06-18T17:25:17

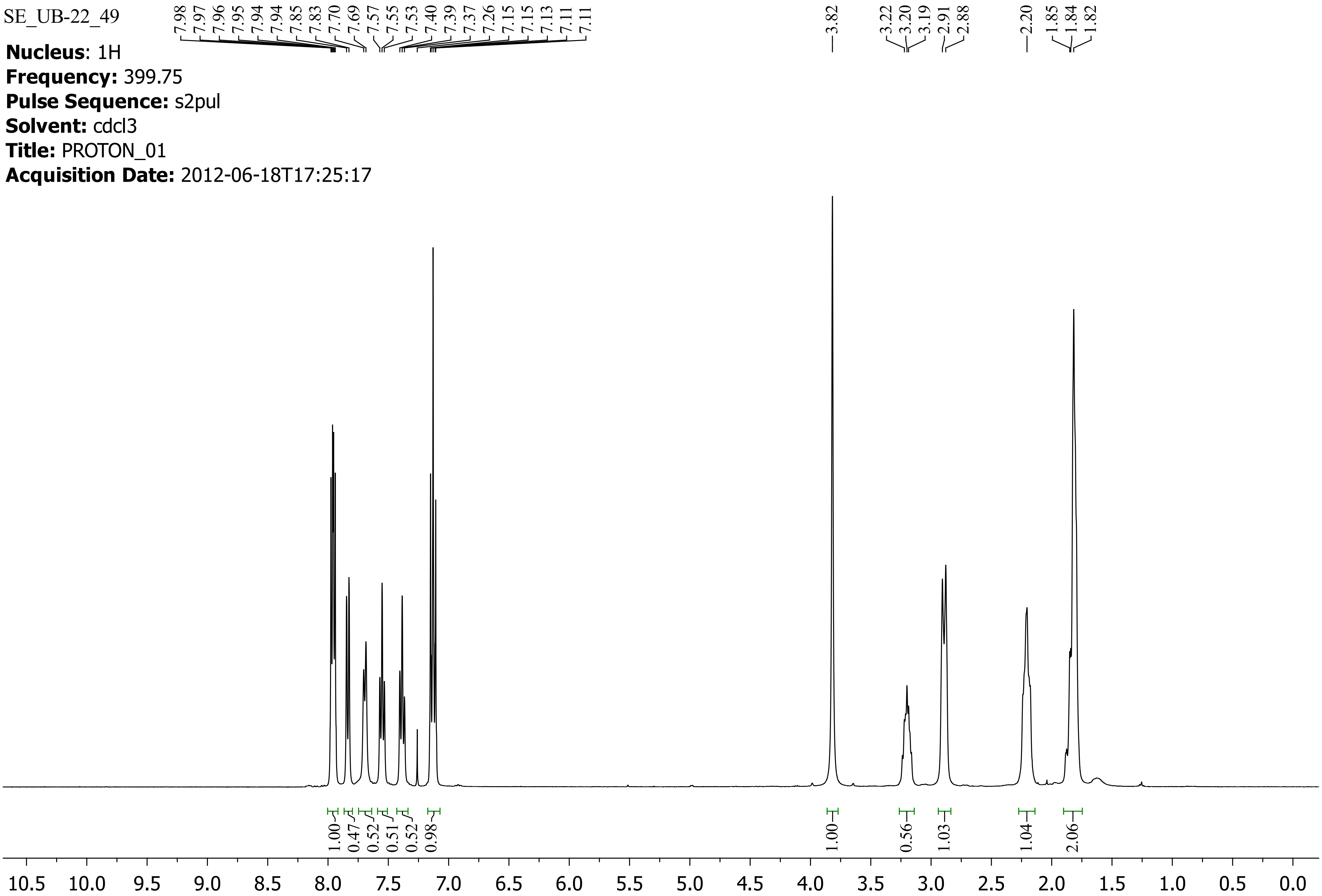

SE_UB-22_49

Nucleus: 1H
Frequency: 399.75
Pulse Sequence: s2pul
Solvent: cdcl3
Title: PROTON_01
Acquisition Date: 2012-06-18T17:25:17

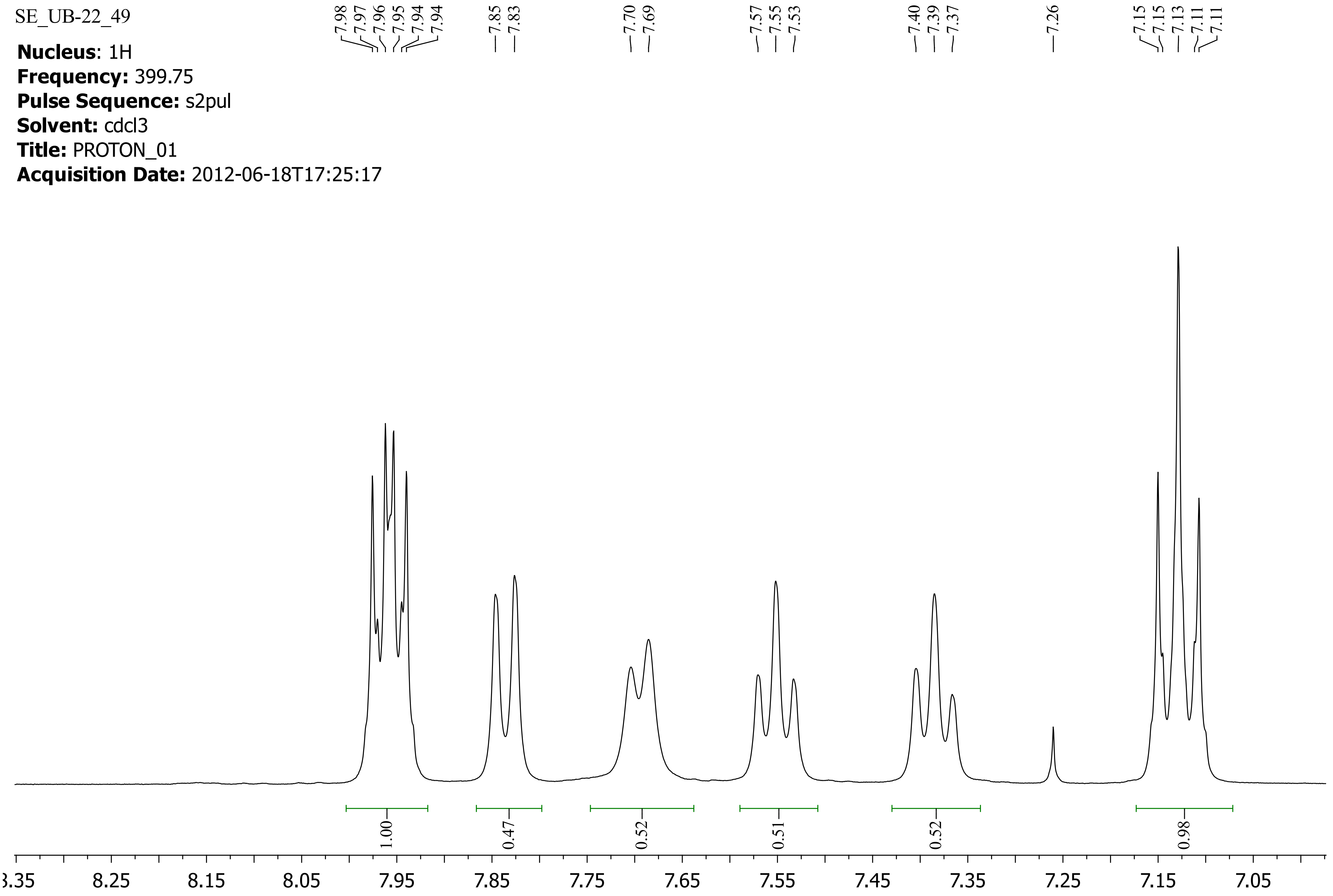

SE_UB-22_49

Nucleus: 13C
Frequency: 100.53
Pulse Sequence: s2pul
Solvent: cdcl3
Title: CARBON_01
Acquisition Date: 2012-06-18T17:25:56

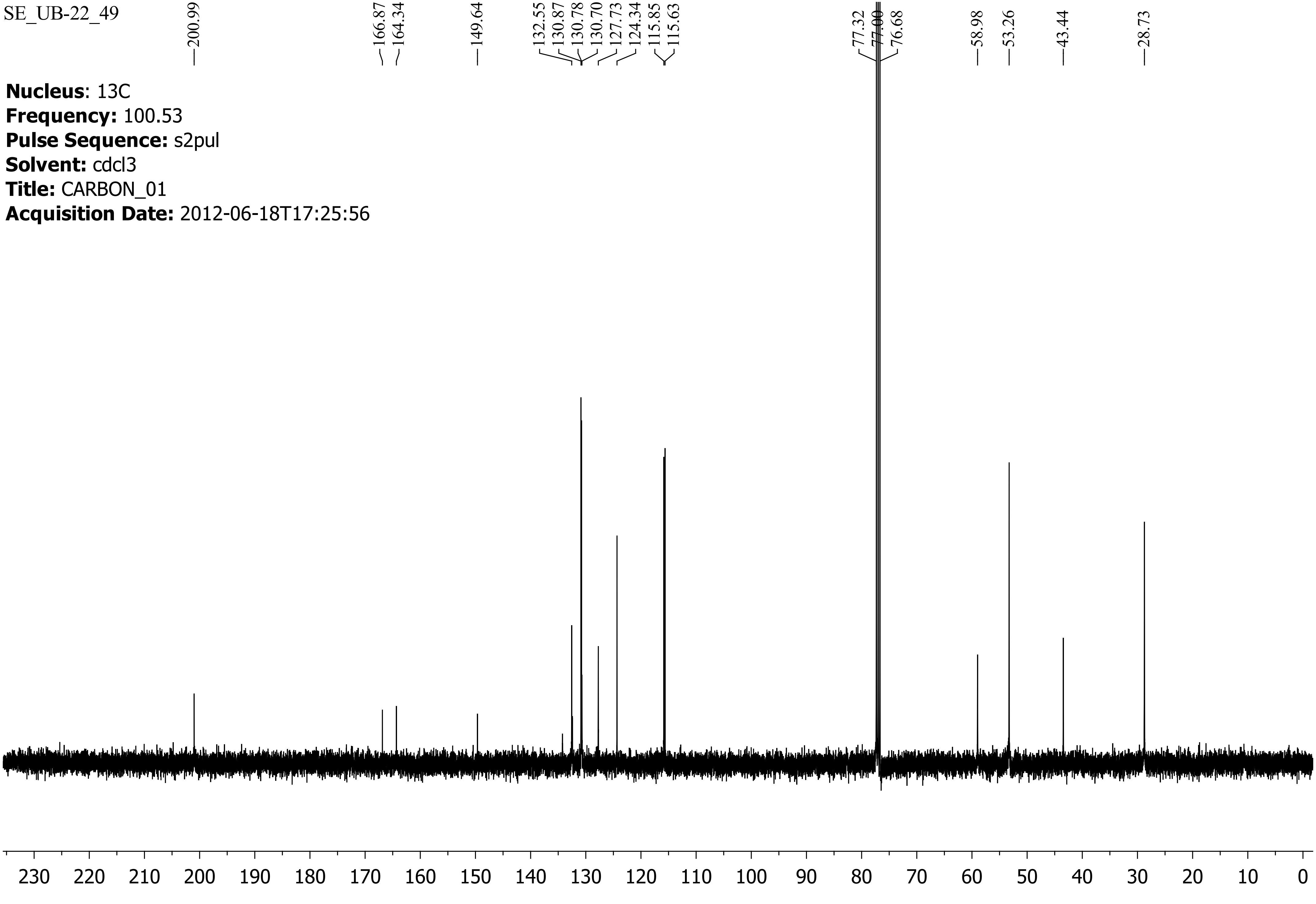

SE_UB-22_50
Nucleus: 1H
Frequency: 399.75
Pulse Sequence: s2pul
Solvent: cdcl3
Title: PROTON_01
Acquisition Date: 2012-06-18T17:39:27

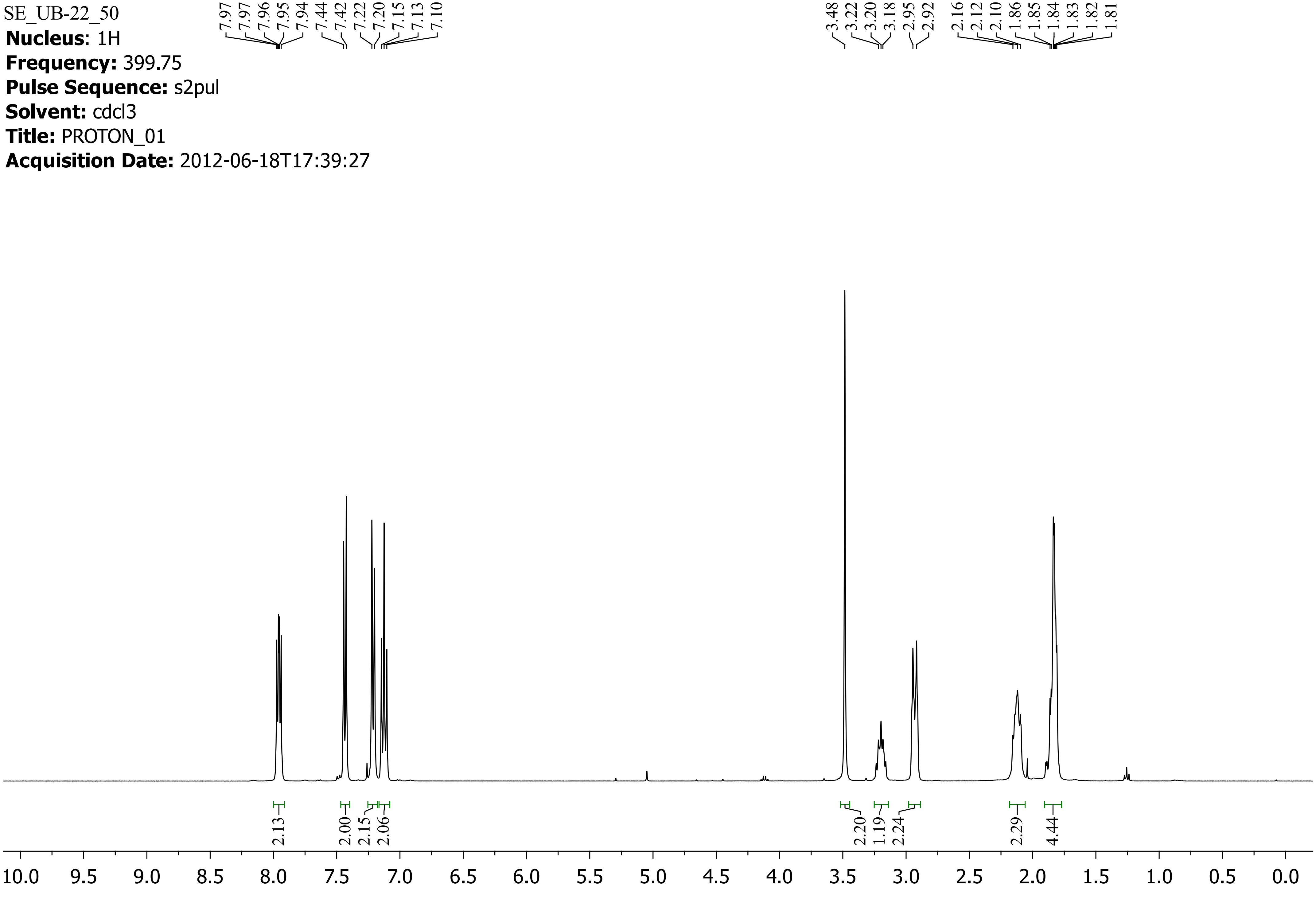

SE_UB-22_50
Nucleus: 1H
Frequency: 399.75
Pulse Sequence: s2pul
Solvent: cdcl3
Title: PROTON_01
Acquisition Date: 2012-06-18T17:39:27

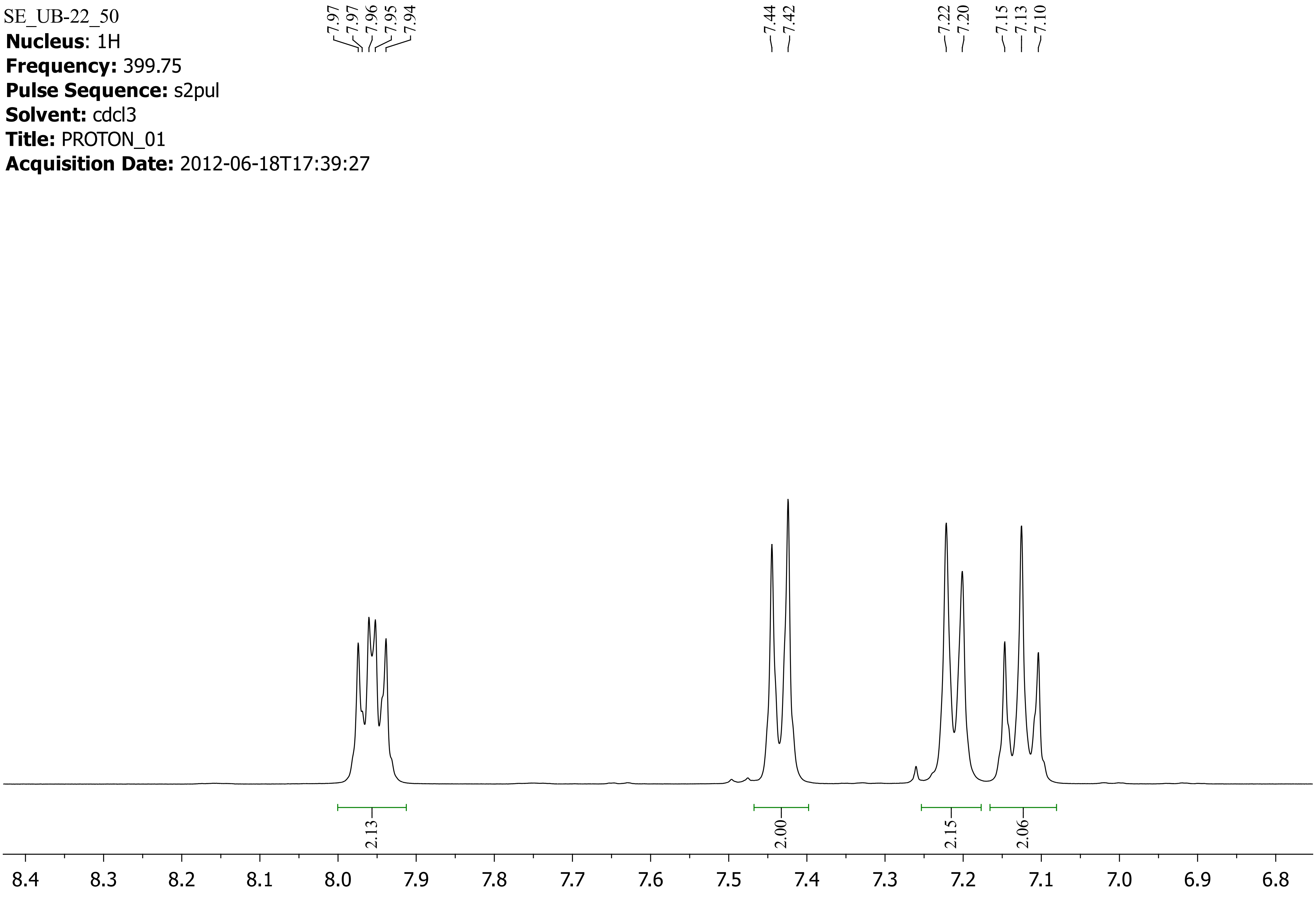

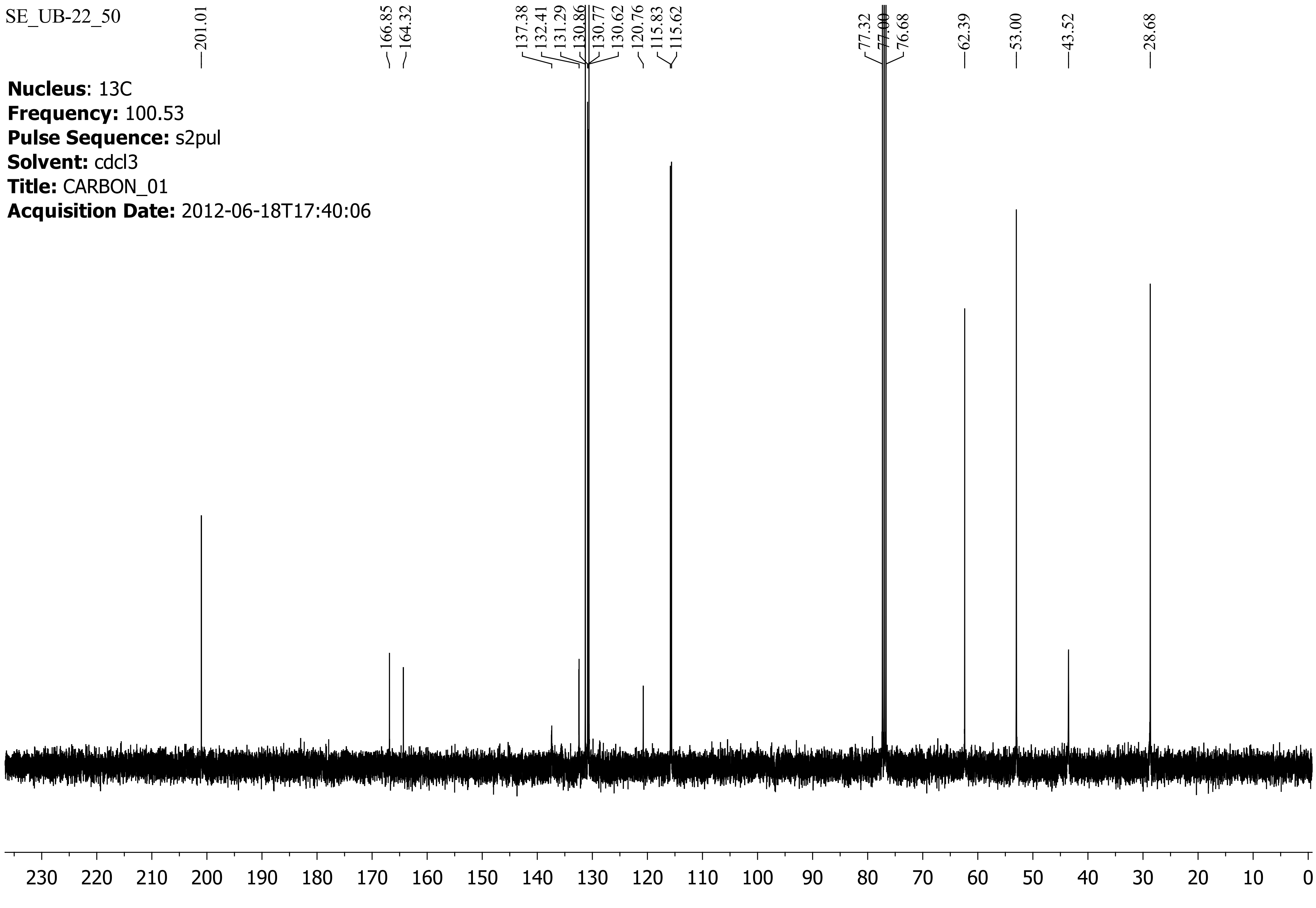
SE_UB-22_50
Nucleus: 13C
Frequency: 100.53
Pulse Sequence: s2pul
Solvent: cdcl3
Title: CARBON_01
Acquisition Date: 2012-06-18T17:40:06
201.01
166.85
164.32
137.38
132.41
131.29
130.86
130.77
130.62
120.76
115.83
115.62
77.32
77.00
76.68
62.39
53.00
43.52
28.68
230 220 210 200 190 180 170 160 150 140 130 120 110 100 90 80 70 60 50 40 30 20 10 0

SE_UB-22_50

Nucleus: 13C
Frequency: 100.53
Pulse Sequence: s2pul
Solvent: cdcl3
Title: CARBON_01
Acquisition Date: 2012-06-18T17:40:06

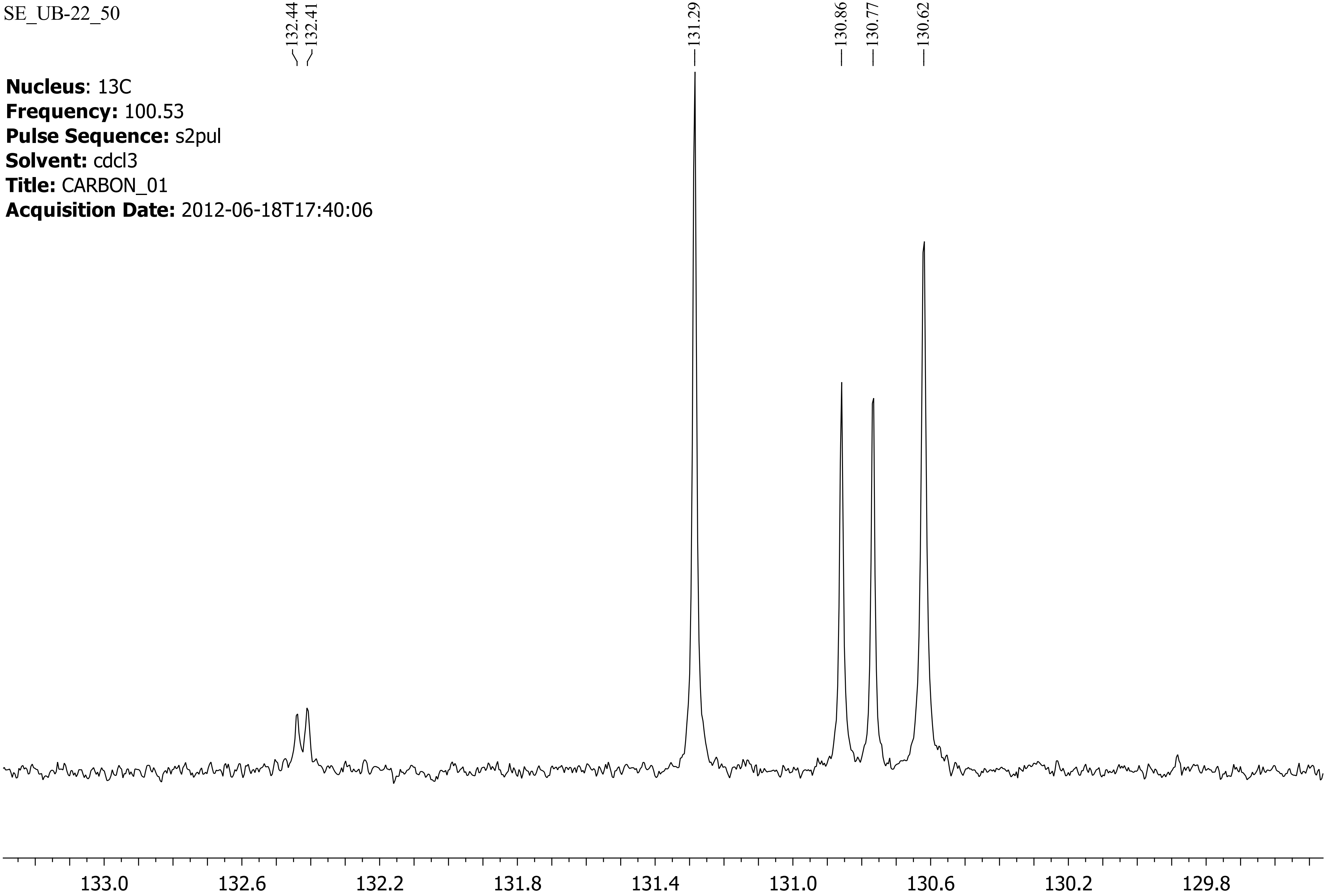

SE_UB-22_51

Nucleus: 1H
Frequency: 399.75
Pulse Sequence: s2pul
Solvent: cdcl3
Title: PROTON_01
Acquisition Date: 2012-06-19T13:19:47

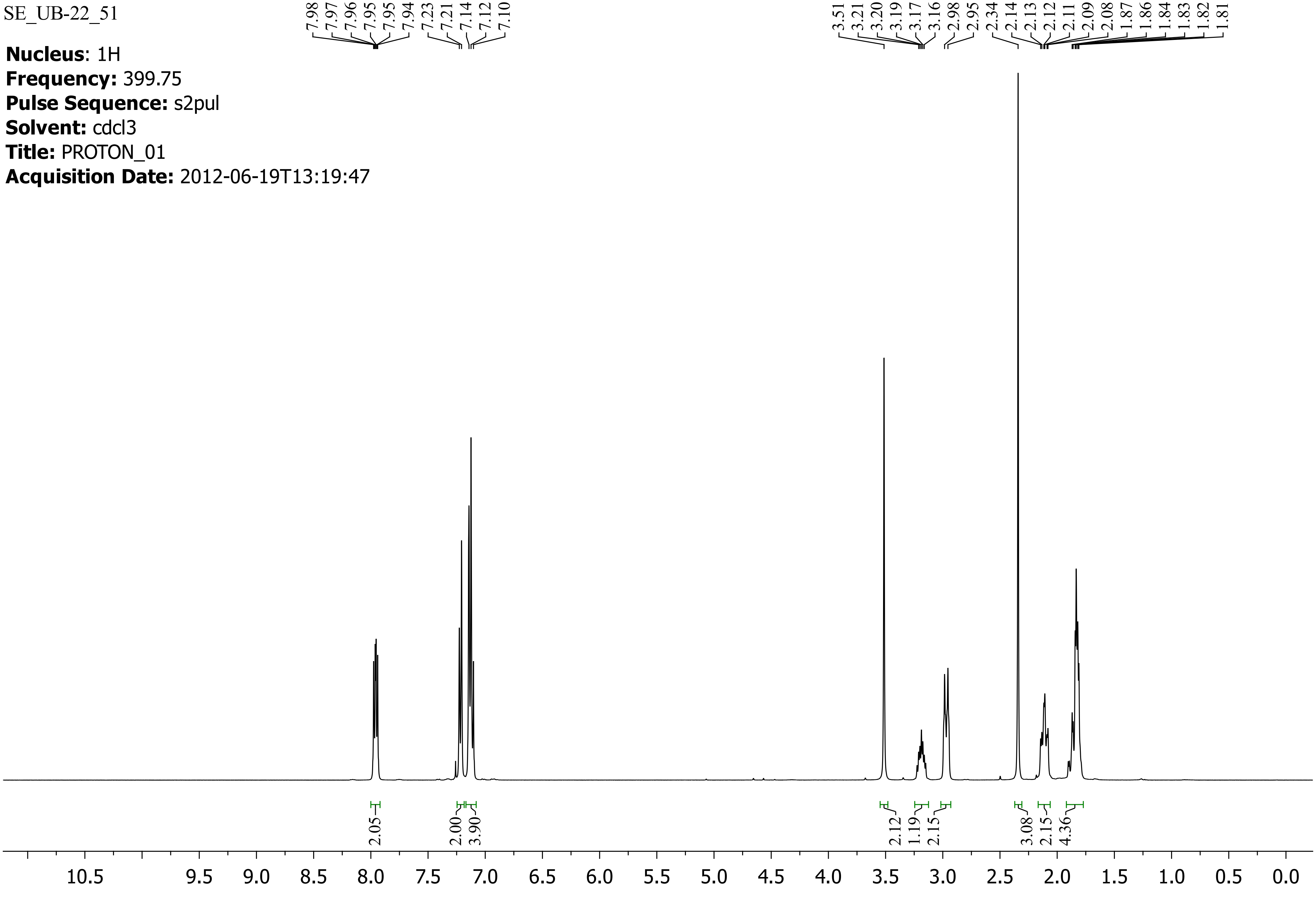

SE_UB-22_51

Nucleus: 1H
Frequency: 399.75
Pulse Sequence: s2pul
Solvent: cdcl3
Title: PROTON_01
Acquisition Date: 2012-06-19T13:19:47

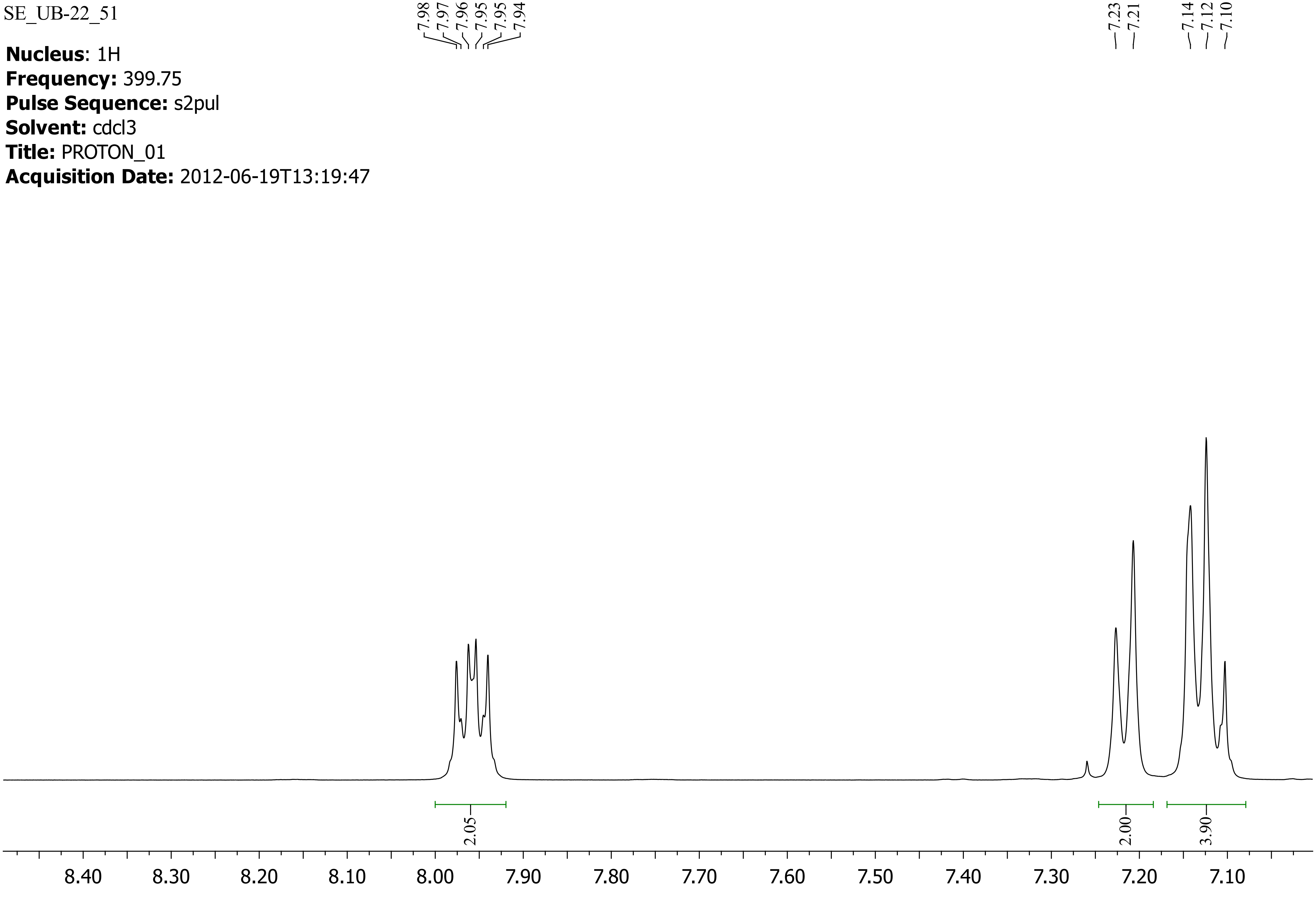

SE_UB-22_51

Nucleus: 1H
Frequency: 399.75
Pulse Sequence: s2pul
Solvent: cdcl3
Title: PROTON_01
Acquisition Date: 2012-06-19T13:19:47

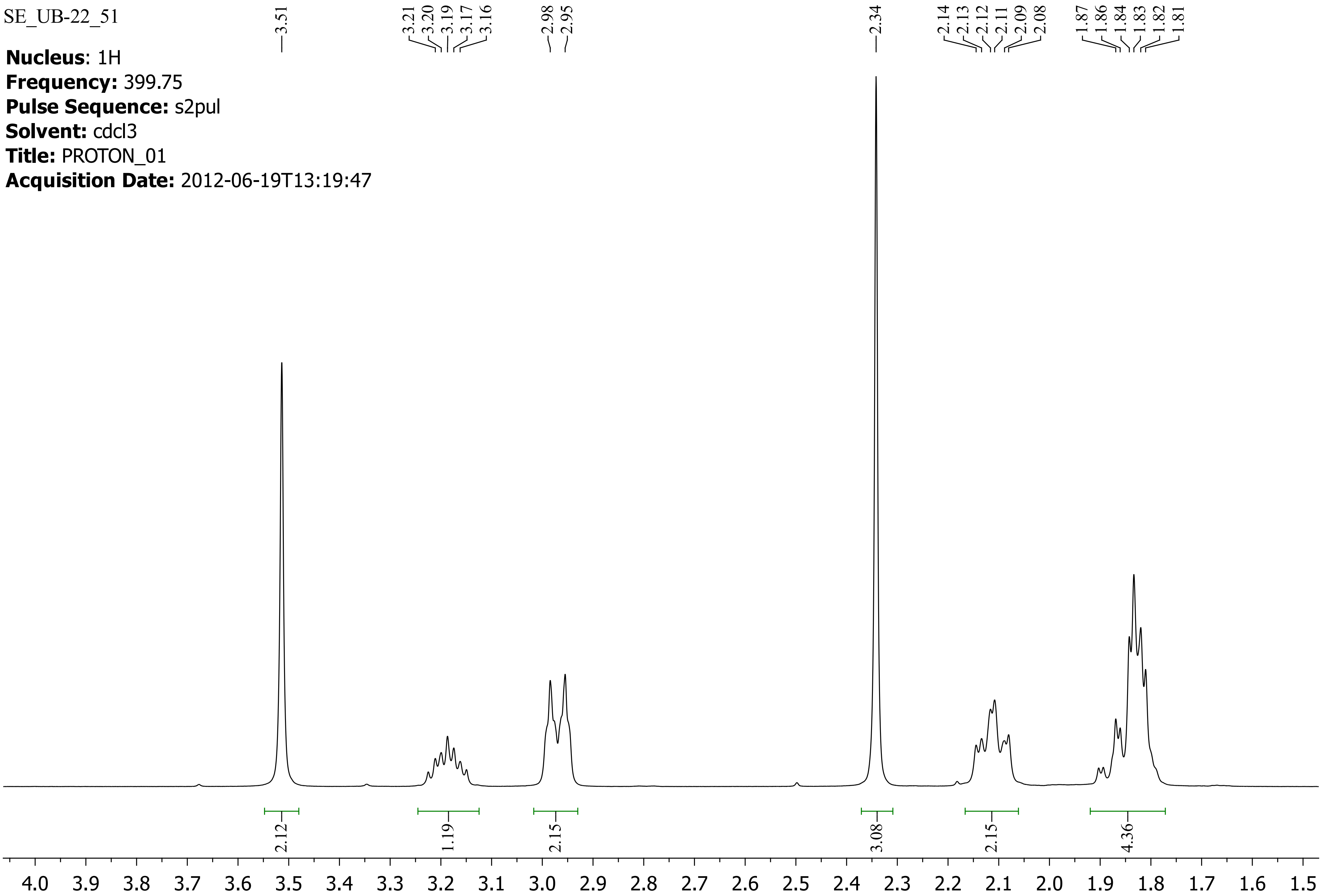

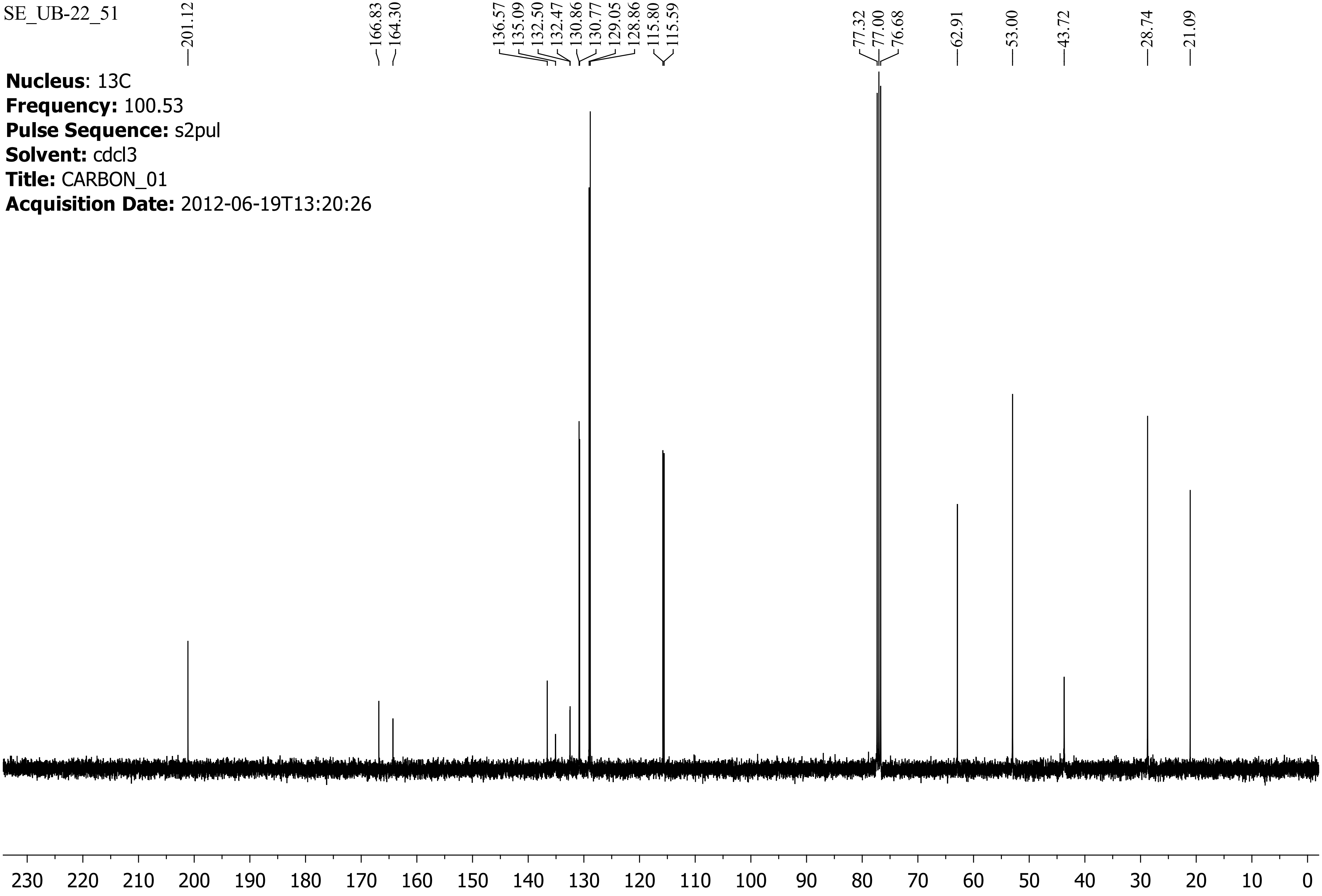

SE_UB-22_51
Nucleus: 13C
Frequency: 100.53
Pulse Sequence: s2pul
Solvent: cdcl3
Title: CARBON_01
Acquisition Date: 2012-06-19T13:20:26
201.12
166.83
164.30
136.57
135.09
132.50
132.47
130.86
130.77
129.05
128.86
115.80
115.59
77.32
77.00
76.68
62.91
53.00
43.72
28.74
21.09
230 220 210 200 190 180 170 160 150 140 130 120 110 100 90 80 70 60 50 40 30 20 10 0

SE_UB-22_51

Nucleus: 13C
Frequency: 100.53
Pulse Sequence: s2pul
Solvent: cdcl3
Title: CARBON_01
Acquisition Date: 2012-06-19T13:20:26

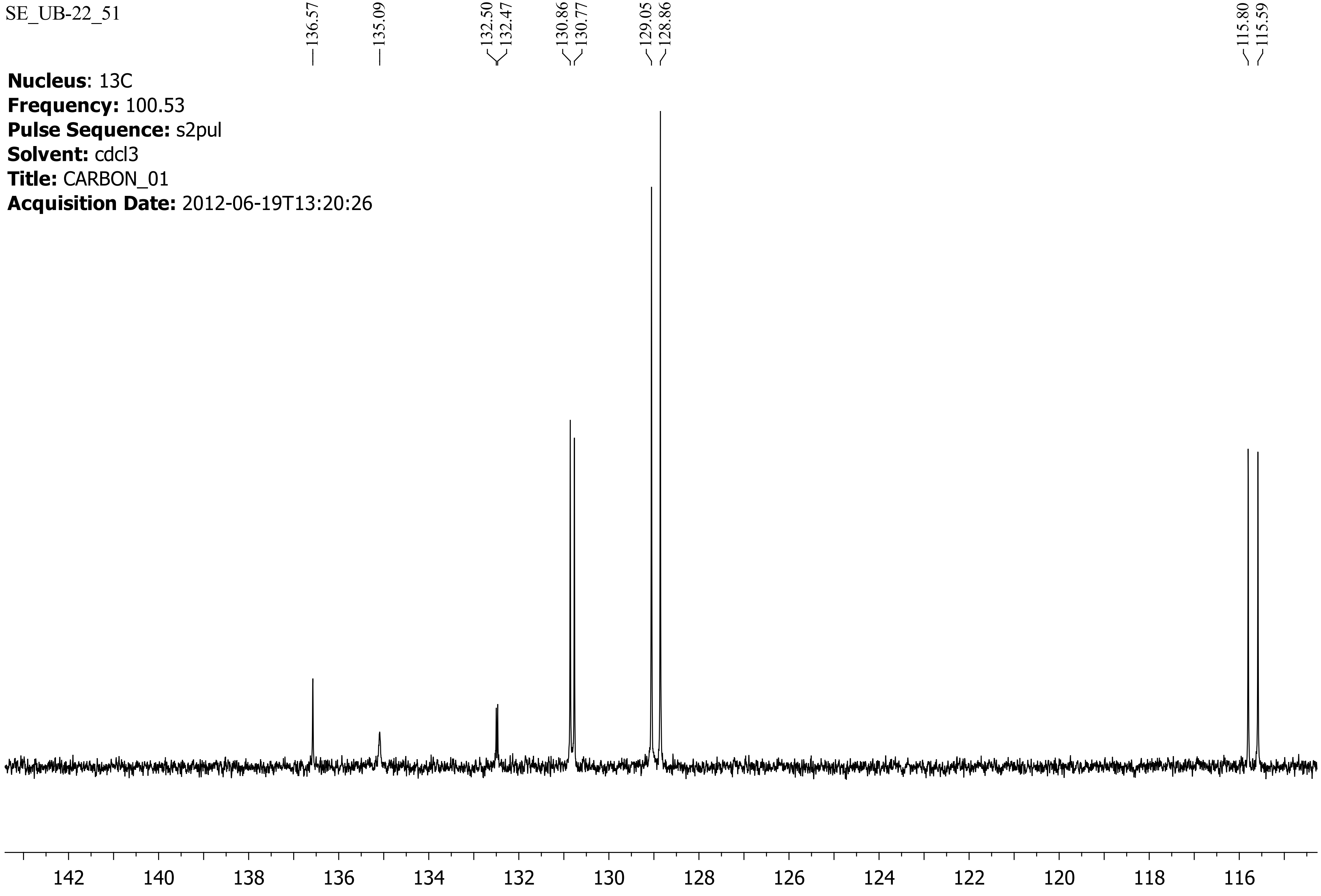

SE_UB-22_52

Nucleus: 1H
Frequency: 399.75
Pulse Sequence: s2pul
Solvent: cd3od
Title: PROTON_01
Acquisition Date: 2012-06-21T15:22:30

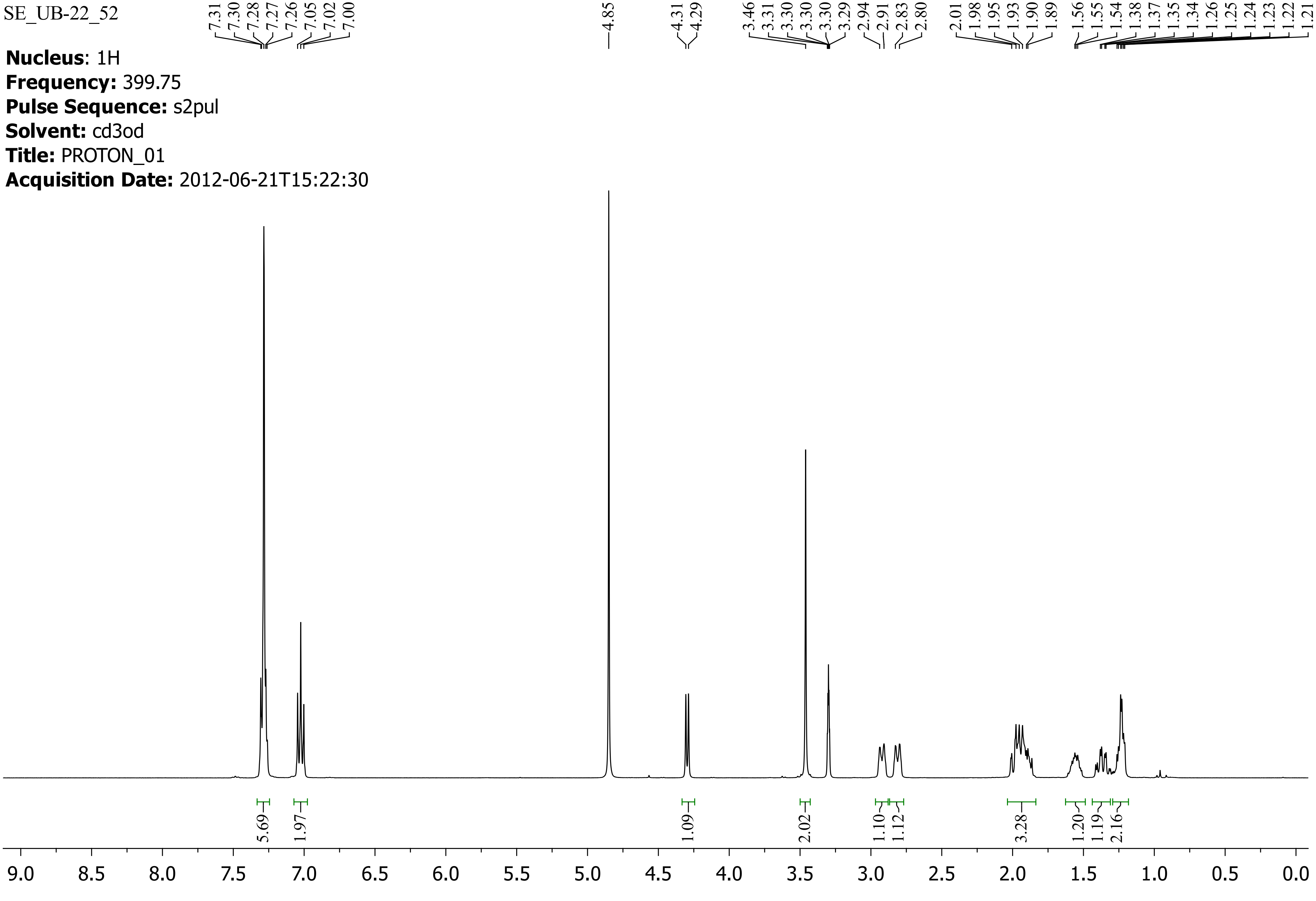

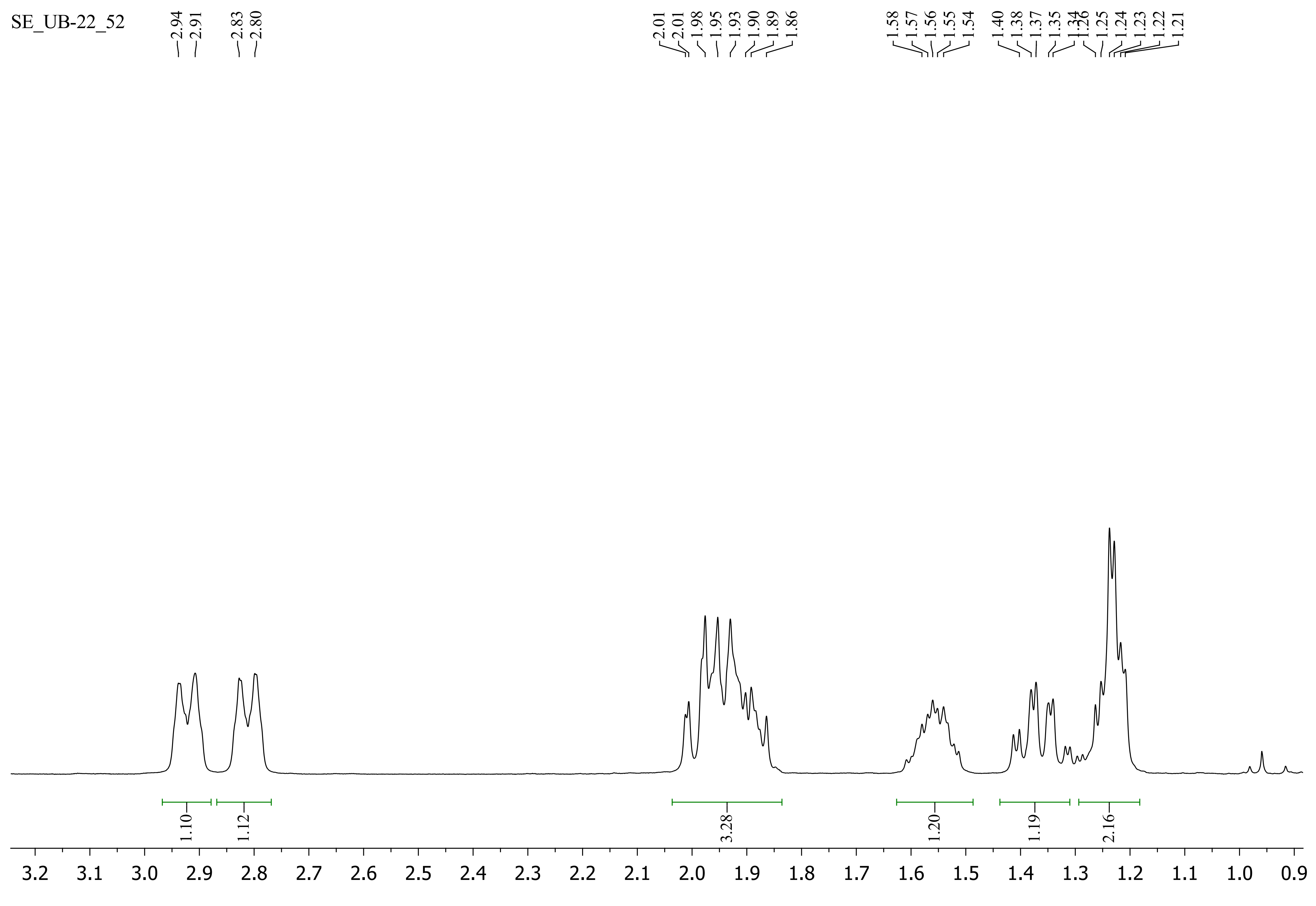
SE_UB-22_52
2.94
2.91
2.83
2.80
2.01
2.01
1.98
1.95
1.93
1.90
1.89
1.86
1.58
1.57
1.56
1.55
1.54
1.40
1.38
1.37
1.35
1.34
1.26
1.25
1.24
1.23
1.22
1.21
1.10
1.12
3.28
1.20
1.19
2.16
3.2
3.1
3.0
2.9
2.8
2.7
2.6
2.5
2.4
2.3
2.2
2.1
2.0
1.9
1.8
1.7
1.6
1.5
1.4
1.3
1.2
1.1
1.0
0.9

SE_UB-22_52

Nucleus: 13C
Frequency: 100.53
Pulse Sequence: s2pul
Solvent: cd3od
Title: CARBON_01
Acquisition Date: 2012-06-21T15:26:49

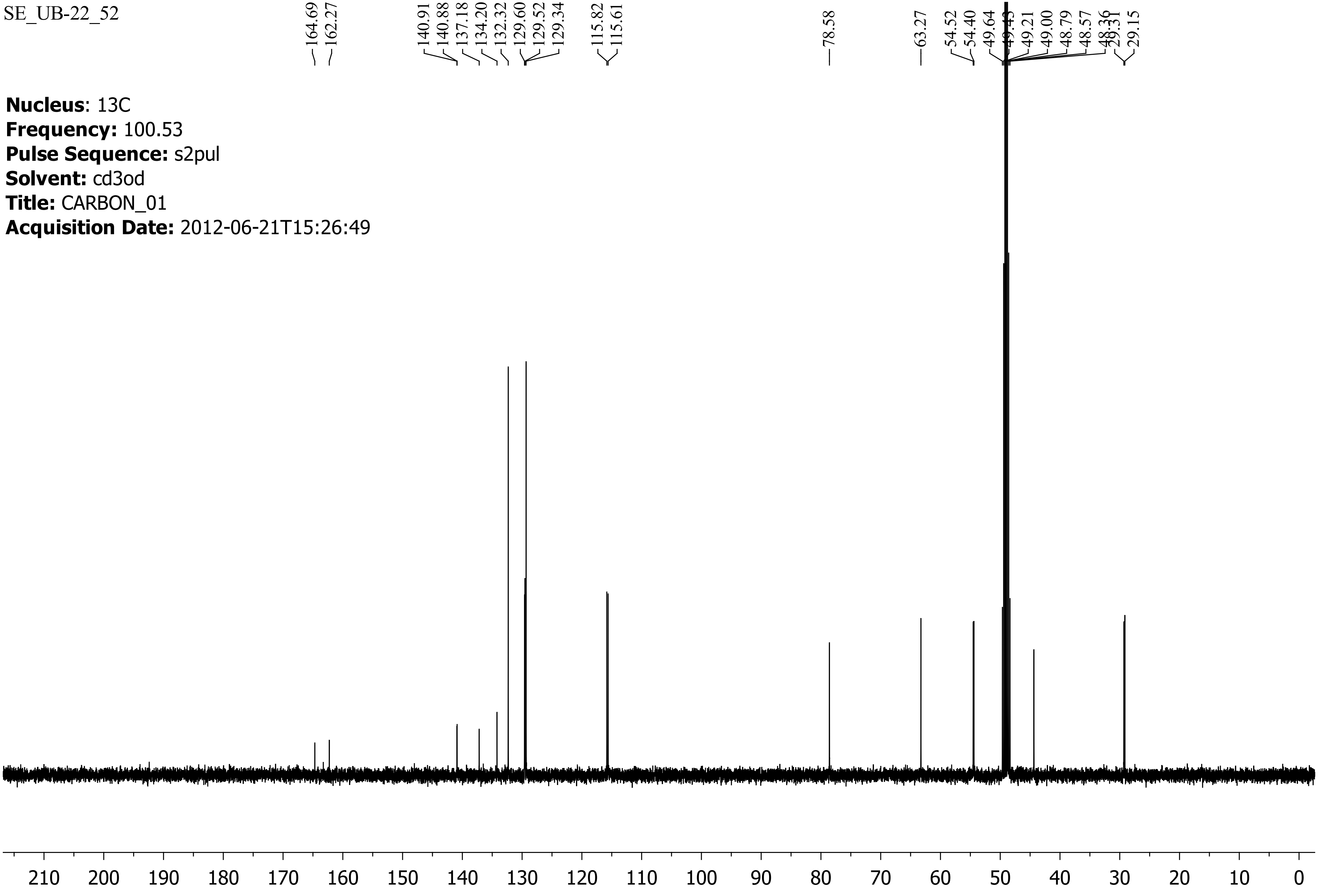

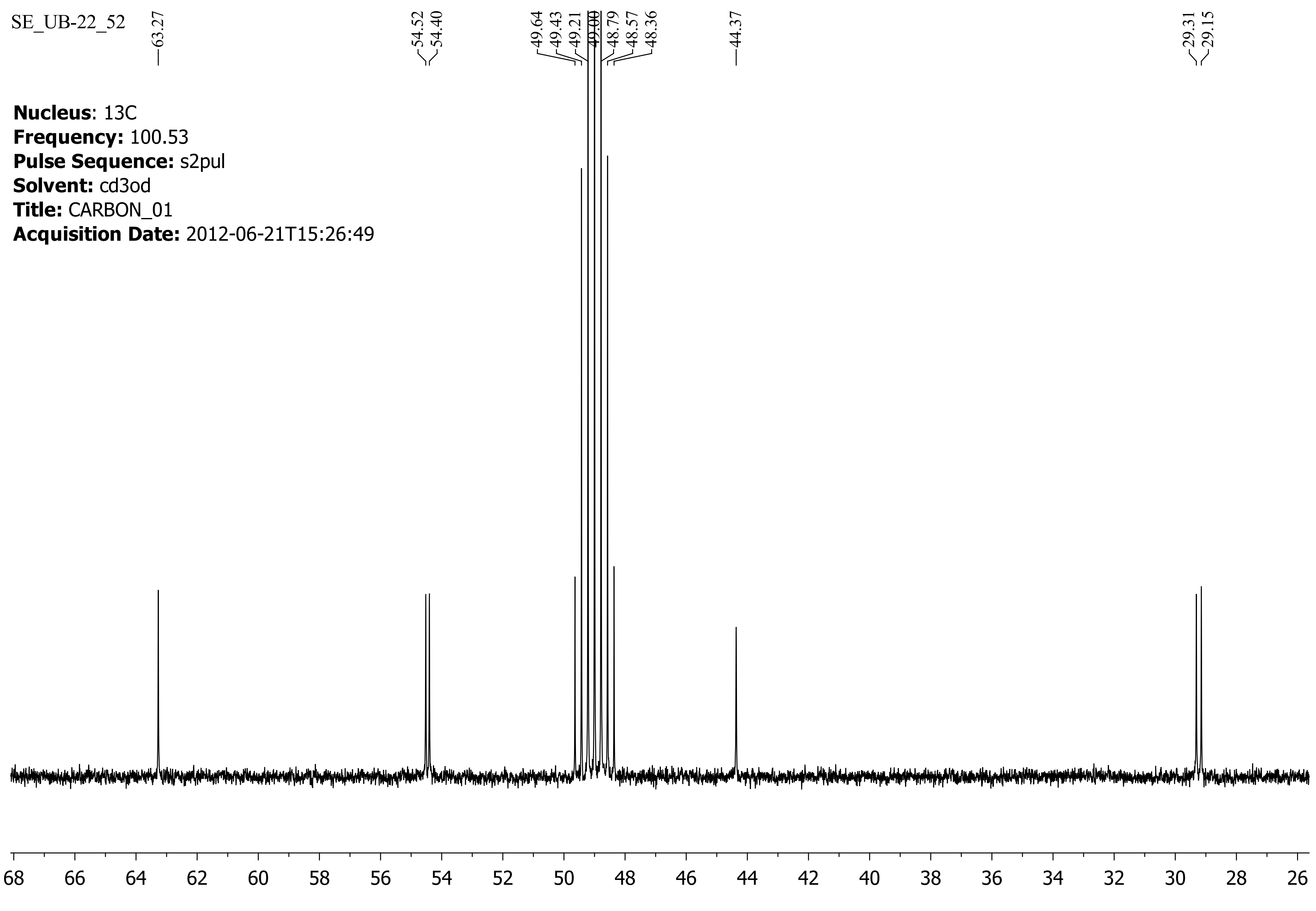

SE_UB-22_52
63.27
54.52
54.40
49.64
49.43
49.21
49.00
48.79
48.57
48.36
44.37
29.31
29.15
Nucleus: 13C
Frequency: 100.53
Pulse Sequence: s2pul
Solvent: cd3od
Title: CARBON_01
Acquisition Date: 2012-06-21T15:26:49
68
66
64
62
60
58
56
54
52
50
48
46
44
42
40
38
36
34
32
30
28
26

SE_UB-22_53

Nucleus: 1H
Frequency: 399.75
Pulse Sequence: s2pul
Solvent: cd3od
Title: PROTON_01
Acquisition Date: 2012-06-21T15:41:43

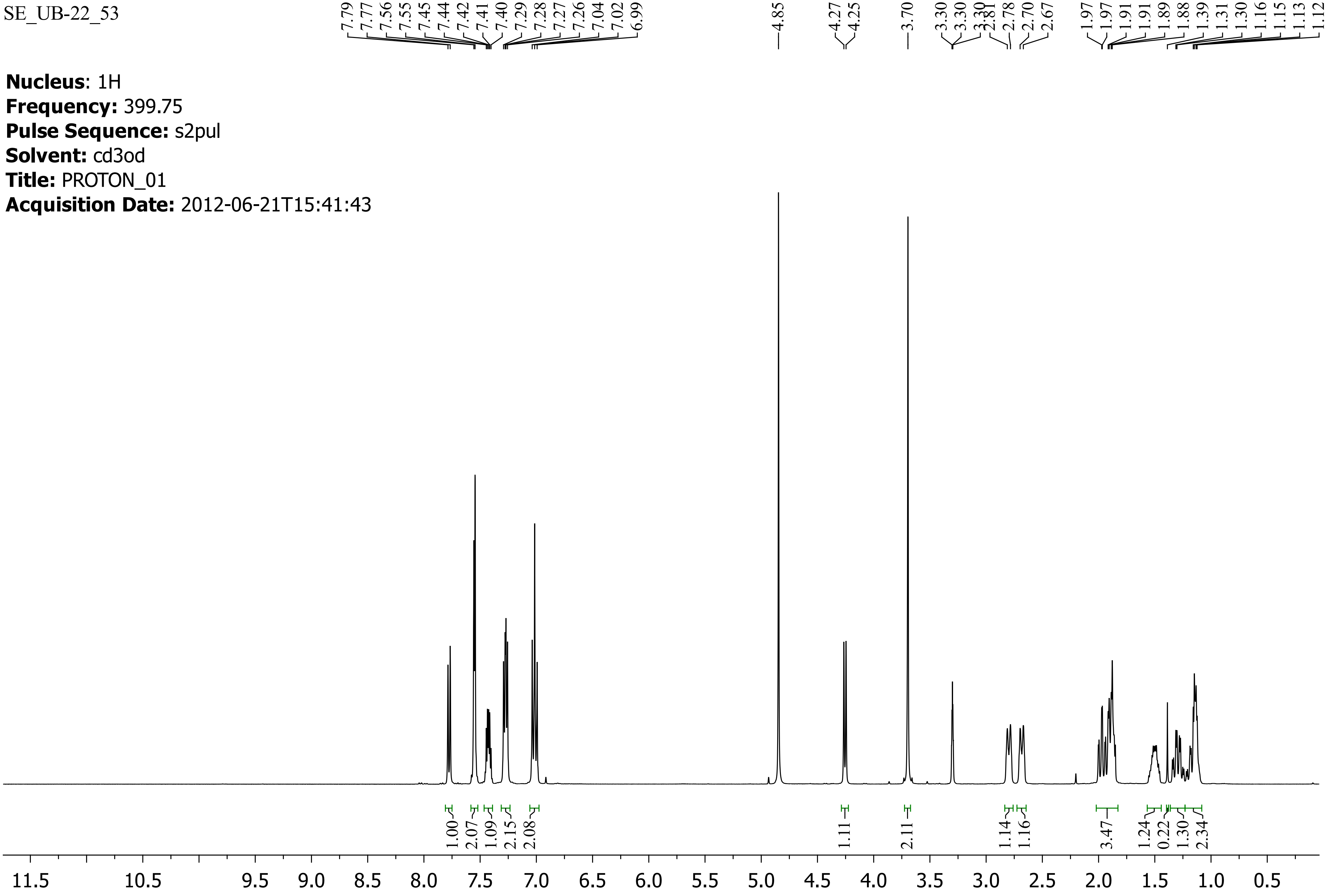

SE_UB-22_53

Nucleus: 1H
Frequency: 399.75
Pulse Sequence: s2pul
Solvent: cd3od
Title: PROTON_01
Acquisition Date: 2012-06-21T15:41:43

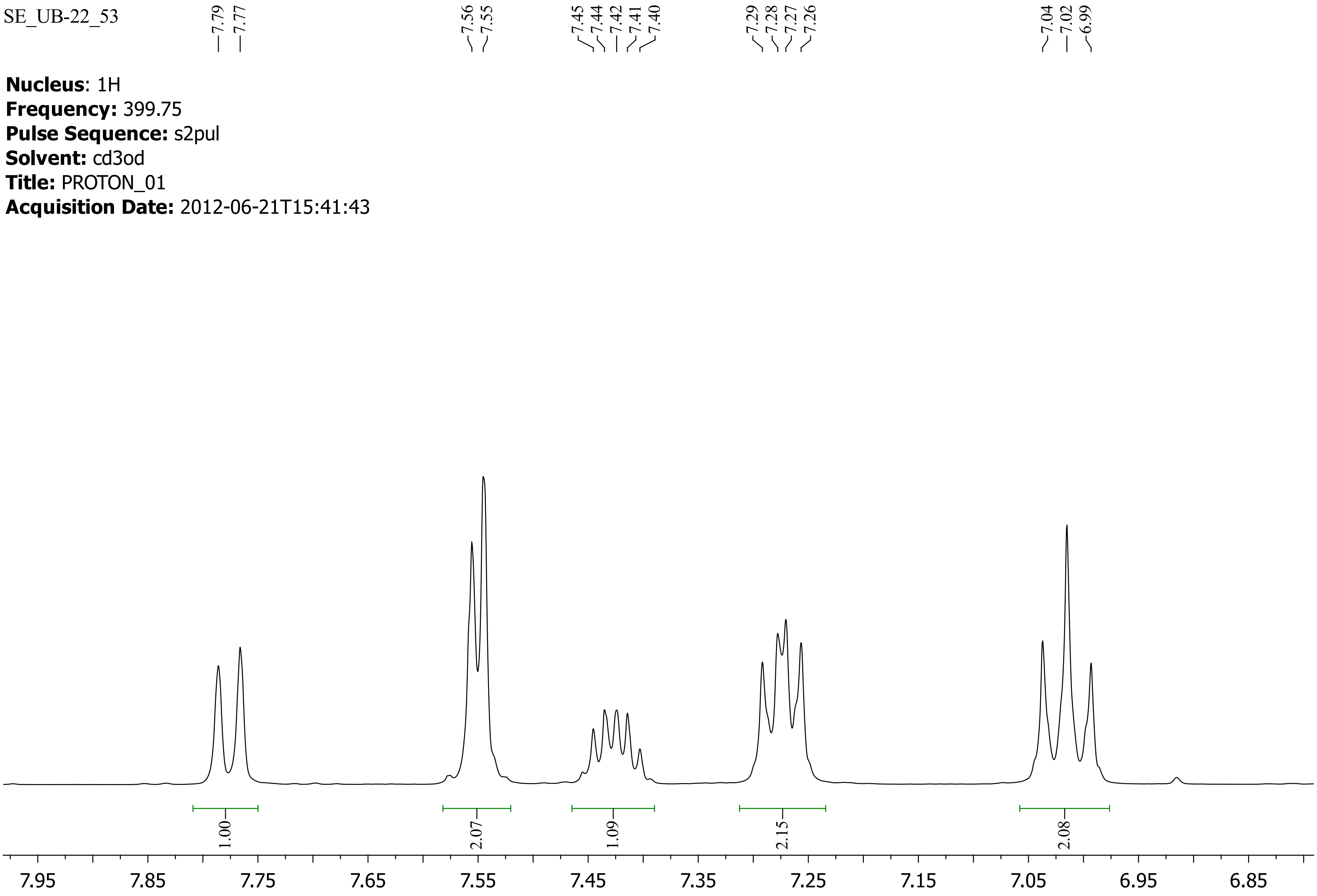

SE_UB-22_53

Nucleus: 1H
Frequency: 399.75
Pulse Sequence: s2pul
Solvent: cd3od
Title: PROTON_01
Acquisition Date: 2012-06-21T15:41:43

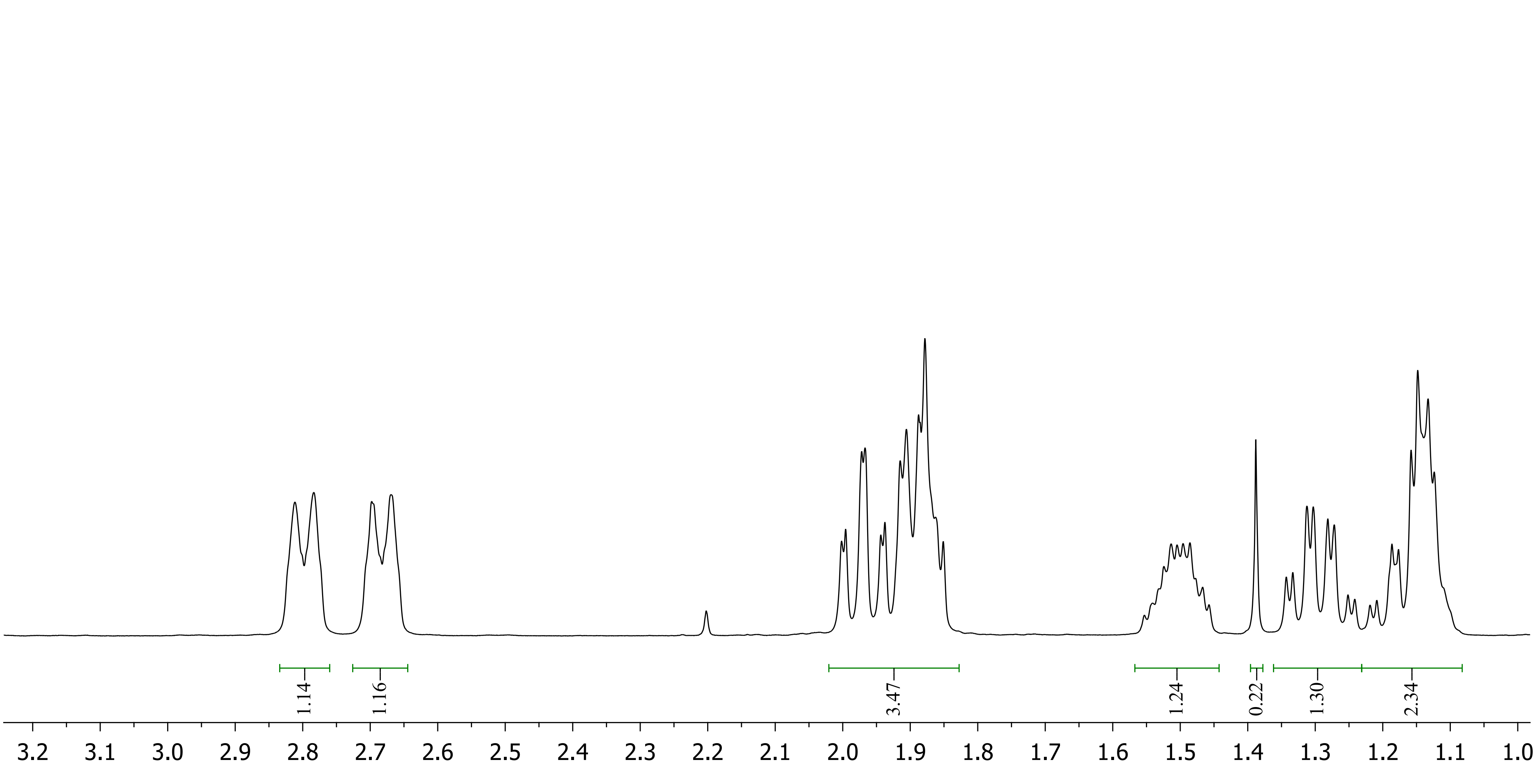

SE_UB-22_53

Nucleus: 13C
Frequency: 100.53
Pulse Sequence: s2pul
Solvent: cd3od
Title: CARBON_01
Acquisition Date: 2012-06-21T15:42:22

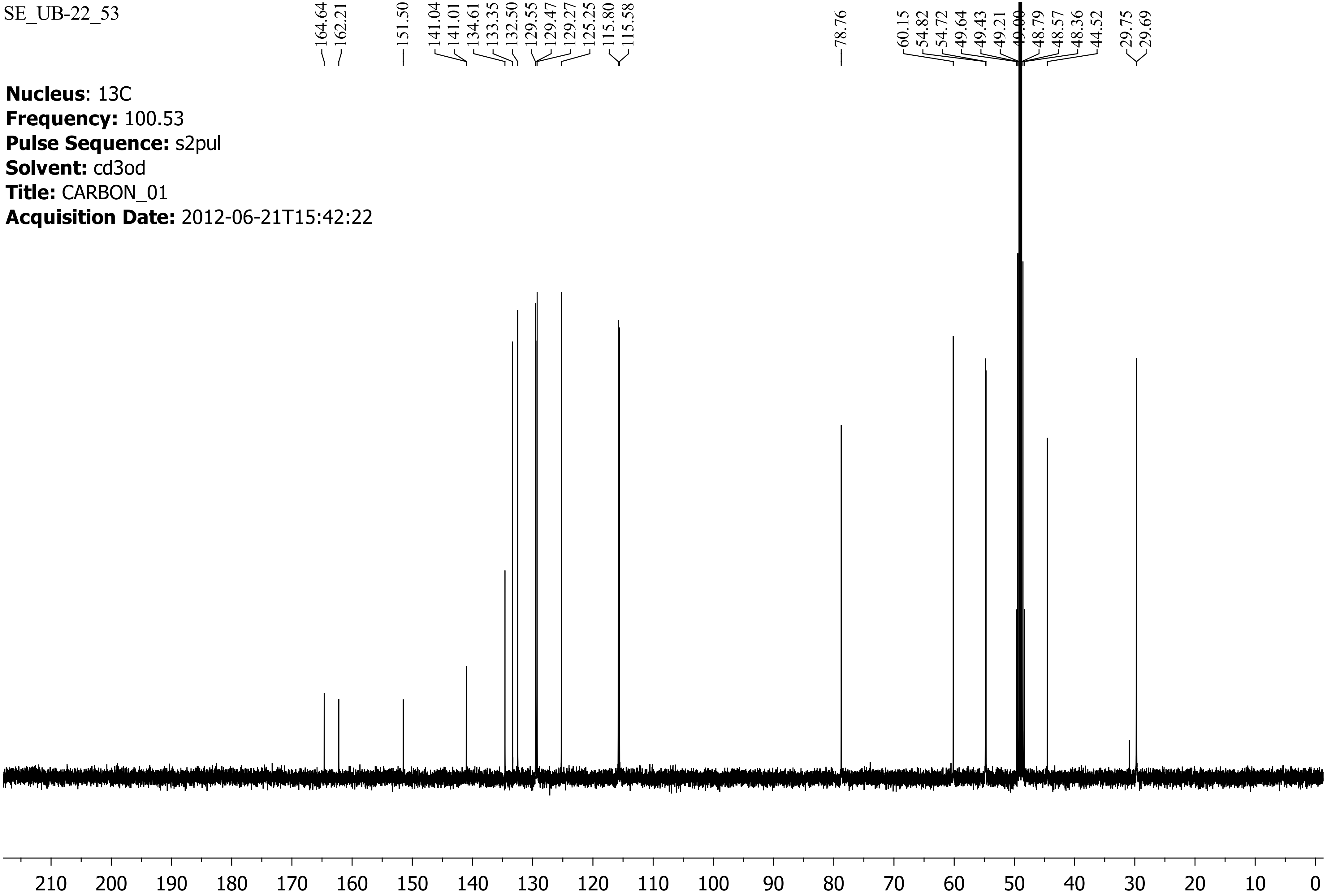

SE_UB-22_53

Nucleus: 13C
Frequency: 100.53
Pulse Sequence: s2pul
Solvent: cd3od
Title: CARBON_01
Acquisition Date: 2012-06-21T15:42:22

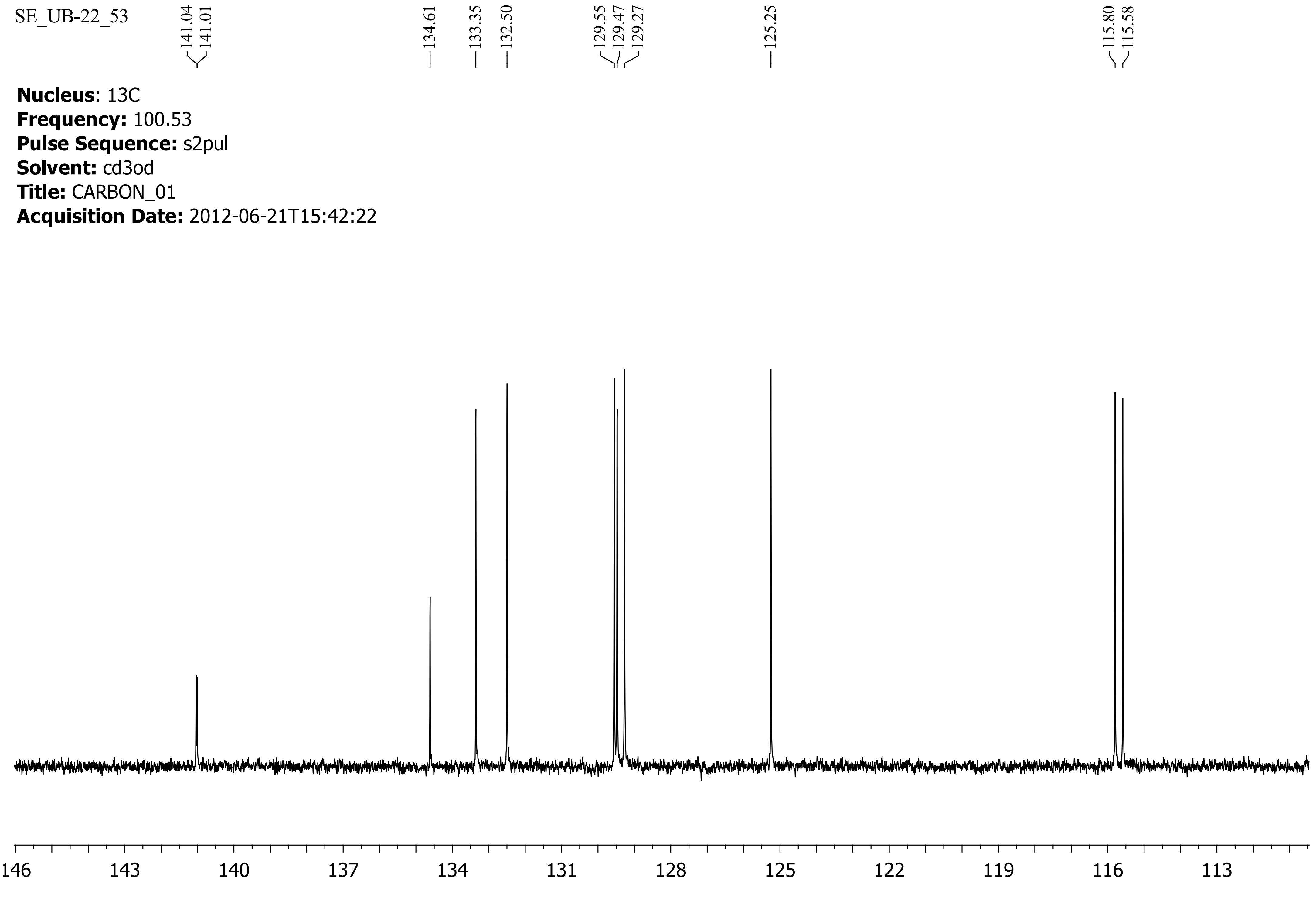

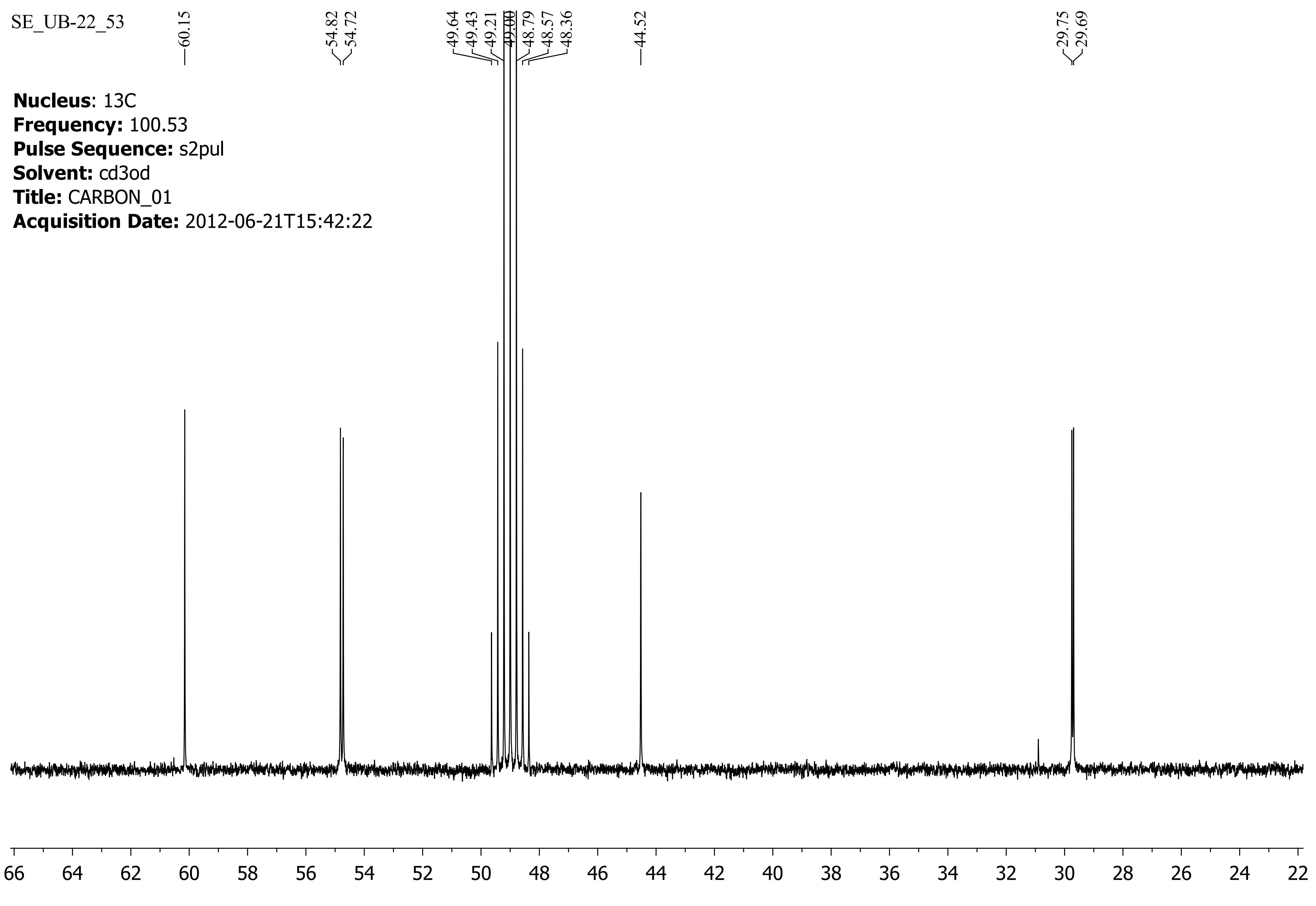
SE_UB-22_53
60.15
54.82
54.72
49.64
49.43
49.21
49.00
48.79
48.57
48.36
44.52
29.75
29.69
Nucleus: 13C
Frequency: 100.53
Pulse Sequence: s2pul
Solvent: cd3od
Title: CARBON_01
Acquisition Date: 2012-06-21T15:42:22
66 64 62 60 58 56 54 52 50 48 46 44 42 40 38 36 34 32 30 28 26 24 22

SE_UB-22_54

Nucleus: 1H
Frequency: 399.75
Pulse Sequence: s2pul
Solvent: cd3od
Title: PROTON_01
Acquisition Date: 2012-06-21T15:57:18

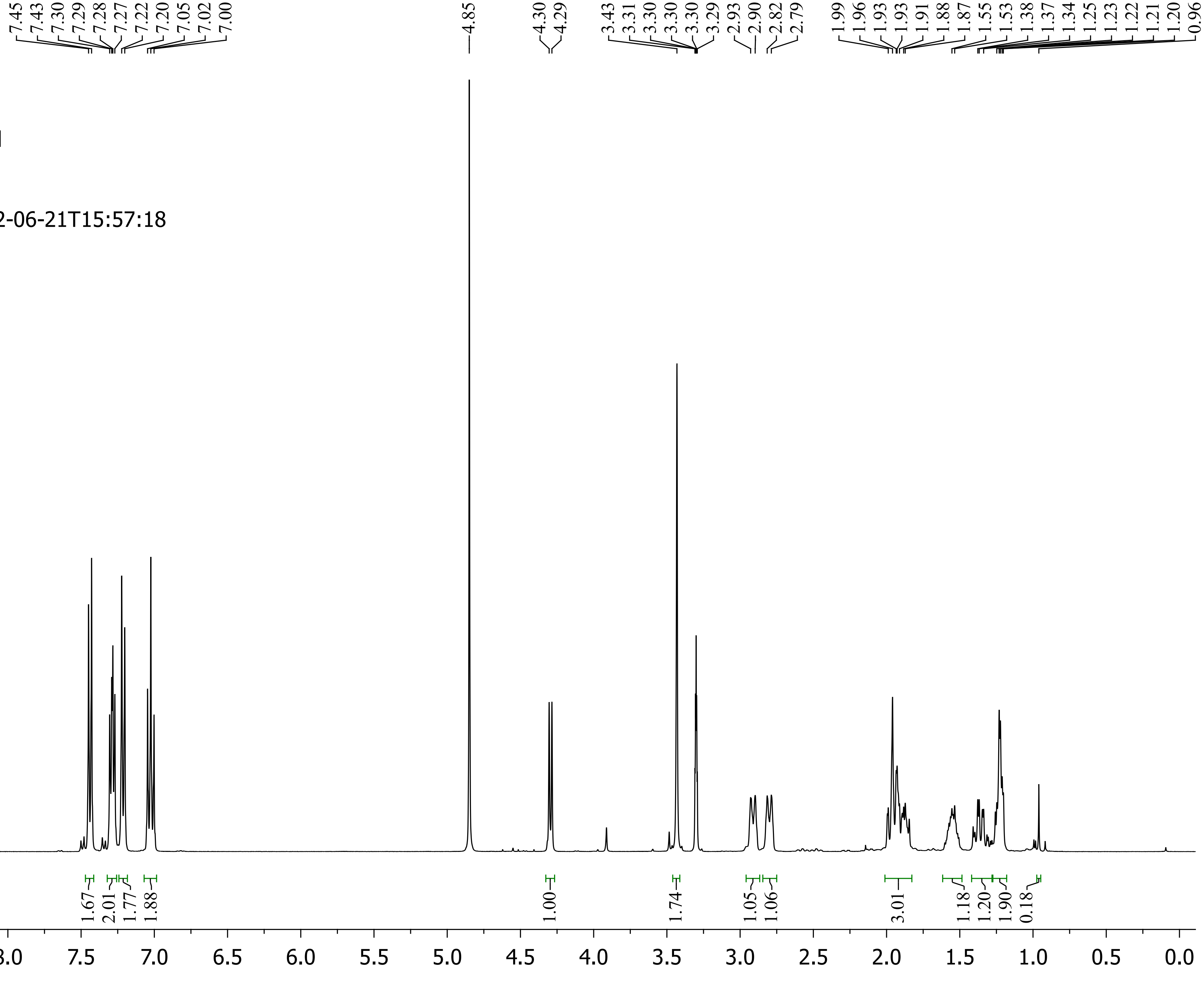

SE_UB-22_54

Nucleus: 1H
Frequency: 399.75
Pulse Sequence: s2pul
Solvent: cd3od
Title: PROTON_01
Acquisition Date: 2012-06-21T15:57:18

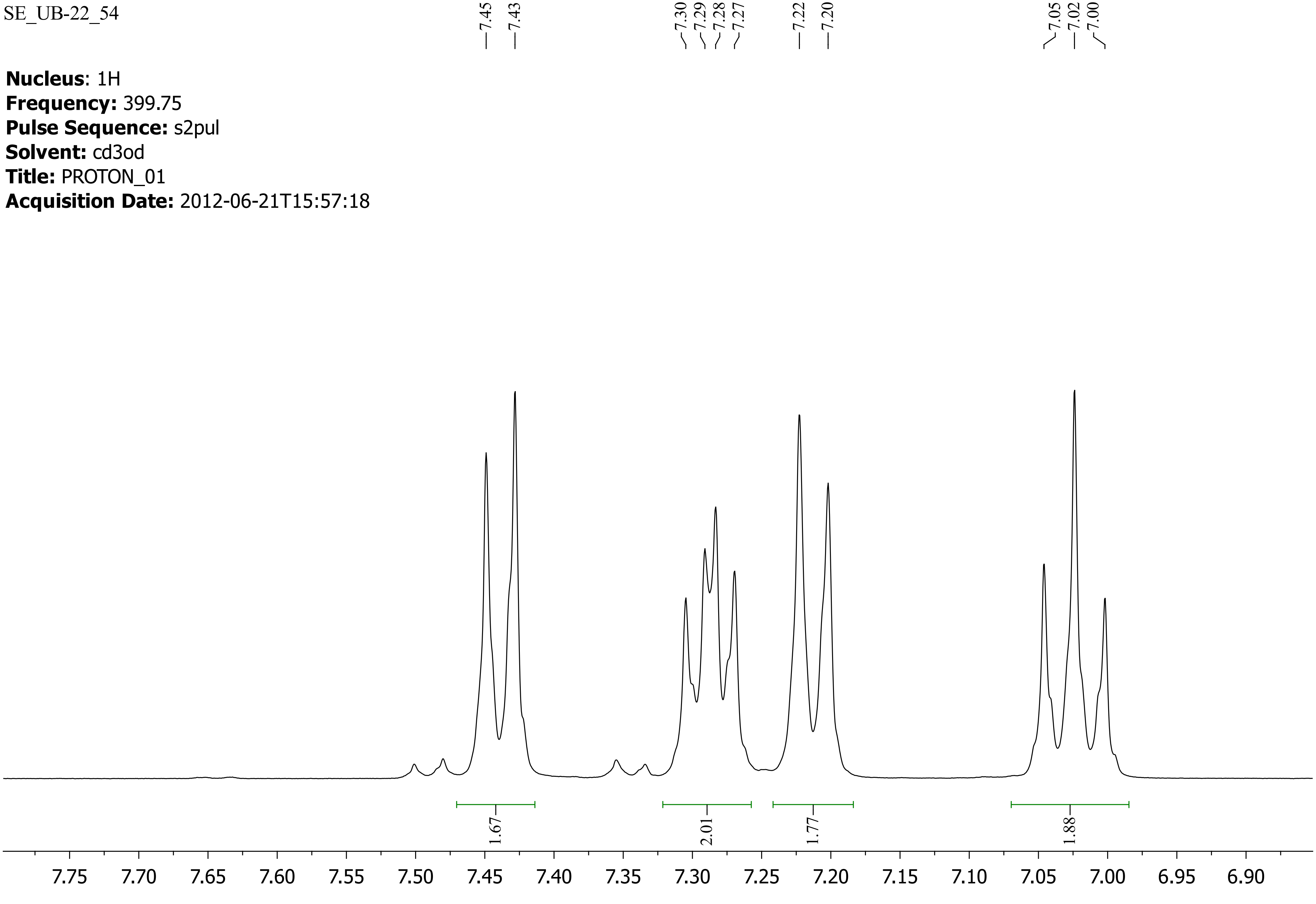

SE_UB-22_54

Nucleus: 1H
Frequency: 399.75
Pulse Sequence: s2pul
Solvent: cd3od
Title: PROTON_01
Acquisition Date: 2012-06-21T15:57:18

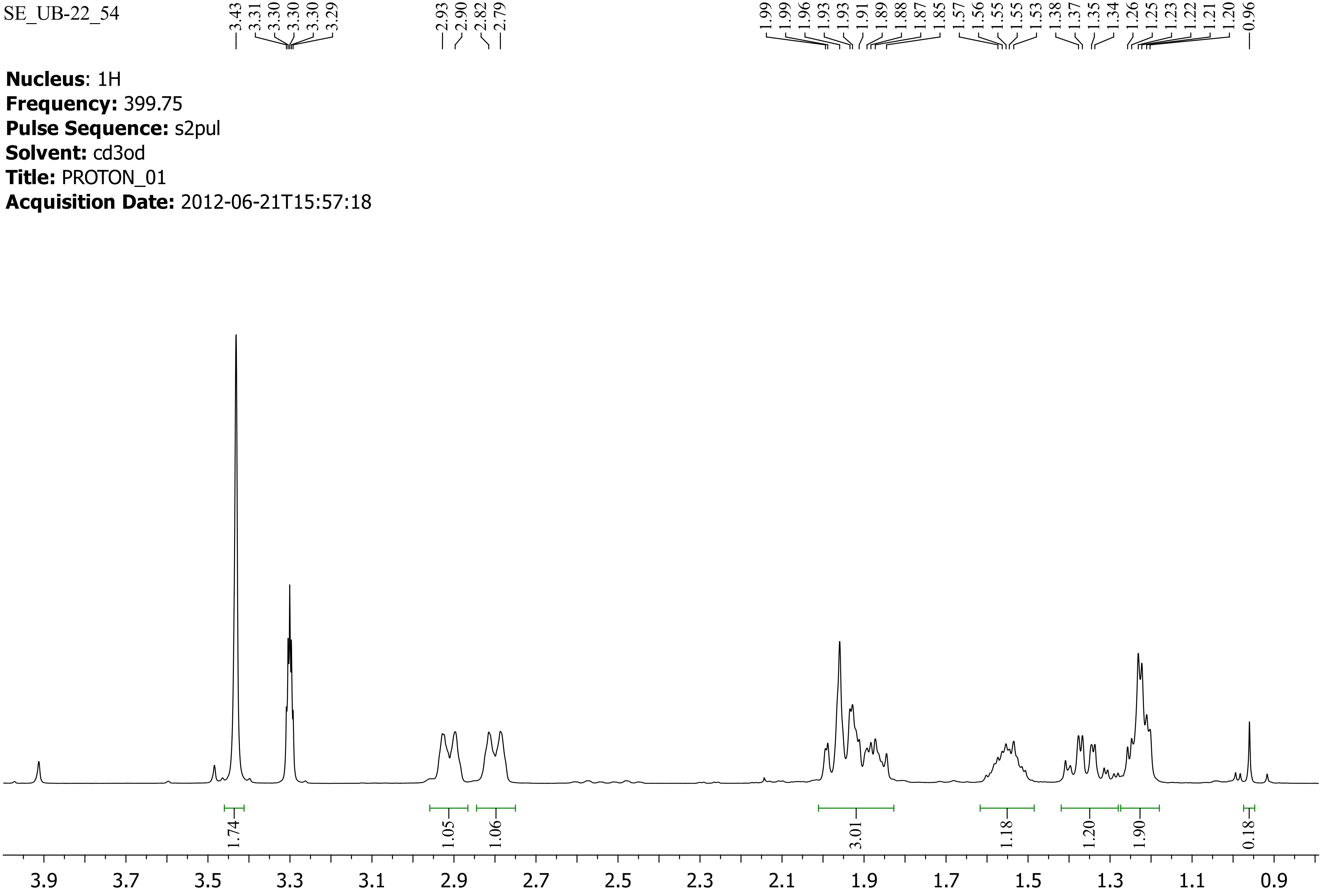

SE_UB-22_54

Nucleus: 13C
Frequency: 100.53
Pulse Sequence: s2pul
Solvent: cd3od
Title: CARBON_01
Acquisition Date: 2012-06-21T15:57:57

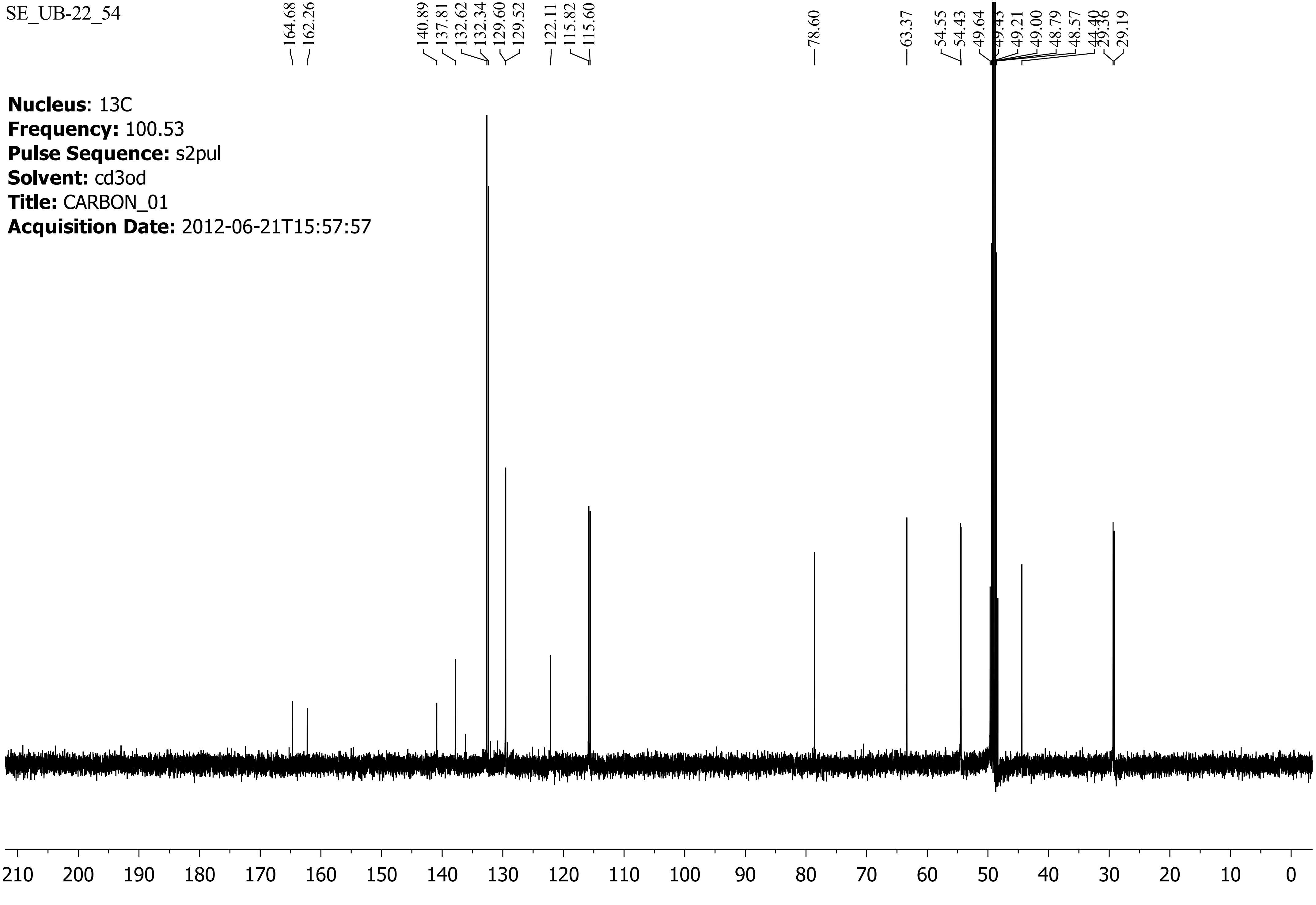

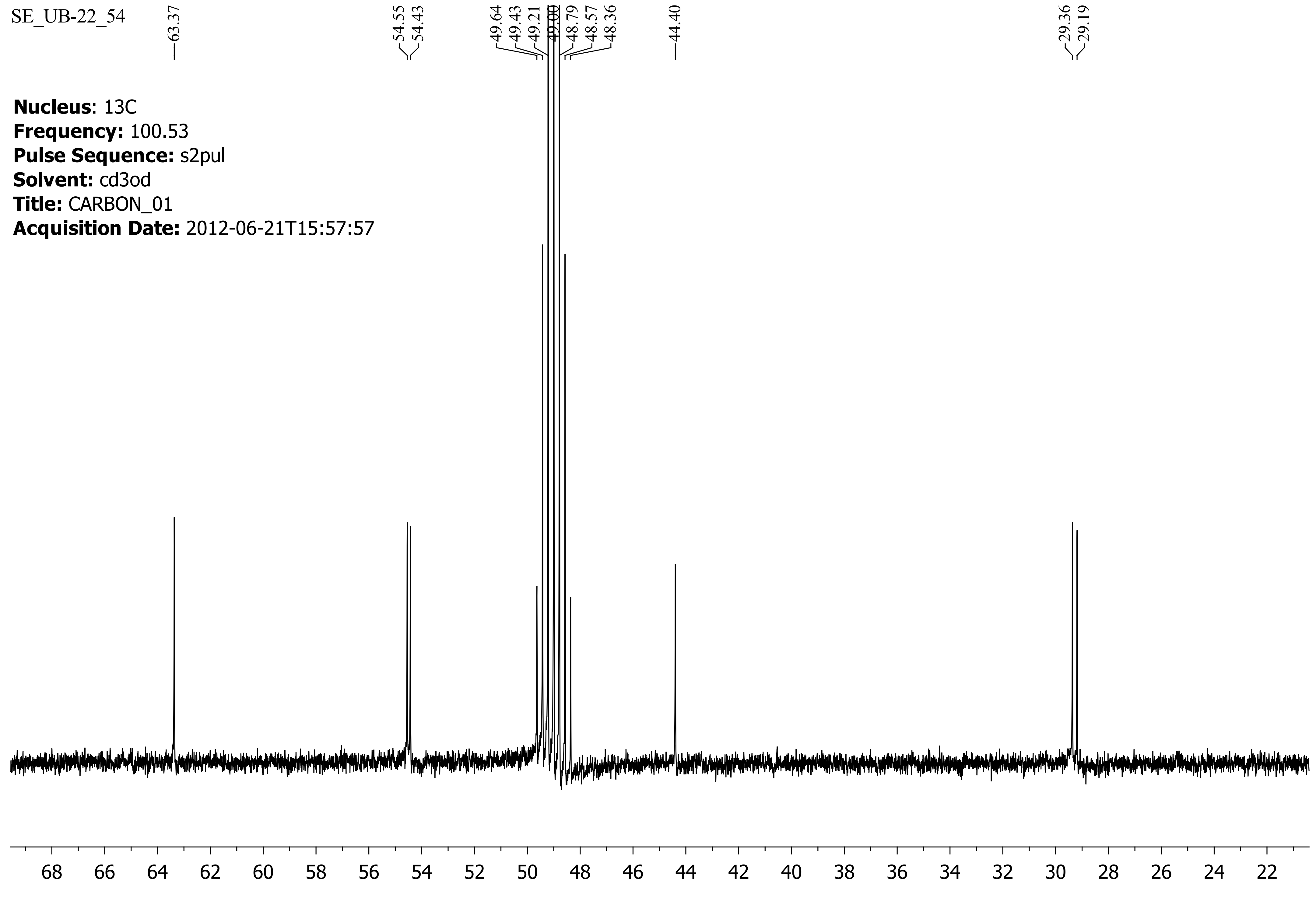
SE_UB-22_54
63.37
54.55
54.43
49.64
49.43
49.21
49.00
48.79
48.57
48.36
44.40
29.36
29.19
Nucleus: 13C
Frequency: 100.53
Pulse Sequence: s2pul
Solvent: cd3od
Title: CARBON_01
Acquisition Date: 2012-06-21T15:57:57
68
66
64
62
60
58
56
54
52
50
48
46
44
42
40
38
36
34
32
30
28
26
24
22

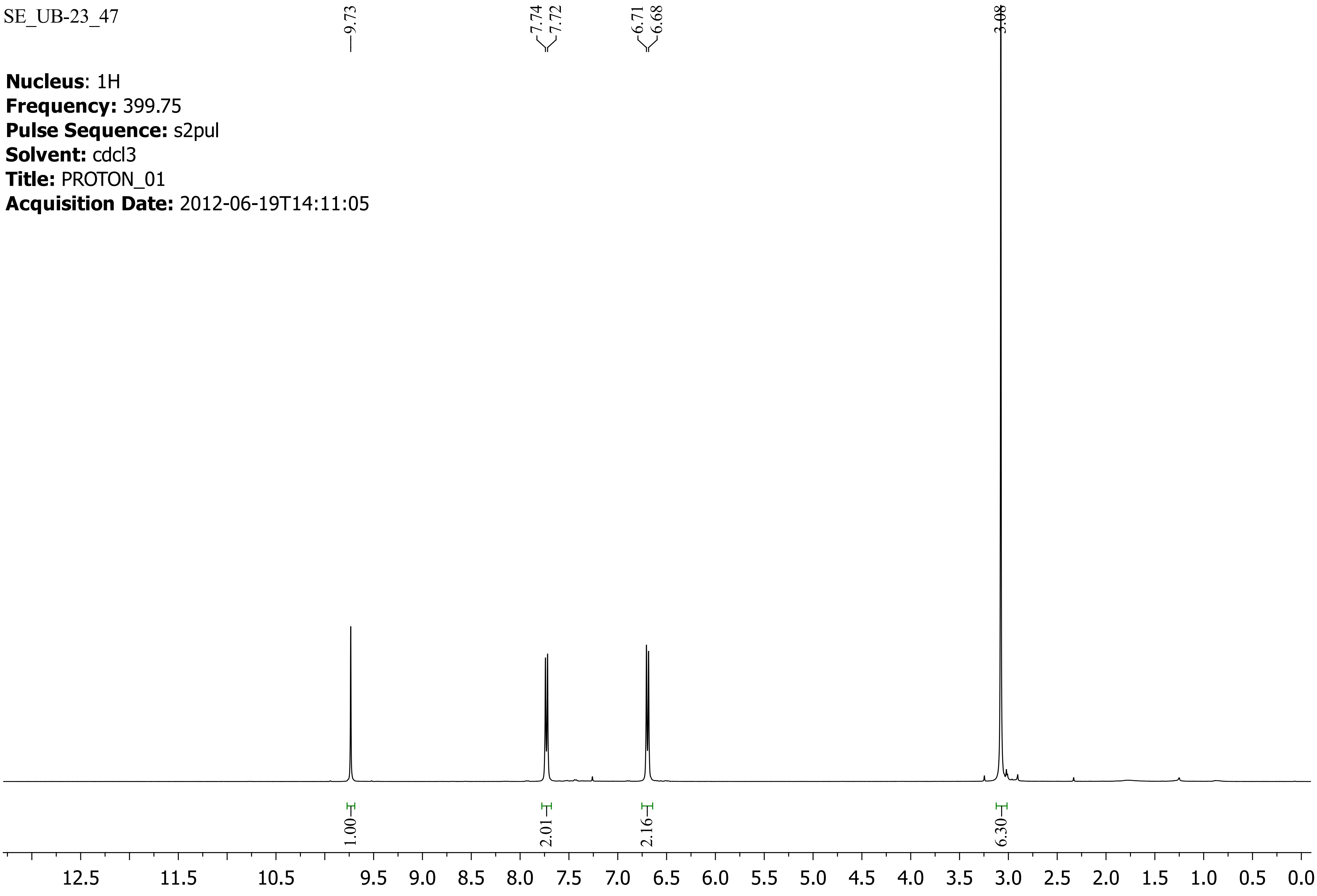
SE_UB-23_47
Nucleus: 1H
Frequency: 399.75
Pulse Sequence: s2pul
Solvent: cdcl3
Title: PROTON_01
Acquisition Date: 2012-06-19T14:11:05
9.73
7.74
7.72
6.71
6.68
3.08
1.00
2.01
2.16
6.30
12.5 11.5 10.5 9.5 9.0 8.5 8.0 7.5 7.0 6.5 6.0 5.5 5.0 4.5 4.0 3.5 3.0 2.5 2.0 1.5 1.0 0.5 0.0

SE_UB-23_47_carbon

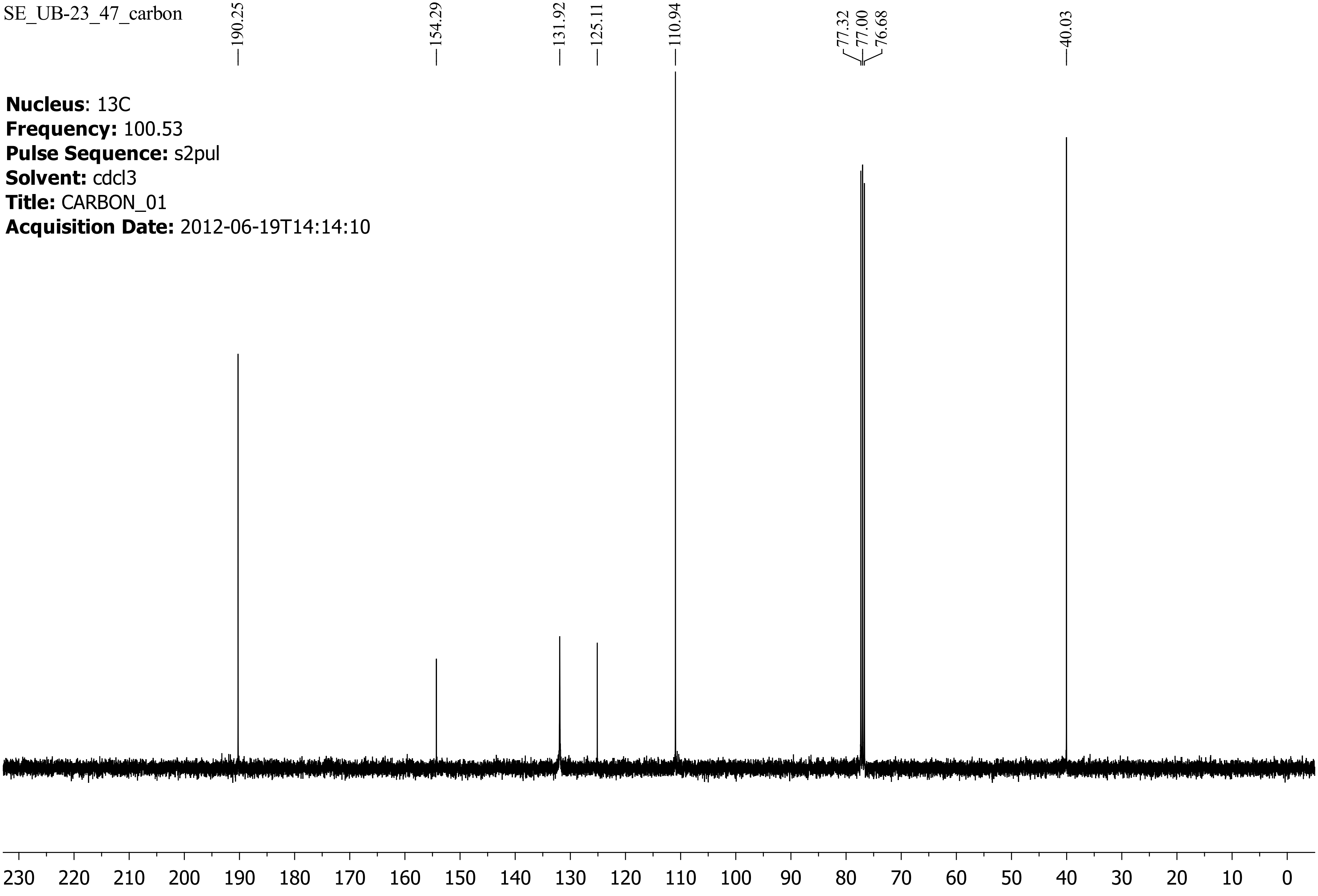

Nucleus: 13C
Frequency: 100.53
Pulse Sequence: s2pul
Solvent: cdcl3
Title: CARBON_01
Acquisition Date: 2012-06-19T14:14:10
