# Supplementary material for: 4-aroylpiperidines and 4-(α-hydroxyphenyl)piperidines as selective sigma-1 receptor ligands: synthesis, preliminary pharmacological evaluation and computational studies
Source: Chem Cent J. 2016 Aug 23;10(1):53. doi: 10.1186/s13065-016-0200-1 (PMC4994268; doi:10.1186/s13065-016-0200-1)

SE_UB-22_55

Nucleus: 1H
Frequency: 399.75
Pulse Sequence: s2pul
Solvent: cd3od
Title: PROTON_01
Acquisition Date: 2012-06-21T16:12:50

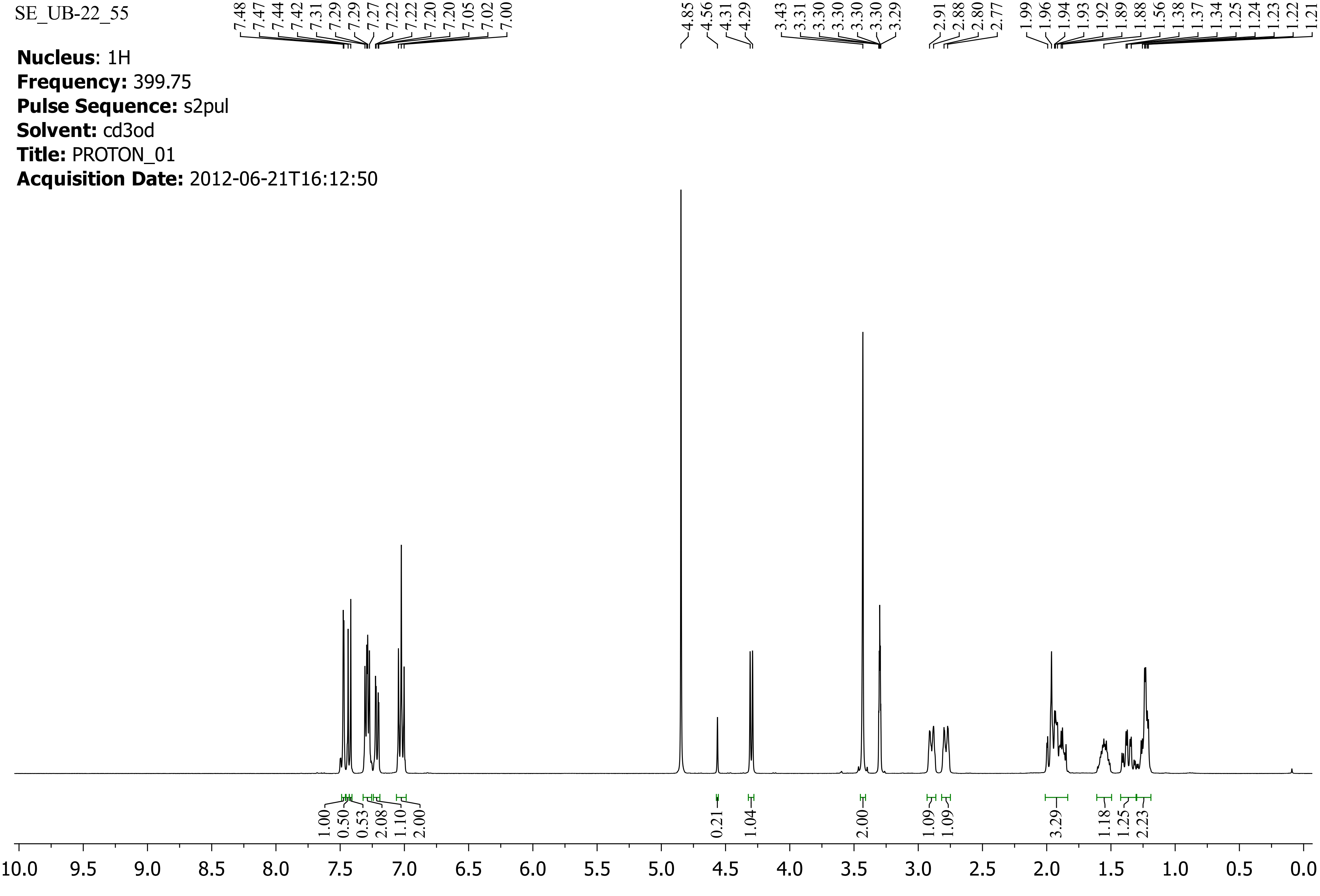

SE_UB-22_55

Nucleus: 1H
Frequency: 399.75
Pulse Sequence: s2pul
Solvent: cd3od
Title: PROTON_01
Acquisition Date: 2012-06-21T16:12:50

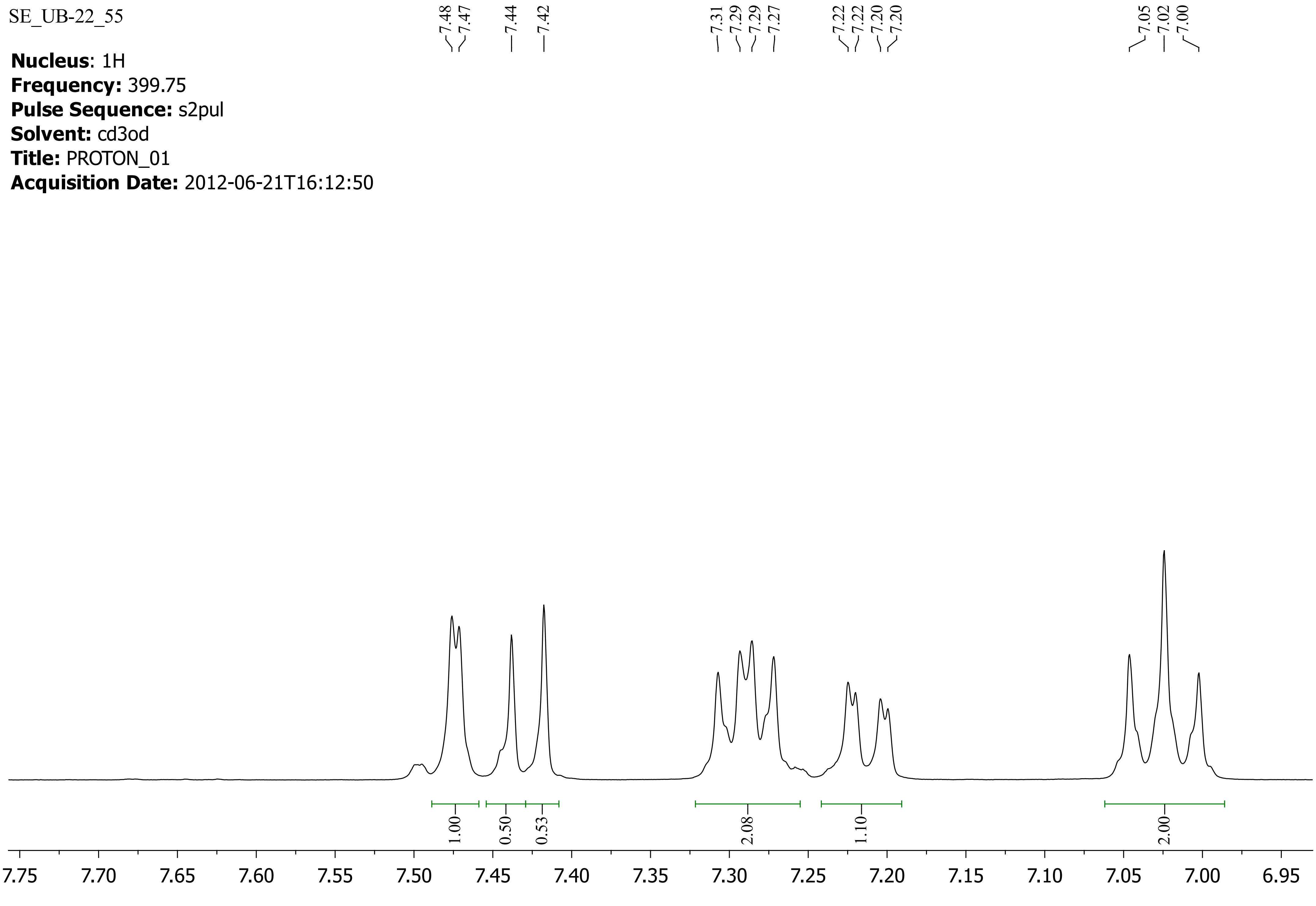

SE_UB-22_55

Nucleus: 1H
Frequency: 399.75
Pulse Sequence: s2pul
Solvent: cd3od
Title: PROTON_01
Acquisition Date: 2012-06-21T16:12:50

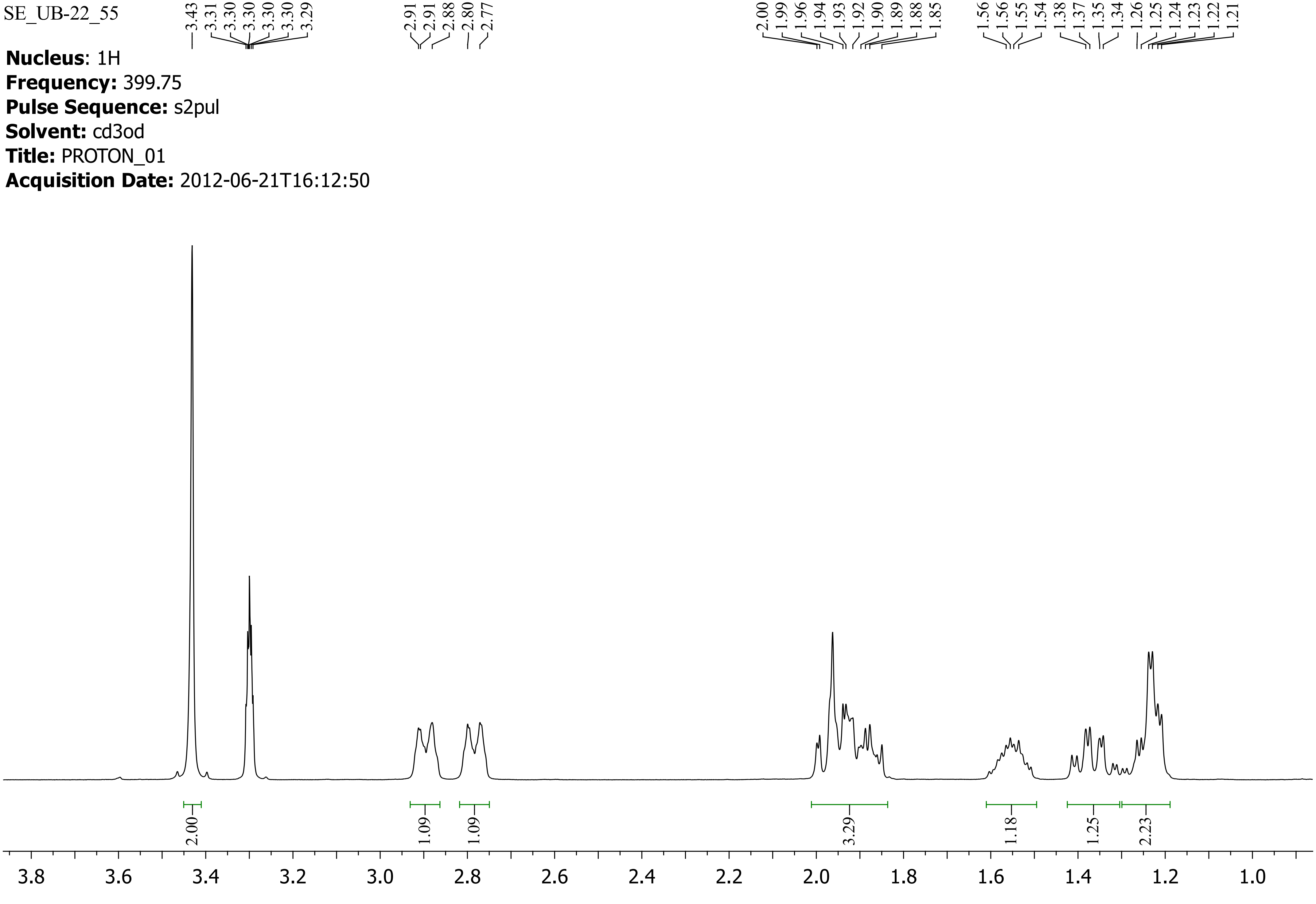

SE_UB-22_55

Nucleus: 13C
Frequency: 100.53
Pulse Sequence: s2pul
Solvent: cd3od
Title: CARBON_01
Acquisition Date: 2012-06-21T16:13:29

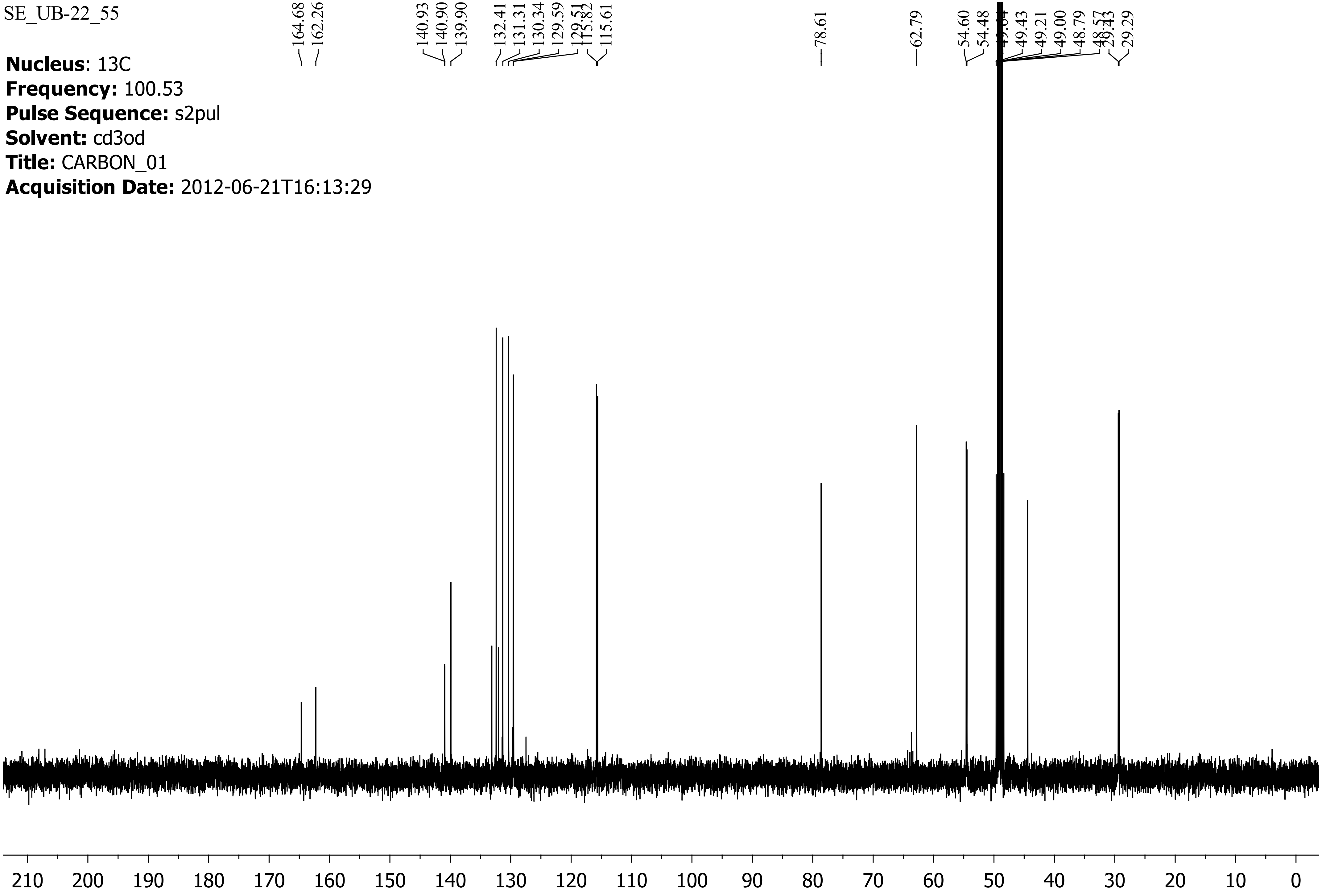

SE_UB-22_55

Nucleus: 13C
Frequency: 100.53
Pulse Sequence: s2pul
Solvent: cd3od
Title: CARBON_01
Acquisition Date: 2012-06-21T16:13:29

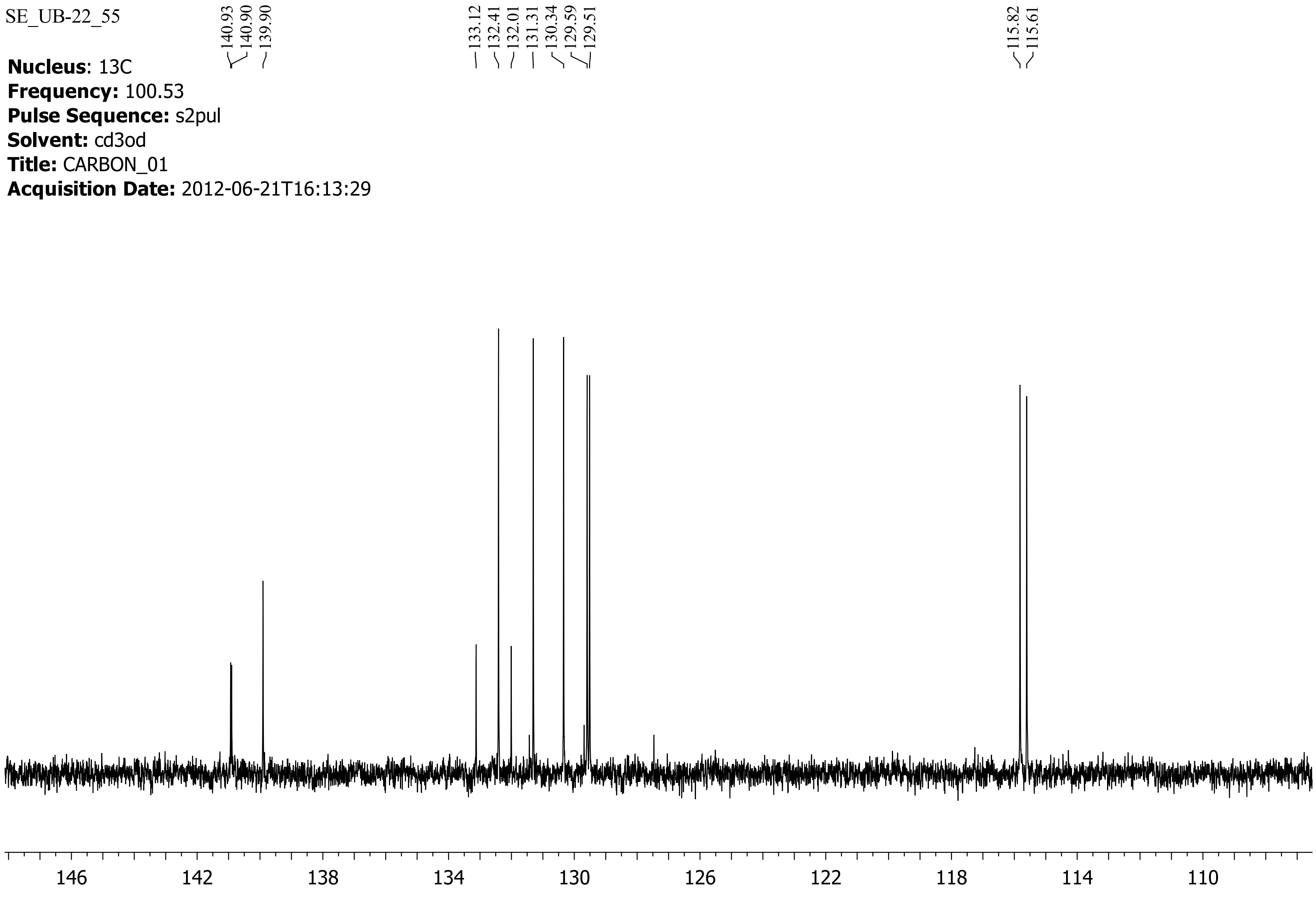

SE_UB-22_55

Nucleus: 13C
Frequency: 100.53
Pulse Sequence: s2pul
Solvent: cd3od
Title: CARBON_01
Acquisition Date: 2012-06-21T16:13:29

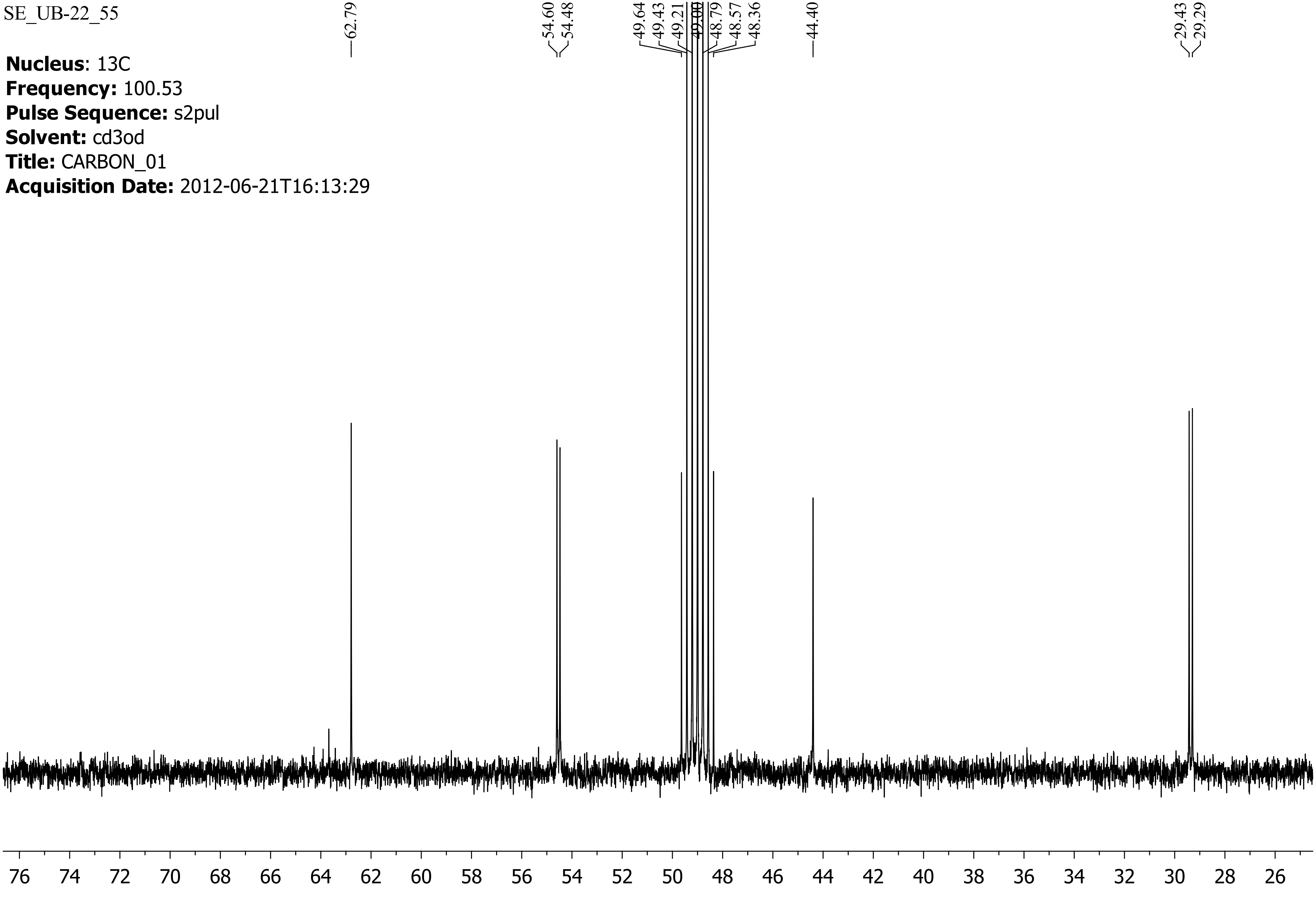

SE_UB-22_56

Nucleus: 1H
Frequency: 399.75
Pulse Sequence: s2pul
Solvent: cd3od
Title: PROTON_01
Acquisition Date: 2012-06-21T16:28:48

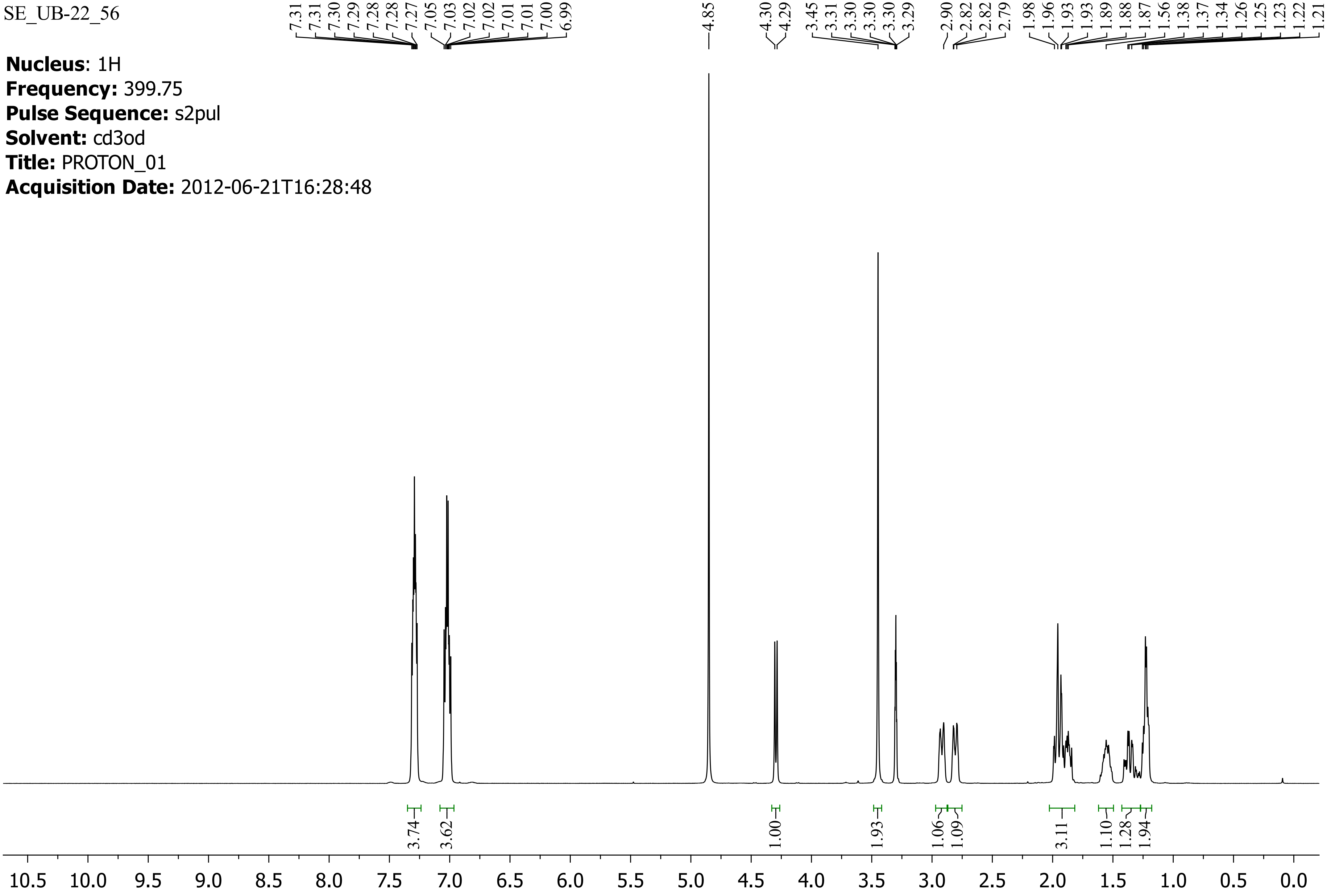

SE_UB-22_56

Nucleus: 1H
Frequency: 399.75
Pulse Sequence: s2pul
Solvent: cd3od
Title: PROTON_01
Acquisition Date: 2012-06-21T16:28:48

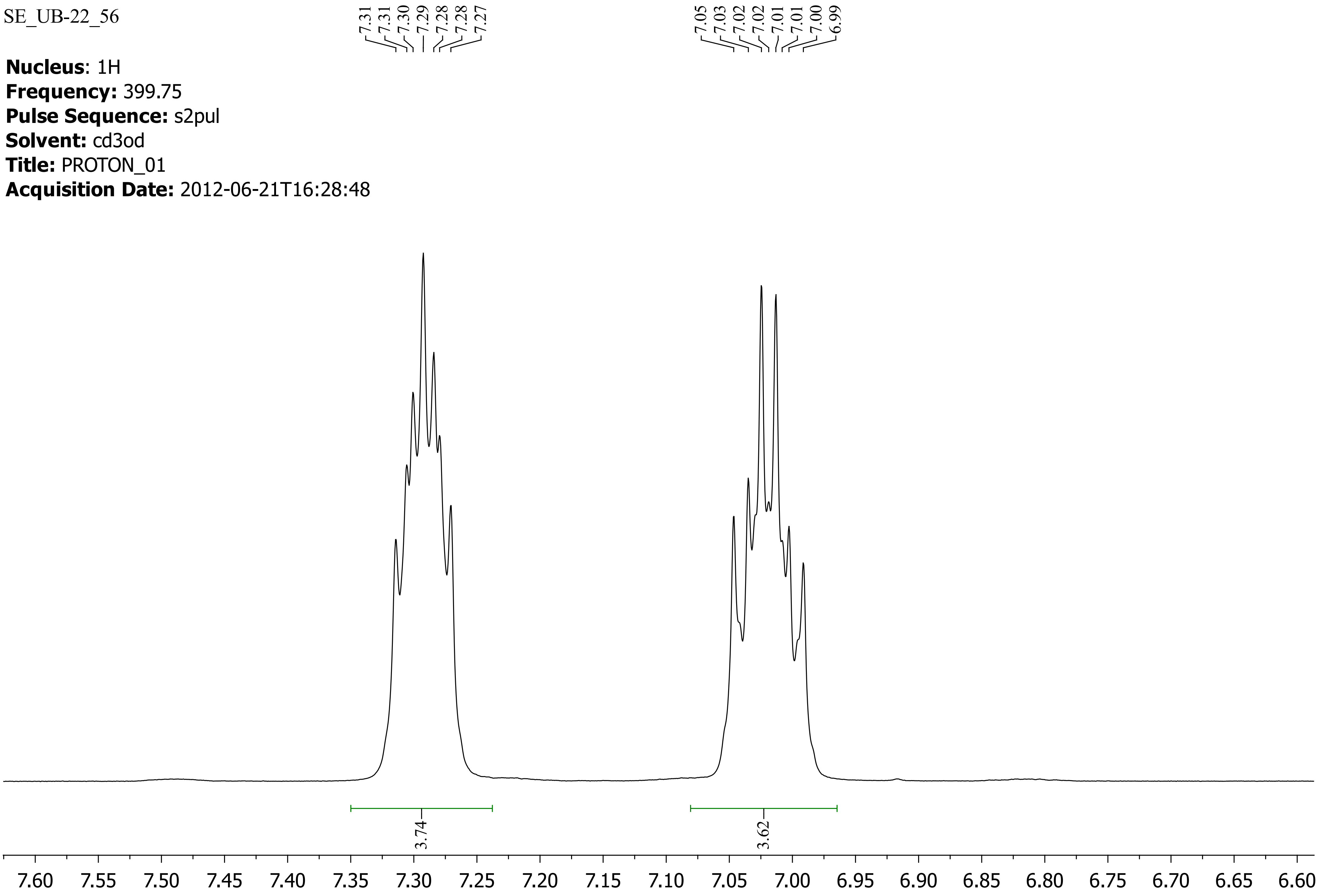

SE_UB-22_56

Nucleus: 1H
Frequency: 399.75
Pulse Sequence: s2pul
Solvent: cd3od
Title: PROTON_01
Acquisition Date: 2012-06-21T16:28:48

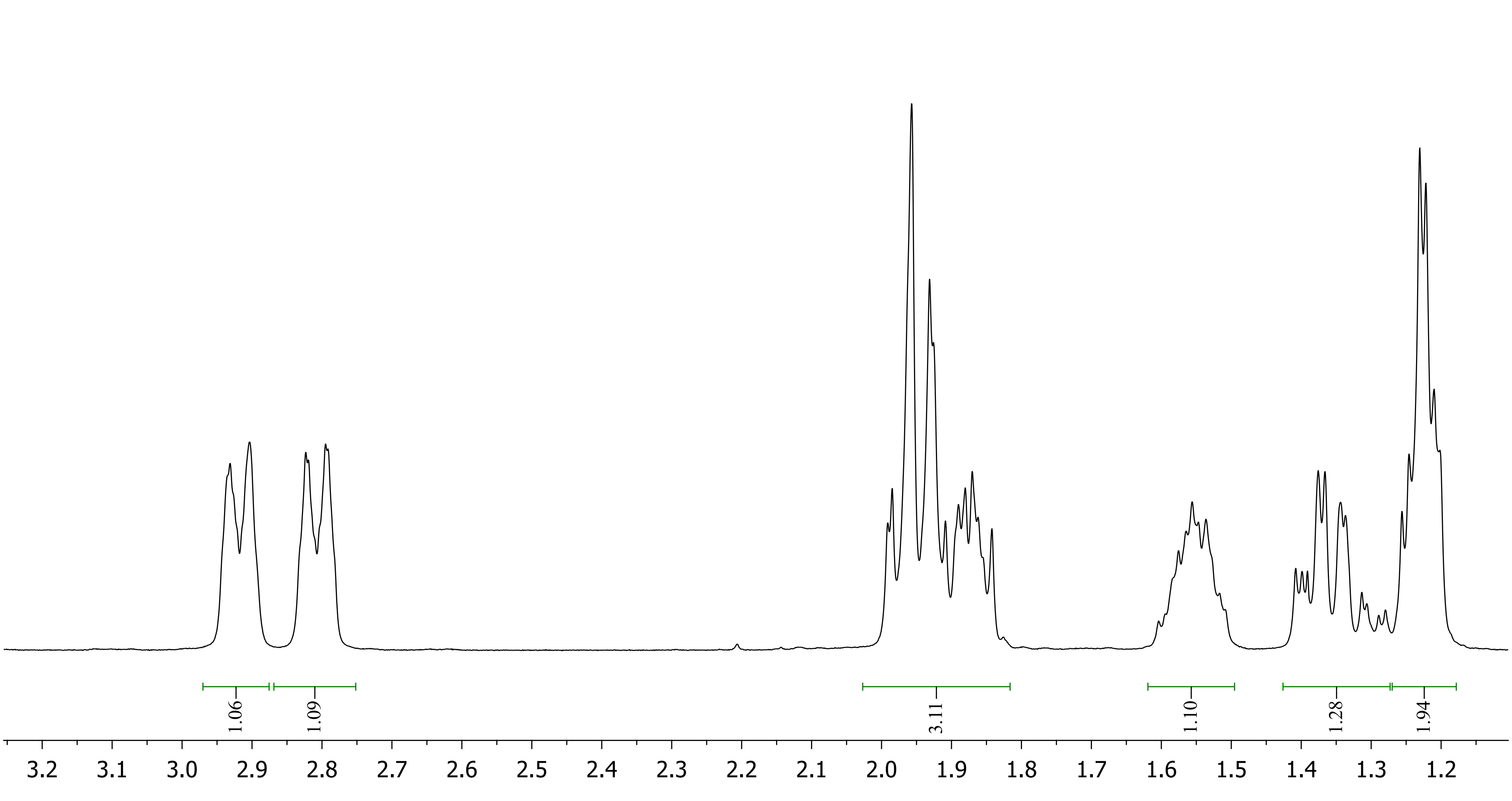

SE_UB-22_56

Nucleus: 13C
Frequency: 100.53
Pulse Sequence: s2pul
Solvent: cd3od
Title: CARBON_01
Acquisition Date: 2012-06-21T16:29:28

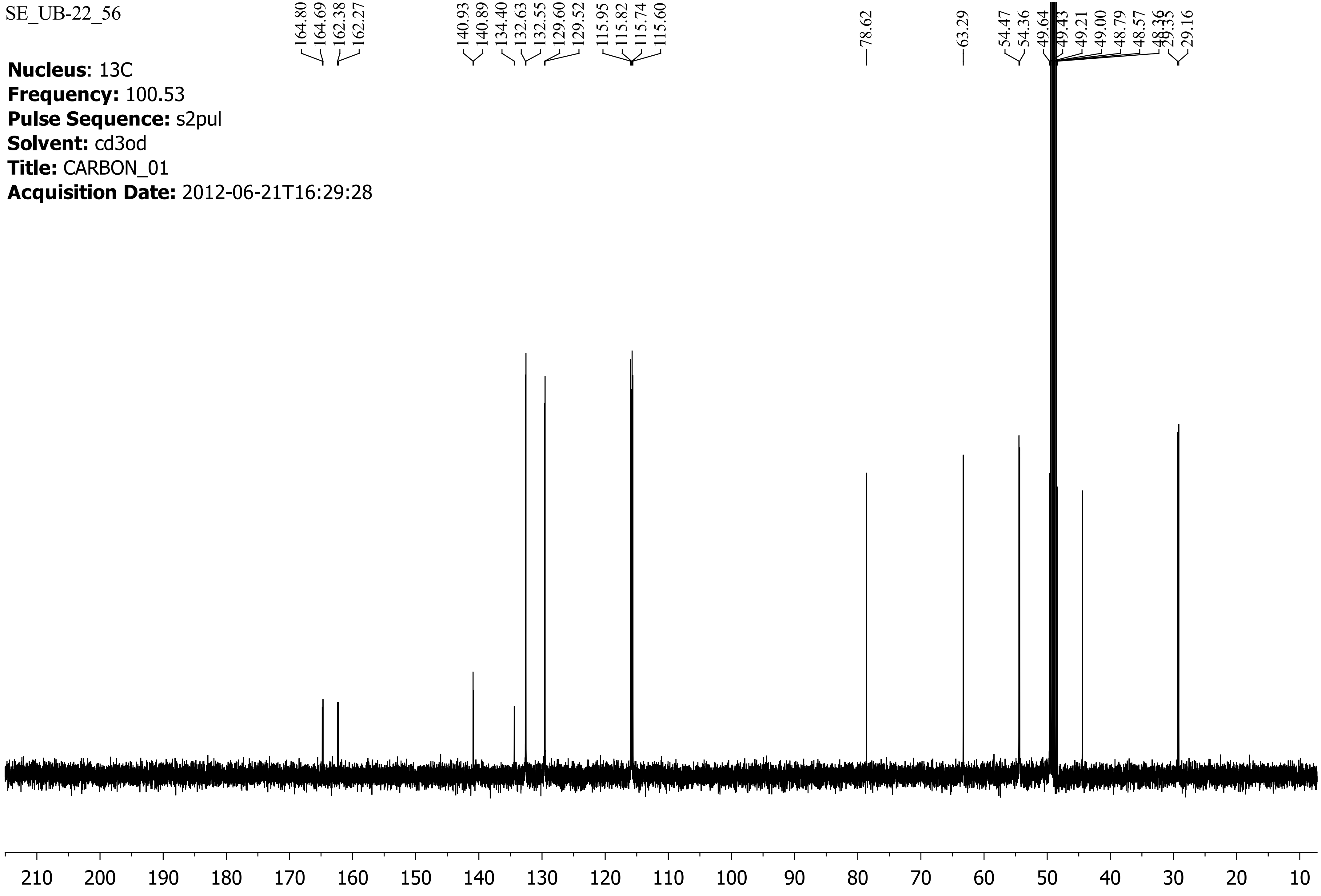

SE_UB-22_56

Nucleus: 13C
Frequency: 100.53
Pulse Sequence: s2pul
Solvent: cd3od
Title: CARBON_01
Acquisition Date: 2012-06-21T16:29:28

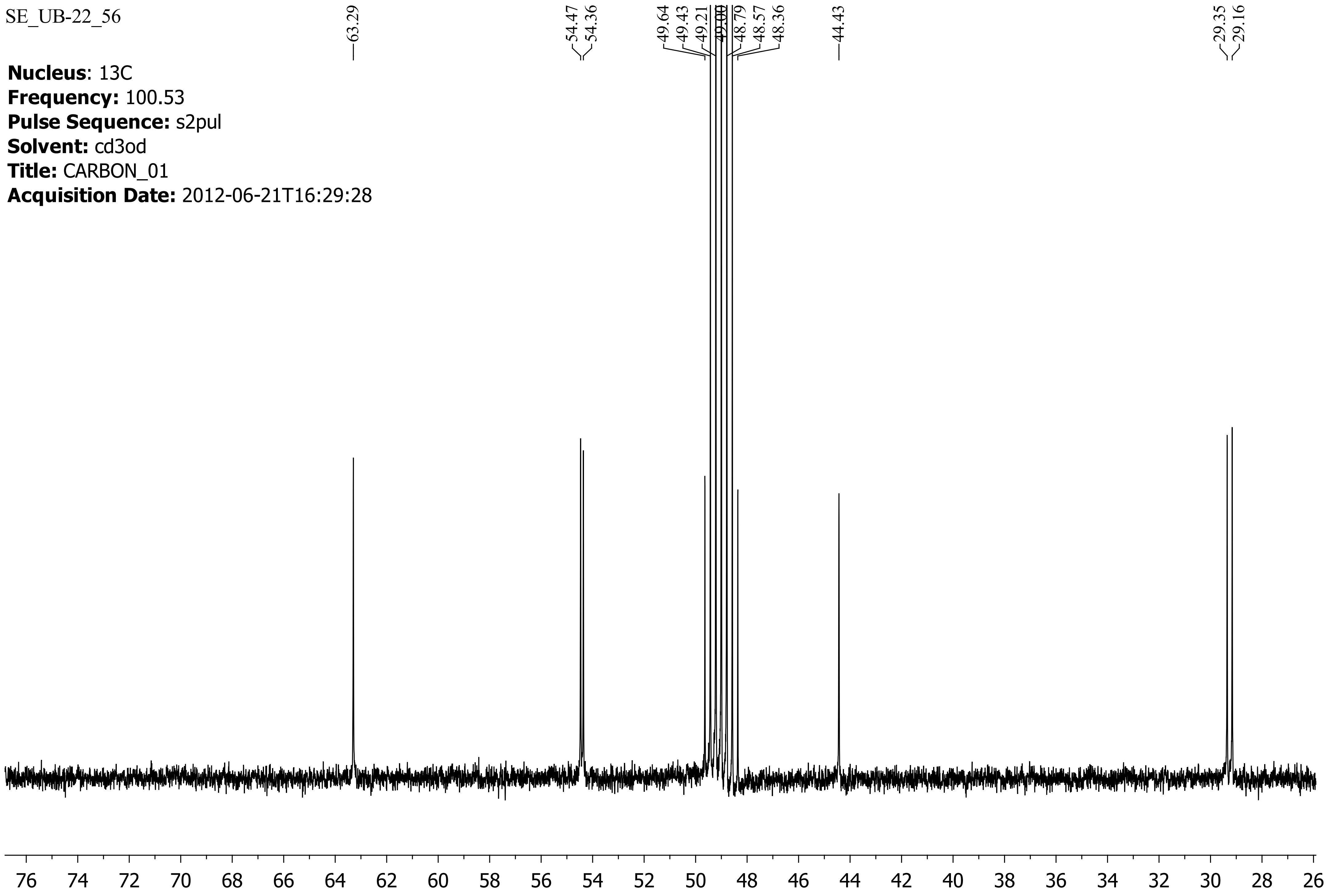

SE_UB-22_57

Nucleus: 1H
Frequency: 399.75
Pulse Sequence: s2pul
Solvent: cd3od
Title: PROTON_01
Acquisition Date: 2012-06-21T16:48:48

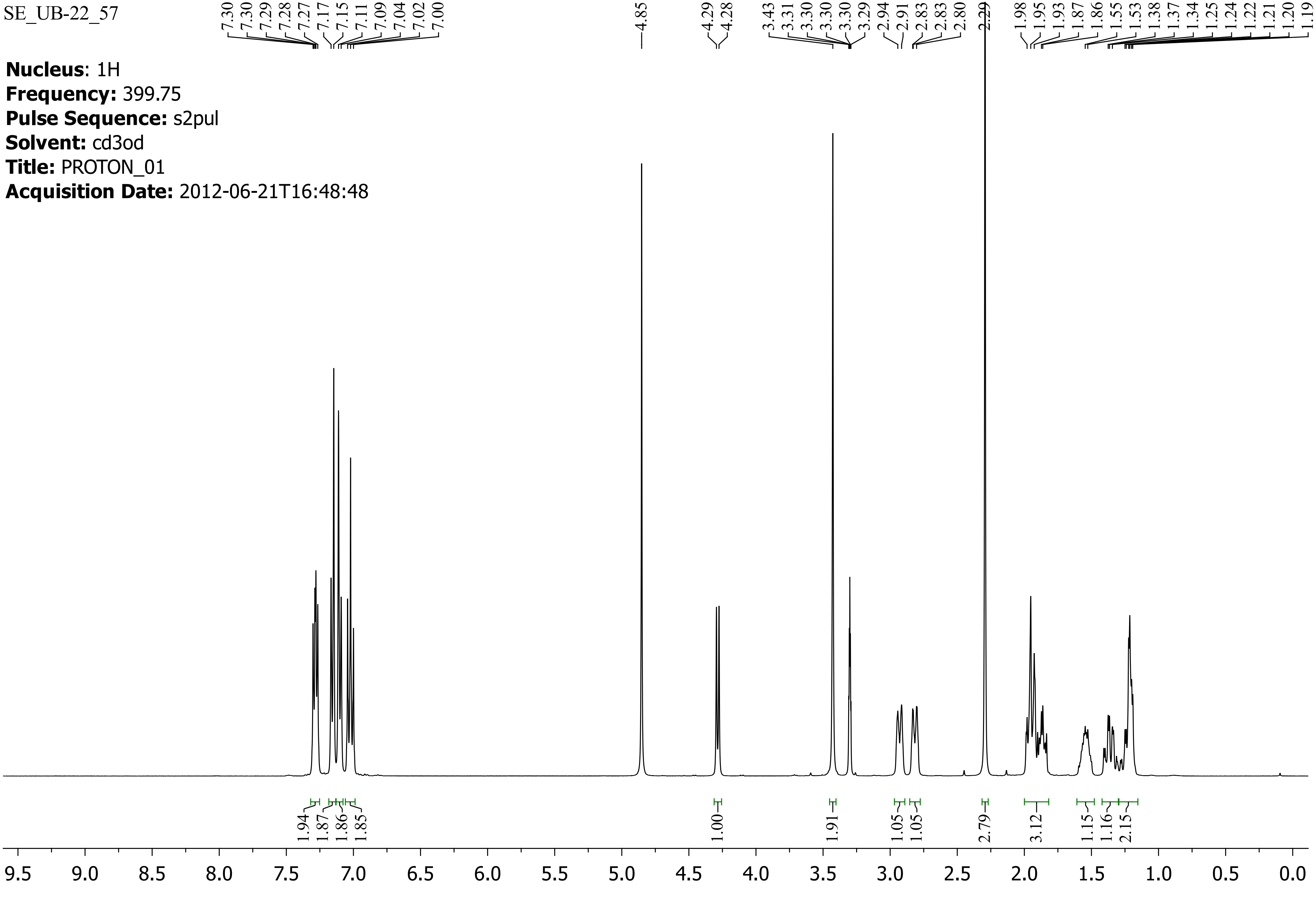

SE_UB-22_57

Nucleus: 1H
Frequency: 399.75
Pulse Sequence: s2pul
Solvent: cd3od
Title: PROTON_01
Acquisition Date: 2012-06-21T16:48:48

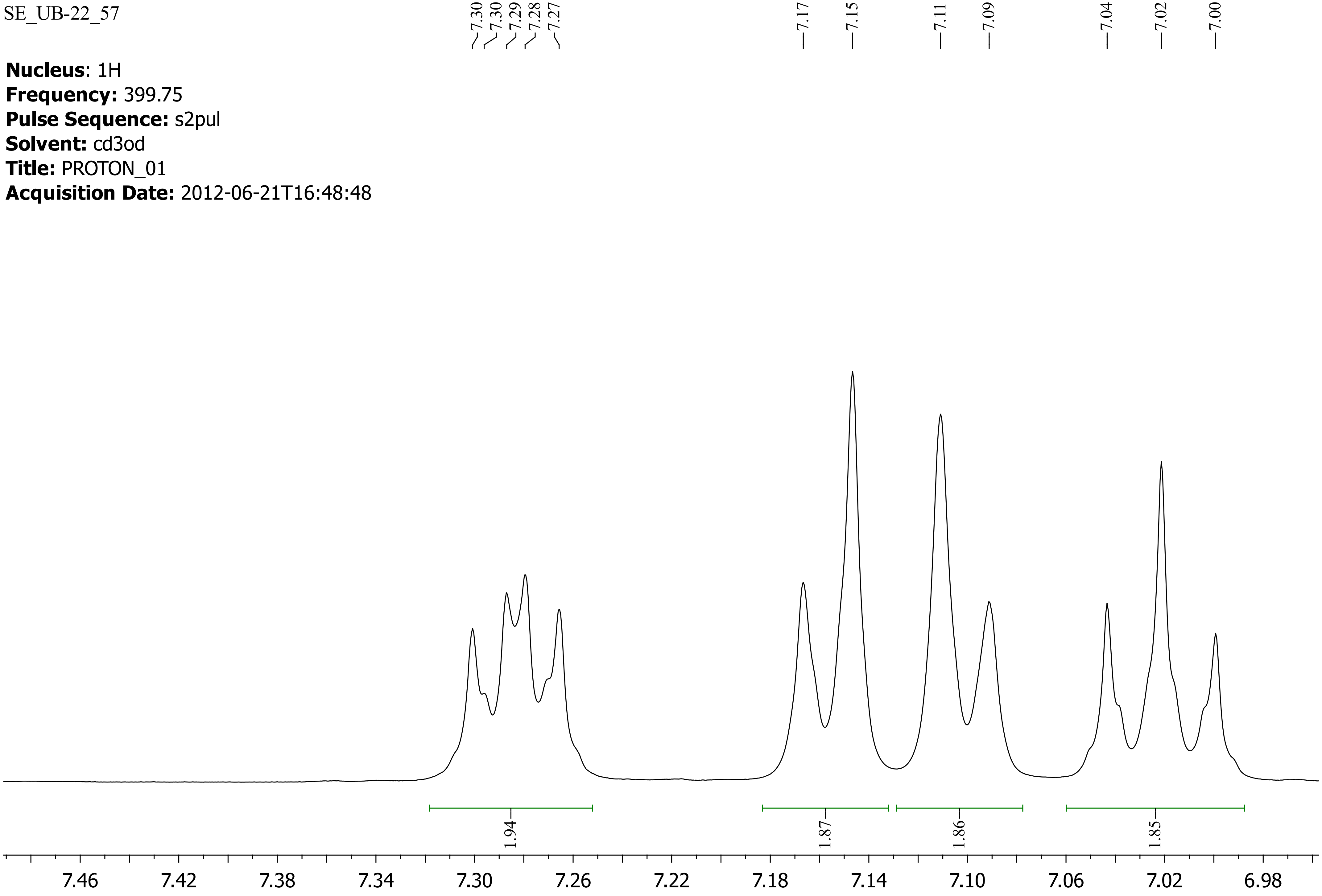

SE_UB-22_57

Nucleus: 1H
Frequency: 399.75
Pulse Sequence: s2pul
Solvent: cd3od
Title: PROTON_01
Acquisition Date: 2012-06-21T16:48:48

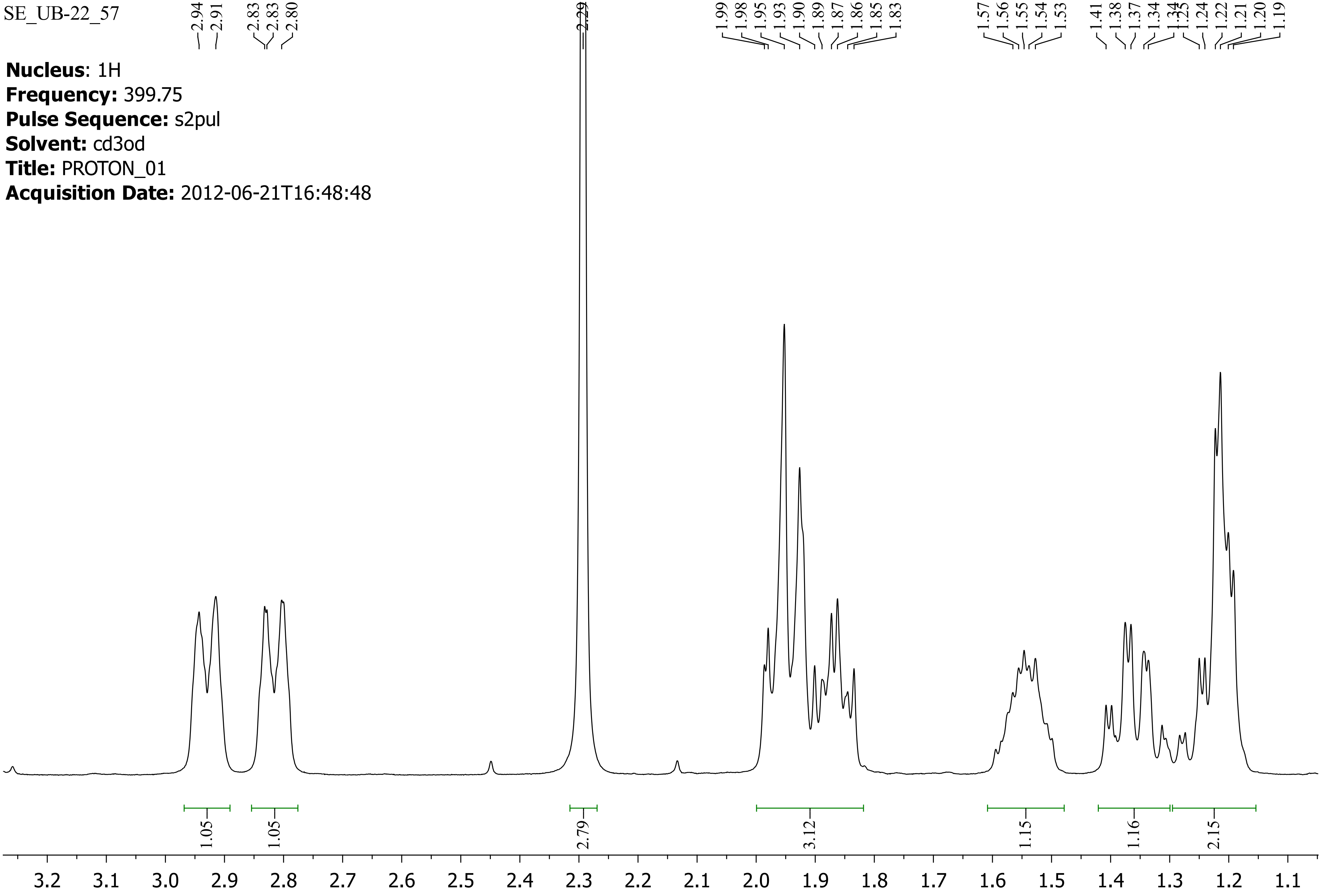

SE_UB-22_57

Nucleus: 13C
Frequency: 100.53
Pulse Sequence: s2pul
Solvent: cd3od
Title: CARBON_01
Acquisition Date: 2012-06-21T16:49:27

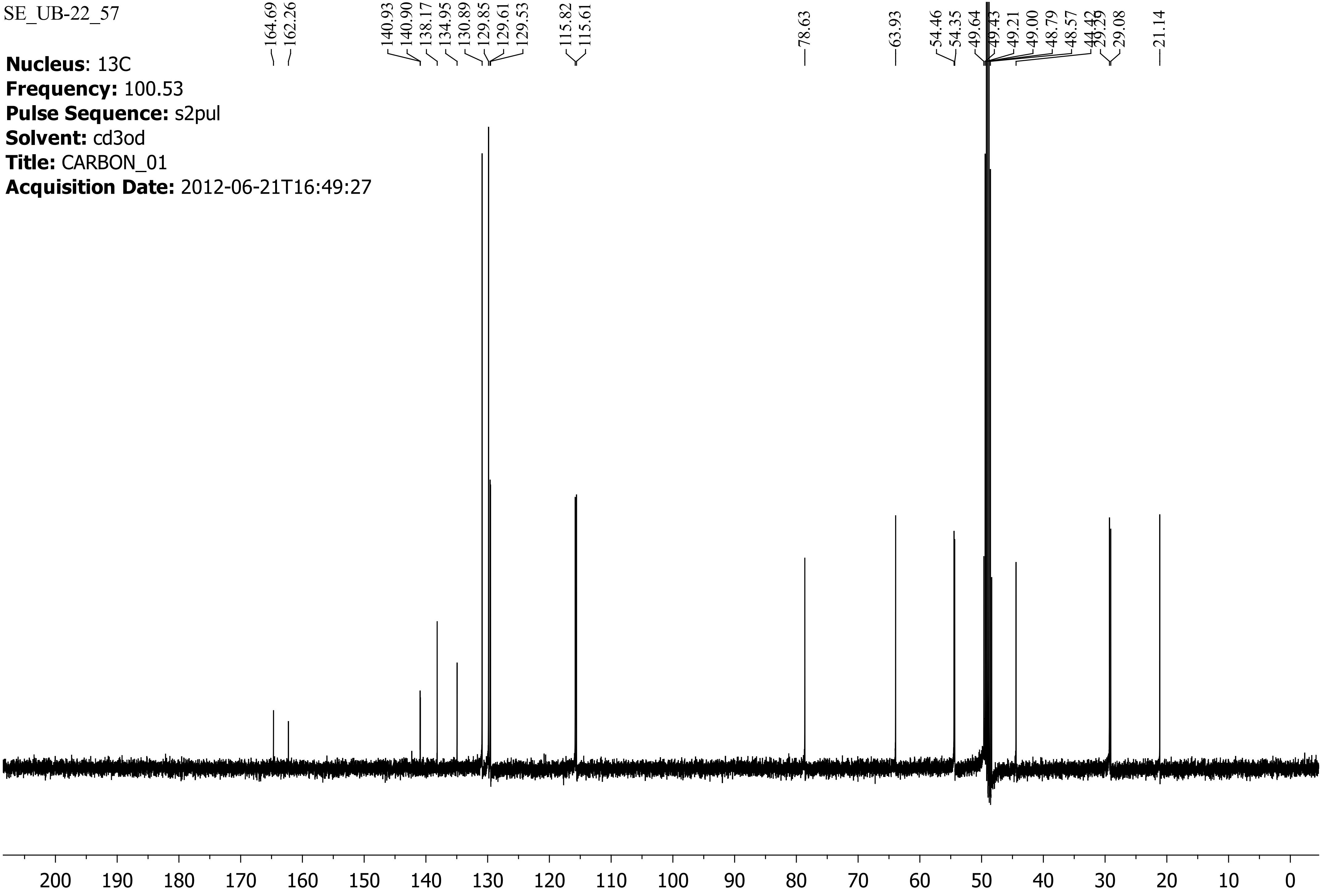

SE_UB-22_57

Nucleus: 13C
Frequency: 100.53
Pulse Sequence: s2pul
Solvent: cd3od
Title: CARBON_01
Acquisition Date: 2012-06-21T16:49:27

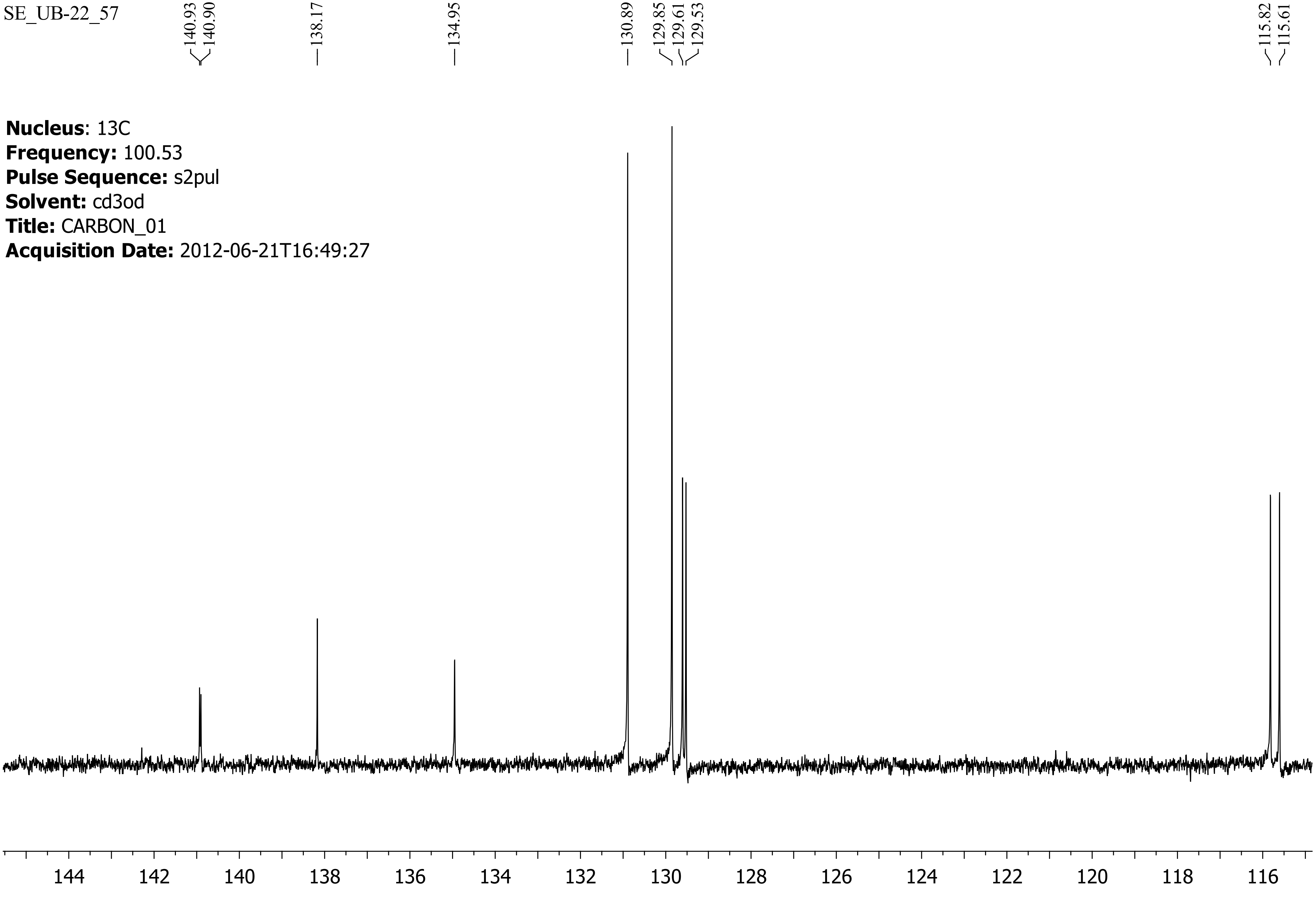

SE_UB-22_57

Nucleus: 13C
Frequency: 100.53
Pulse Sequence: s2pul
Solvent: cd3od
Title: CARBON_01
Acquisition Date: 2012-06-21T16:49:27

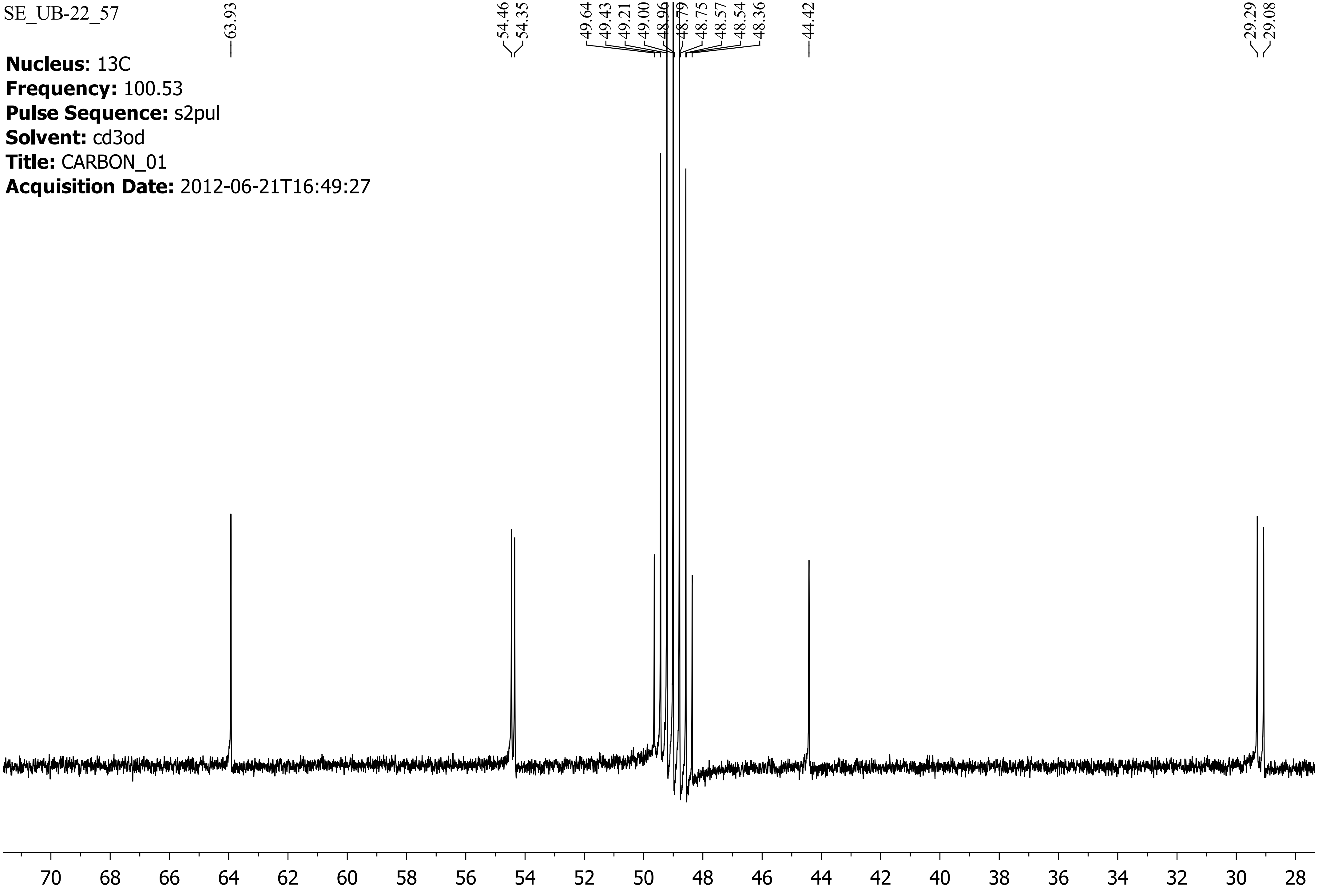

SE_UB-22_59

Nucleus: 1H
Frequency: 399.75
Pulse Sequence: s2pul
Solvent: cdcl3
Title: PROTON_01
Acquisition Date: 2012-06-21T18:23:28

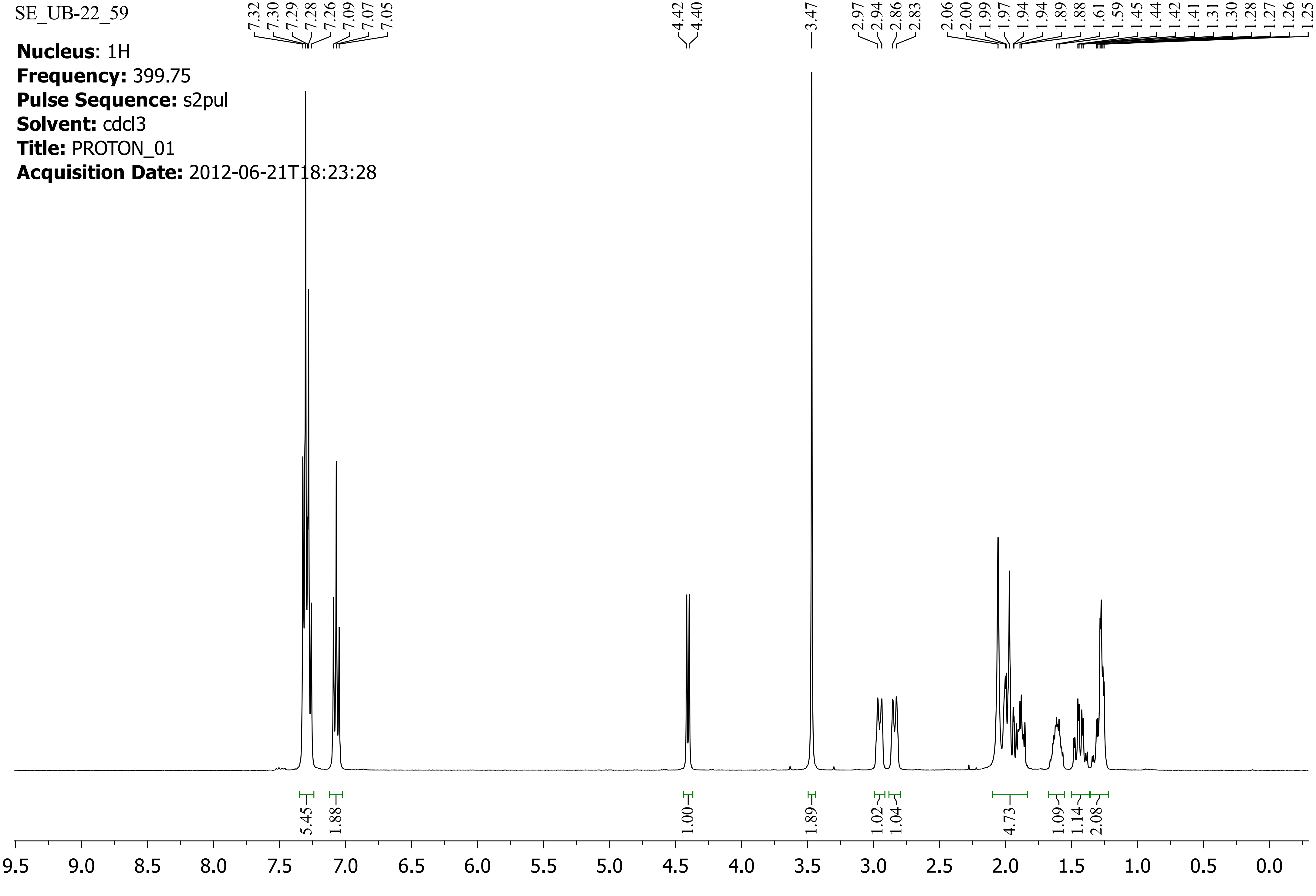

SE_UB-22_59

Nucleus: 1H
Frequency: 399.75
Pulse Sequence: s2pul
Solvent: cdcl3
Title: PROTON_01
Acquisition Date: 2012-06-21T18:23:28

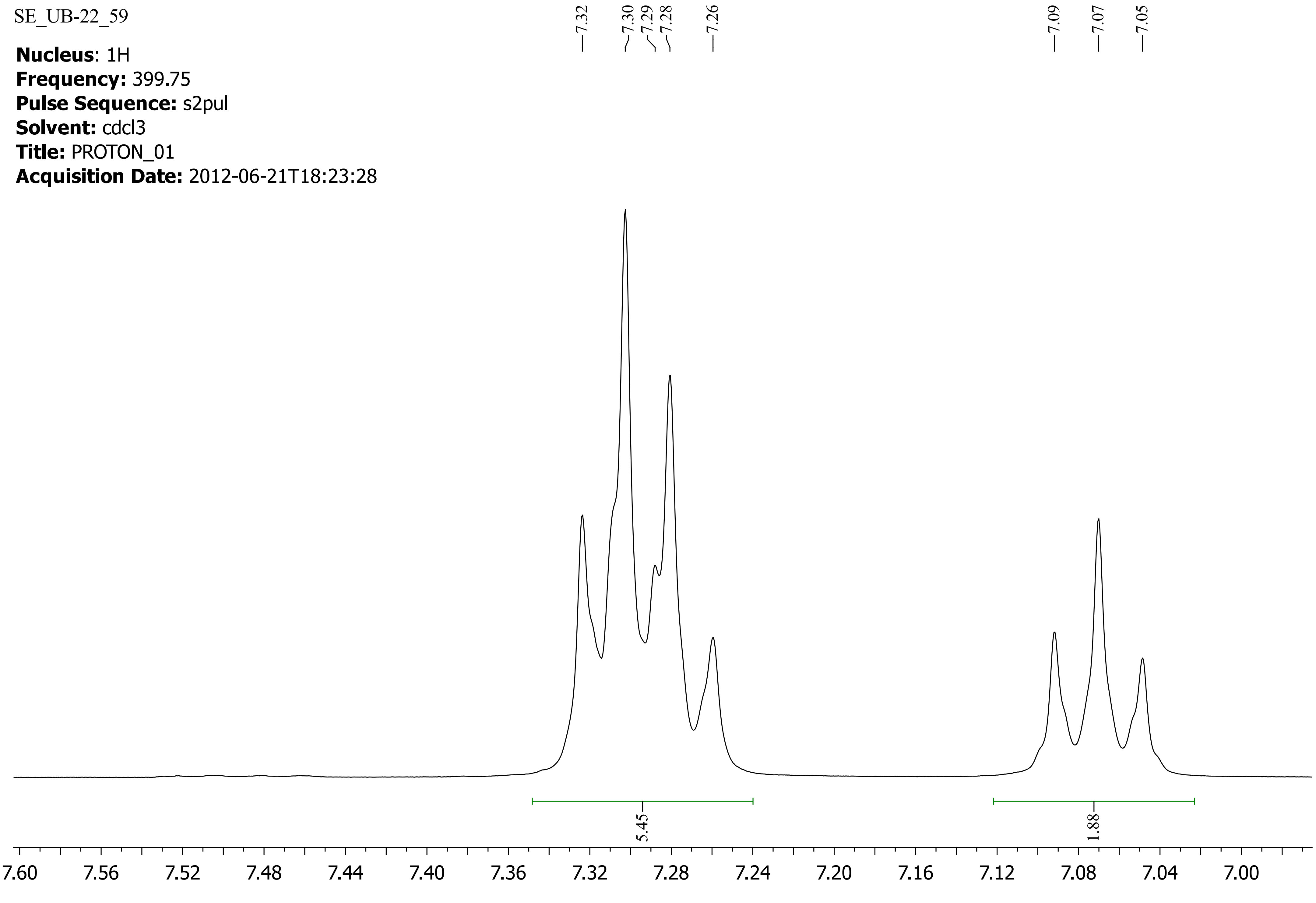

SE_UB-22_59

Nucleus: 1H
Frequency: 399.75
Pulse Sequence: s2pul
Solvent: cdcl3
Title: PROTON_01
Acquisition Date: 2012-06-21T18:23:28

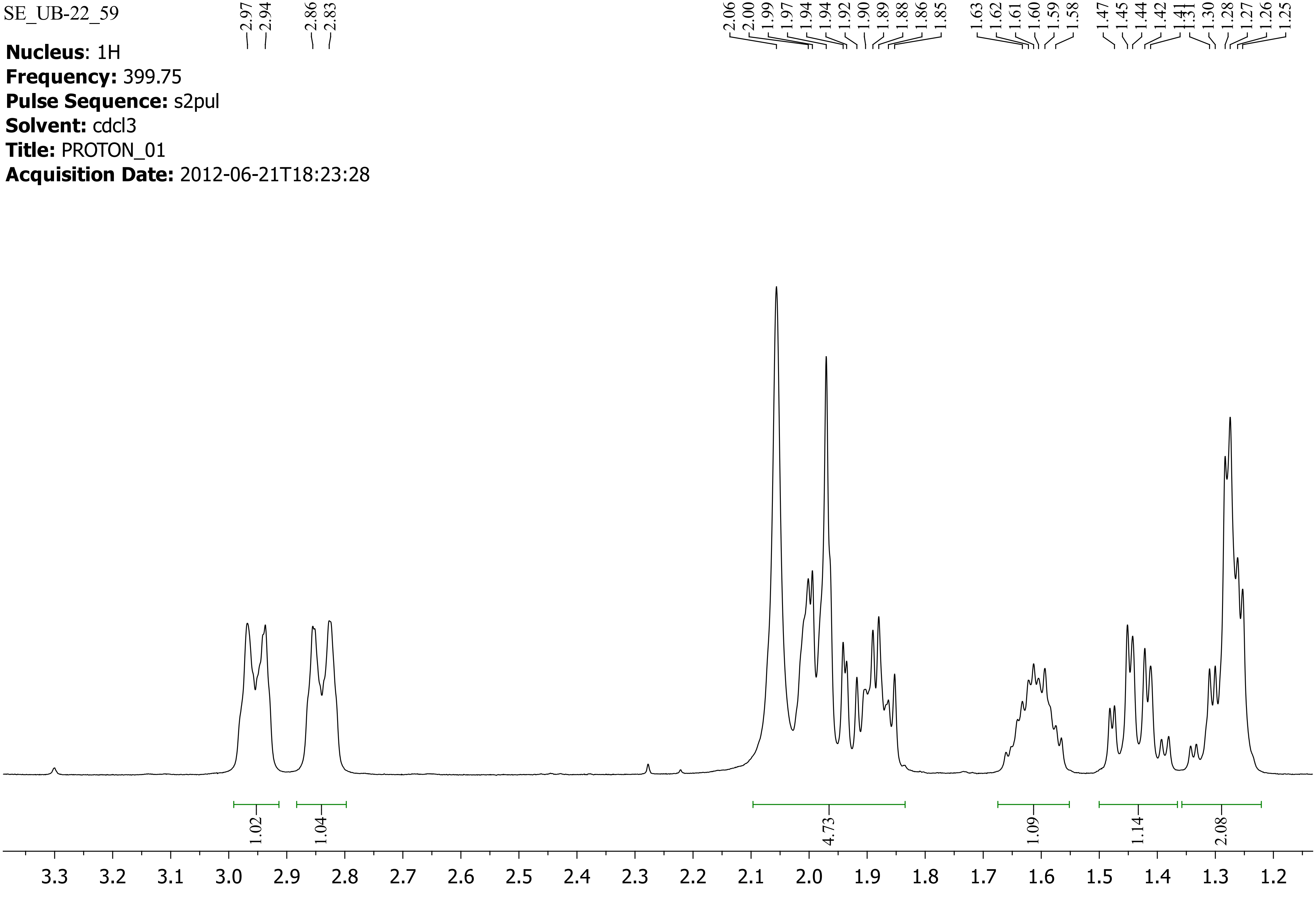

SE_UB-22_59

Nucleus: 13C
Frequency: 100.53
Pulse Sequence: s2pul
Solvent: cdcl3
Title: CARBON_01
Acquisition Date: 2012-06-21T18:24:07

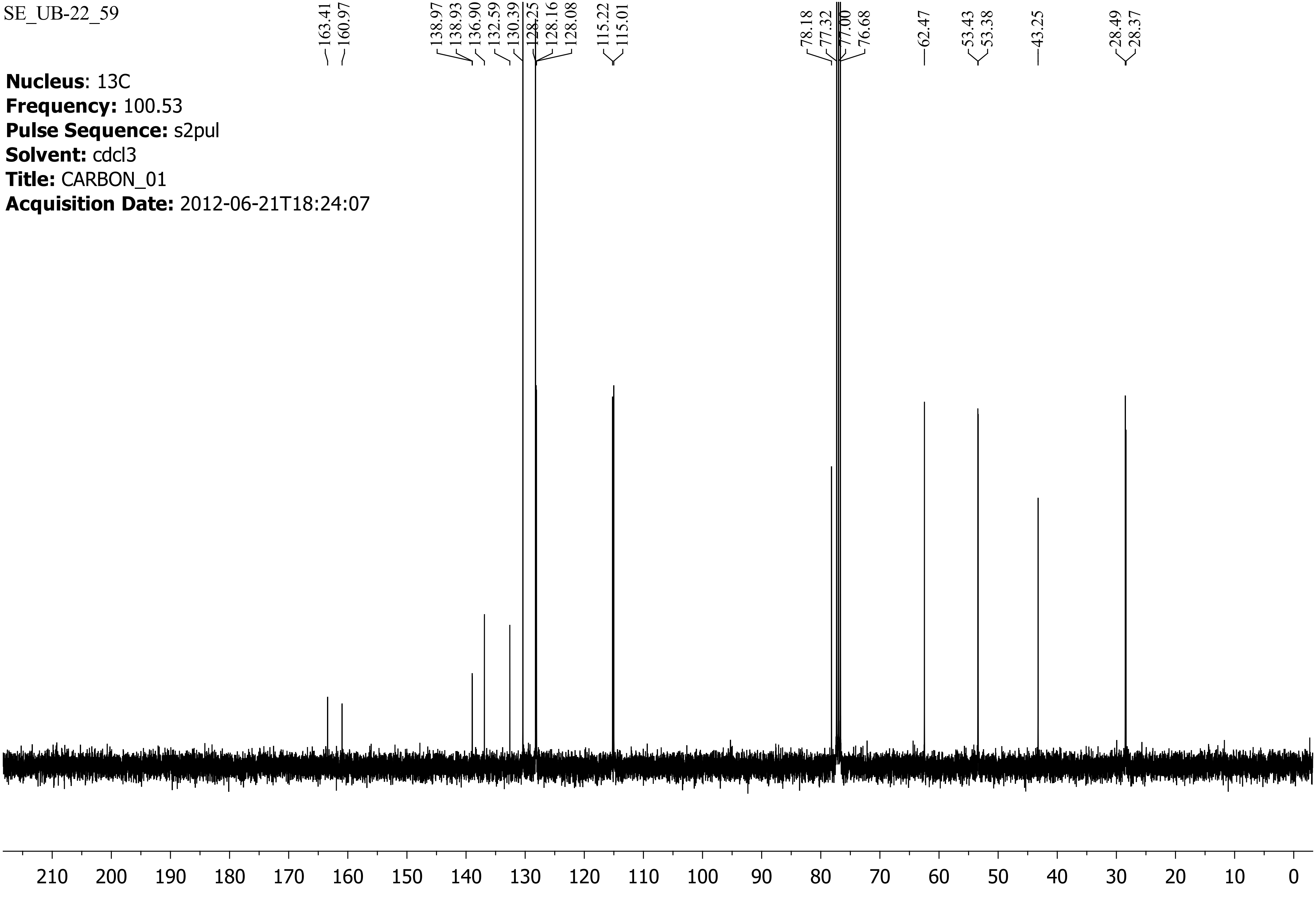

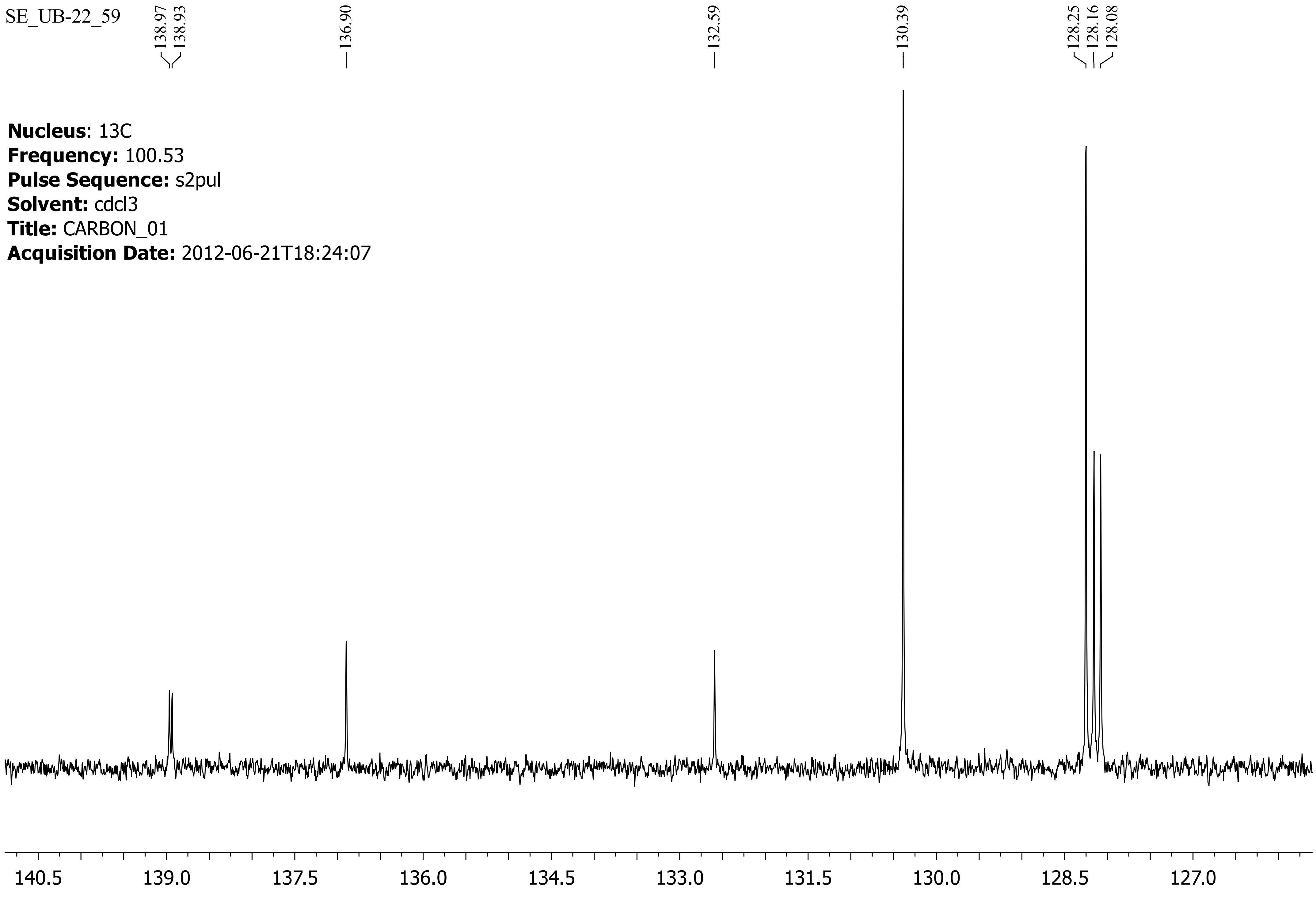

SE_UB-22_59
138.97
138.93
136.90
132.59
130.39
128.25
128.16
128.08
Nucleus: 13C
Frequency: 100.53
Pulse Sequence: s2pul
Solvent: cdcl3
Title: CARBON_01
Acquisition Date: 2012-06-21T18:24:07
140.5
139.0
137.5
136.0
134.5
133.0
131.5
130.0
128.5
127.0

SE_UB-22_60

Nucleus: 1H
Frequency: 399.75
Pulse Sequence: s2pul
Solvent: cd3od
Title: PROTON_01
Acquisition Date: 2012-06-21T17:04:20

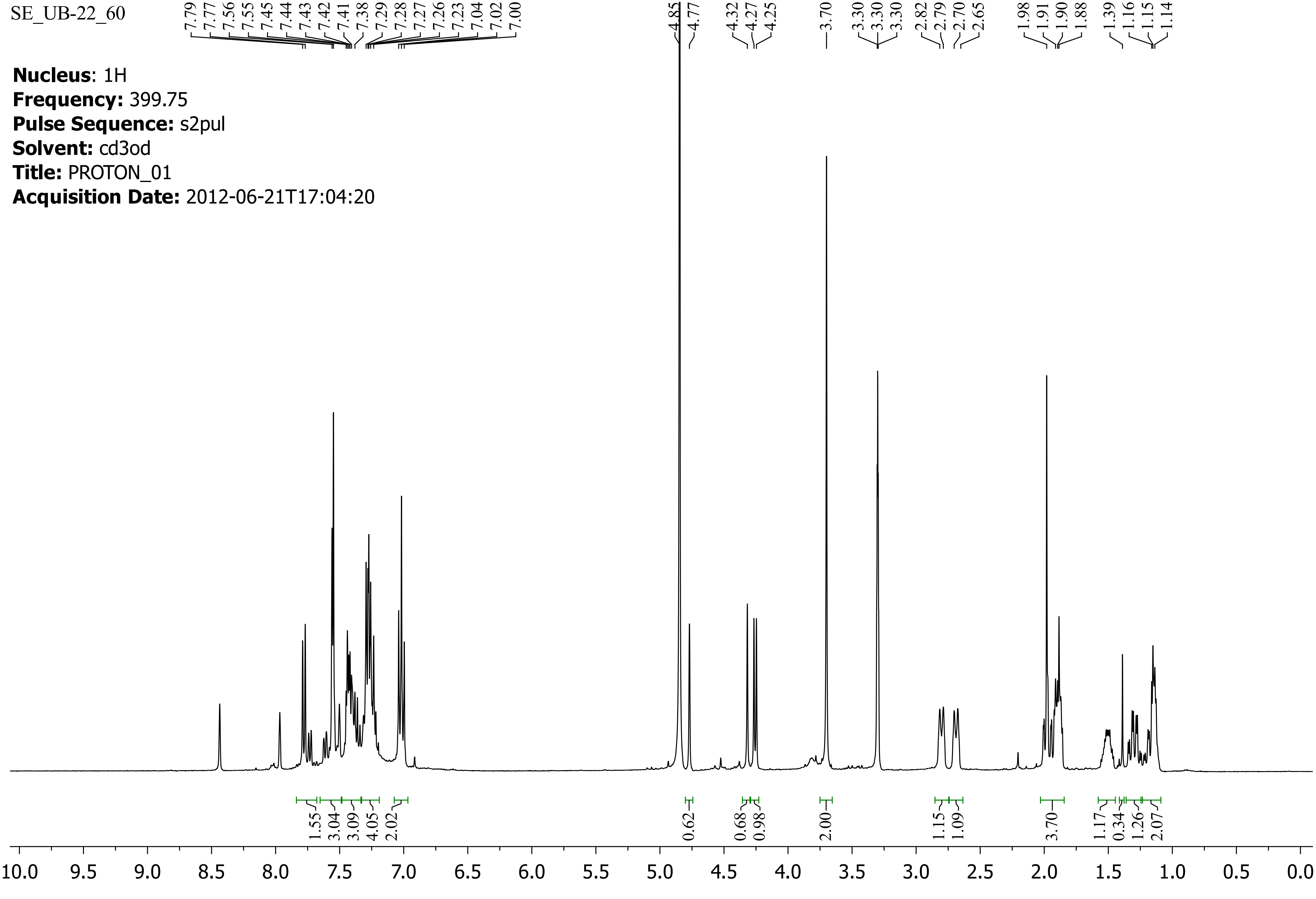

SE_UB-22_60

Nucleus: 1H
Frequency: 399.75
Pulse Sequence: s2pul
Solvent: cd3od
Title: PROTON_01
Acquisition Date: 2012-06-21T17:04:20

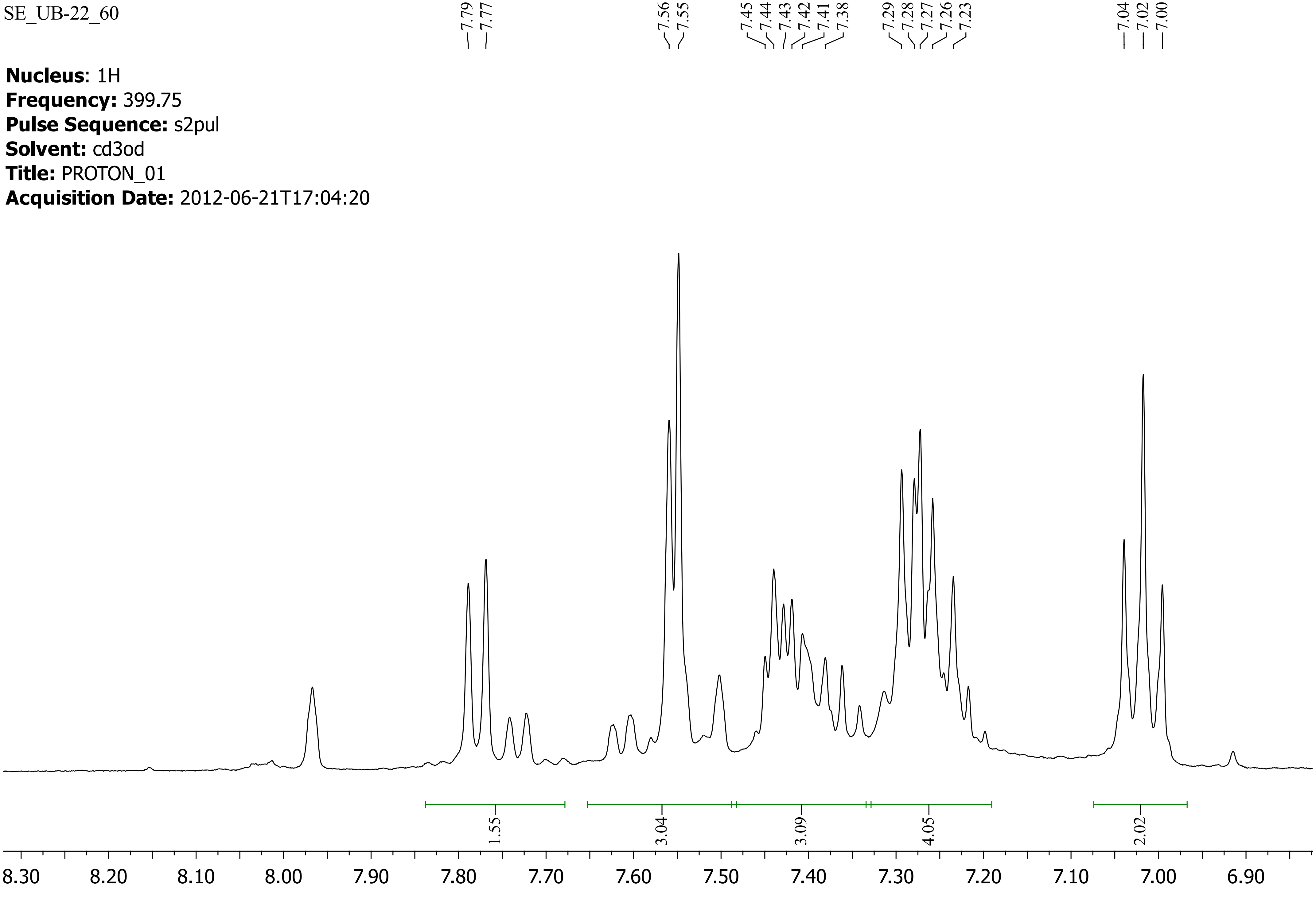

SE_UB-22_60

Nucleus: 1H
Frequency: 399.75
Pulse Sequence: s2pul
Solvent: cd3od
Title: PROTON_01
Acquisition Date: 2012-06-21T17:04:20

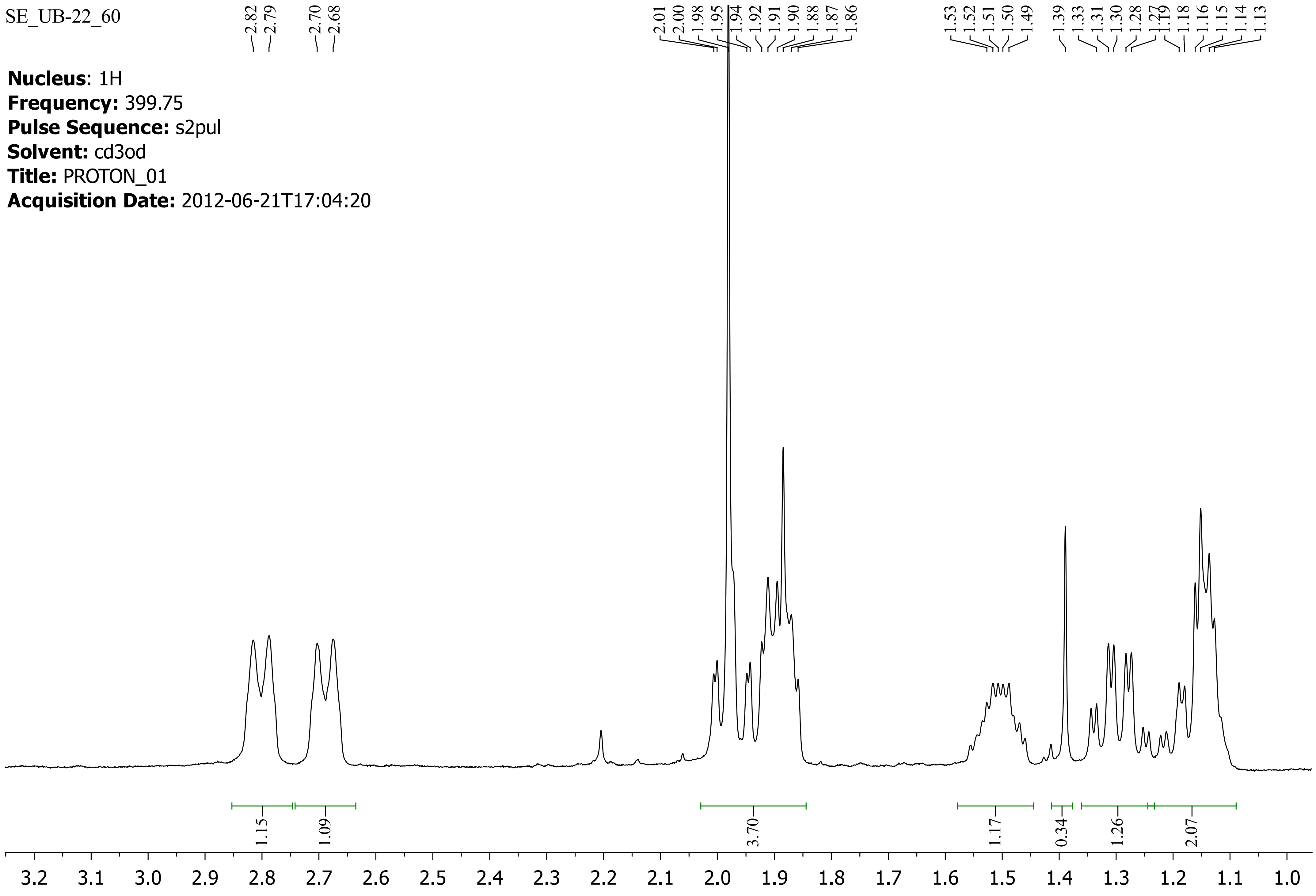

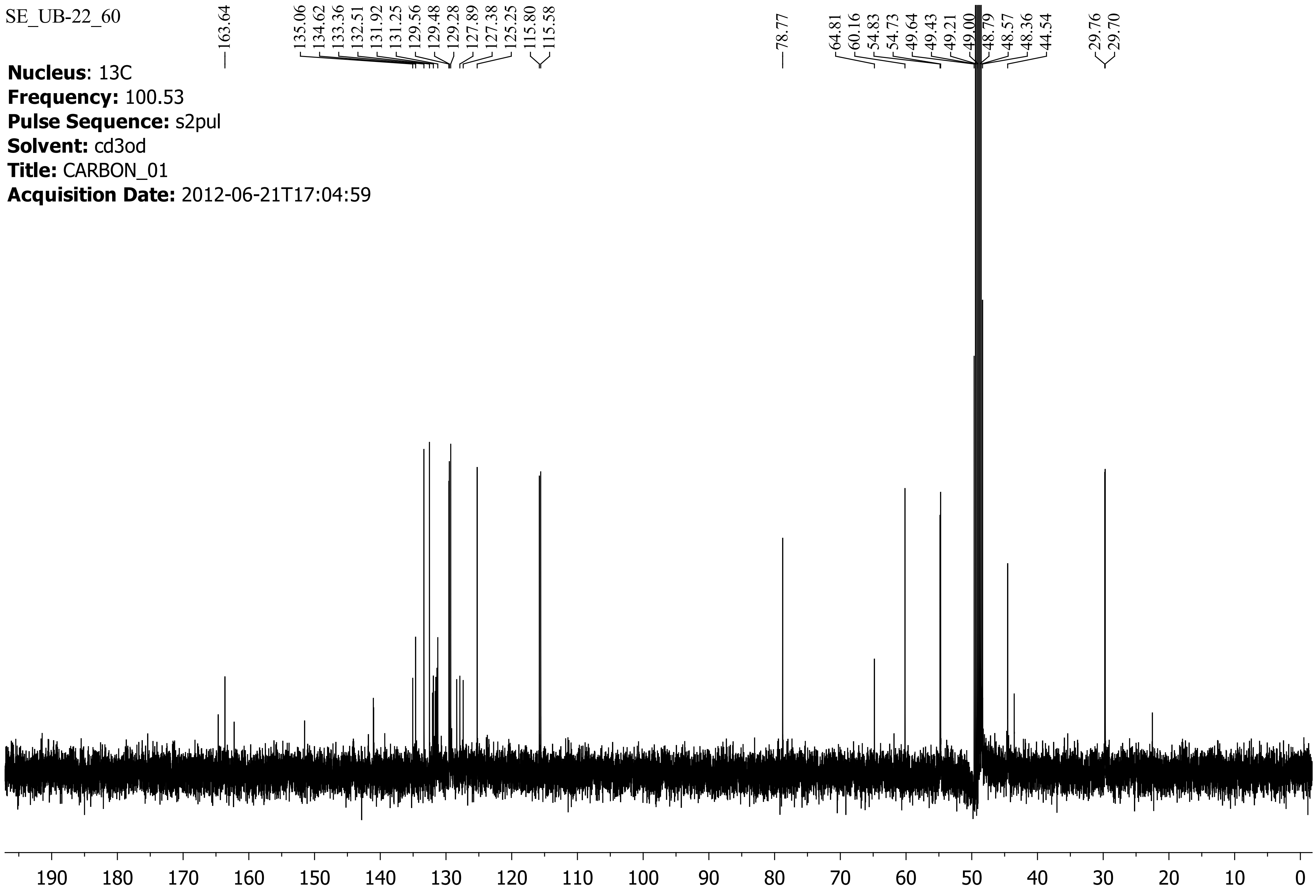
SE_UB-22_60
163.64
135.06
134.62
133.36
132.51
131.92
131.25
129.56
129.48
129.28
127.89
127.38
125.25
115.80
115.58
78.77
64.81
60.16
54.83
54.73
49.64
49.43
49.21
49.00
48.79
48.57
48.36
44.54
29.76
29.70
Nucleus: 13C
Frequency: 100.53
Pulse Sequence: s2pul
Solvent: cd3od
Title: CARBON_01
Acquisition Date: 2012-06-21T17:04:59
190 180 170 160 150 140 130 120 110 100 90 80 70 60 50 40 30 20 10 0

SE_UB-22_60

Nucleus: 13C
Frequency: 100.53
Pulse Sequence: s2pul
Solvent: cd3od
Title: CARBON_01
Acquisition Date: 2012-06-21T17:04:59

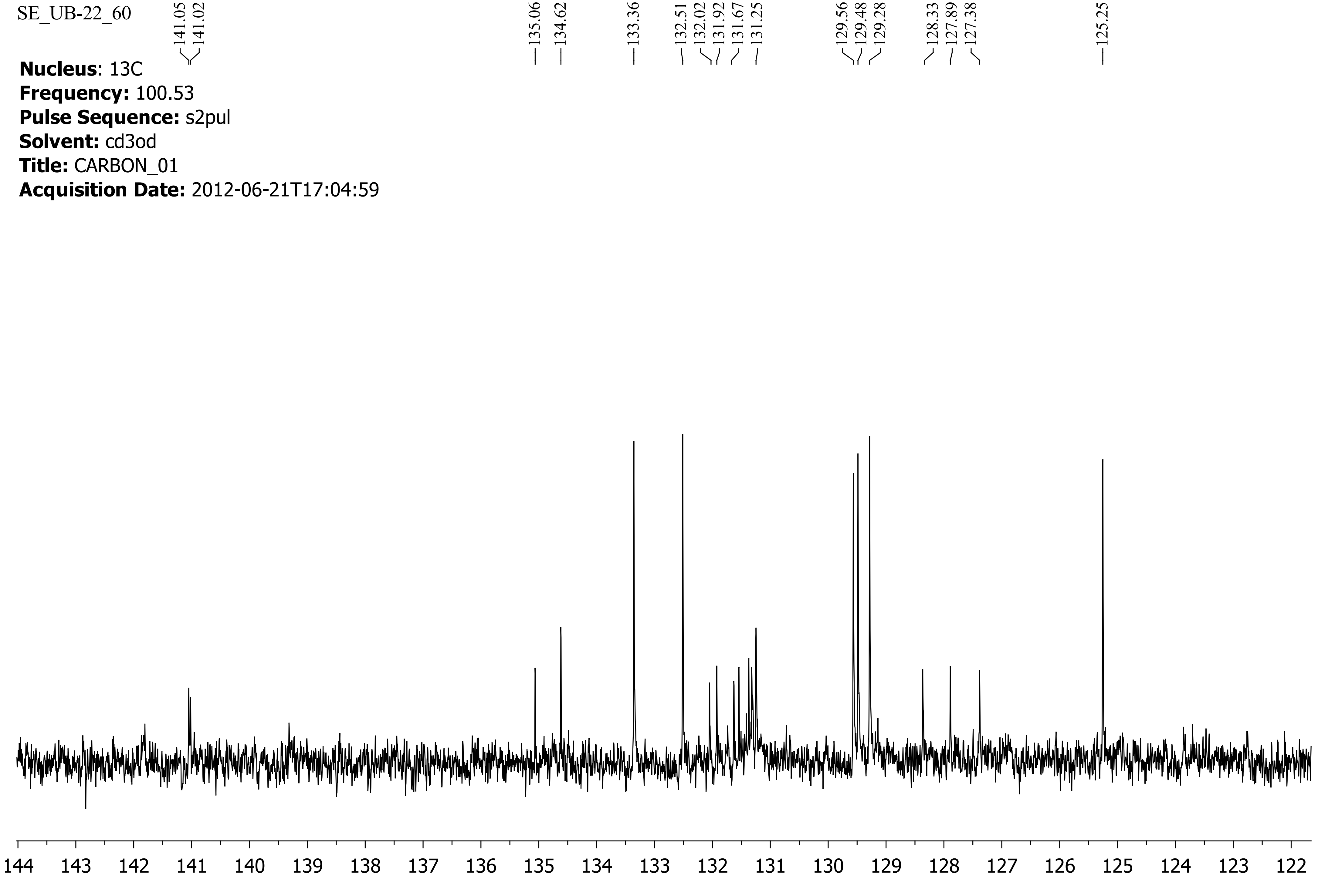

SE_UB-22_61

Nucleus: 1H
Frequency: 399.75
Pulse Sequence: s2pul
Solvent: cd3od
Title: PROTON_01
Acquisition Date: 2012-06-21T17:19:47

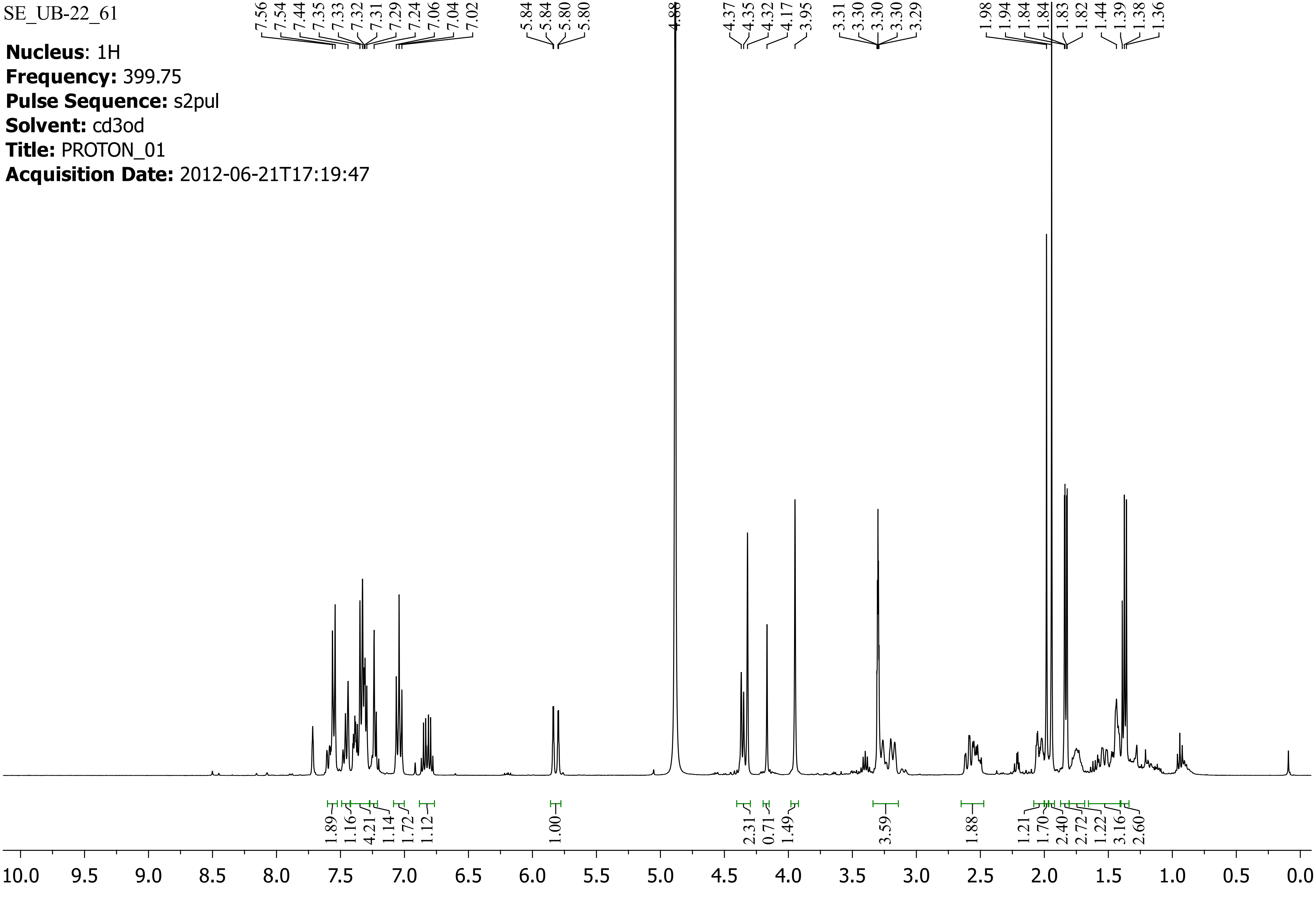

SE_UB-22_61

Nucleus: 13C
Frequency: 100.53
Pulse Sequence: s2pul
Solvent: cd3od
Title: CARBON_01
Acquisition Date: 2012-06-21T17:20:26

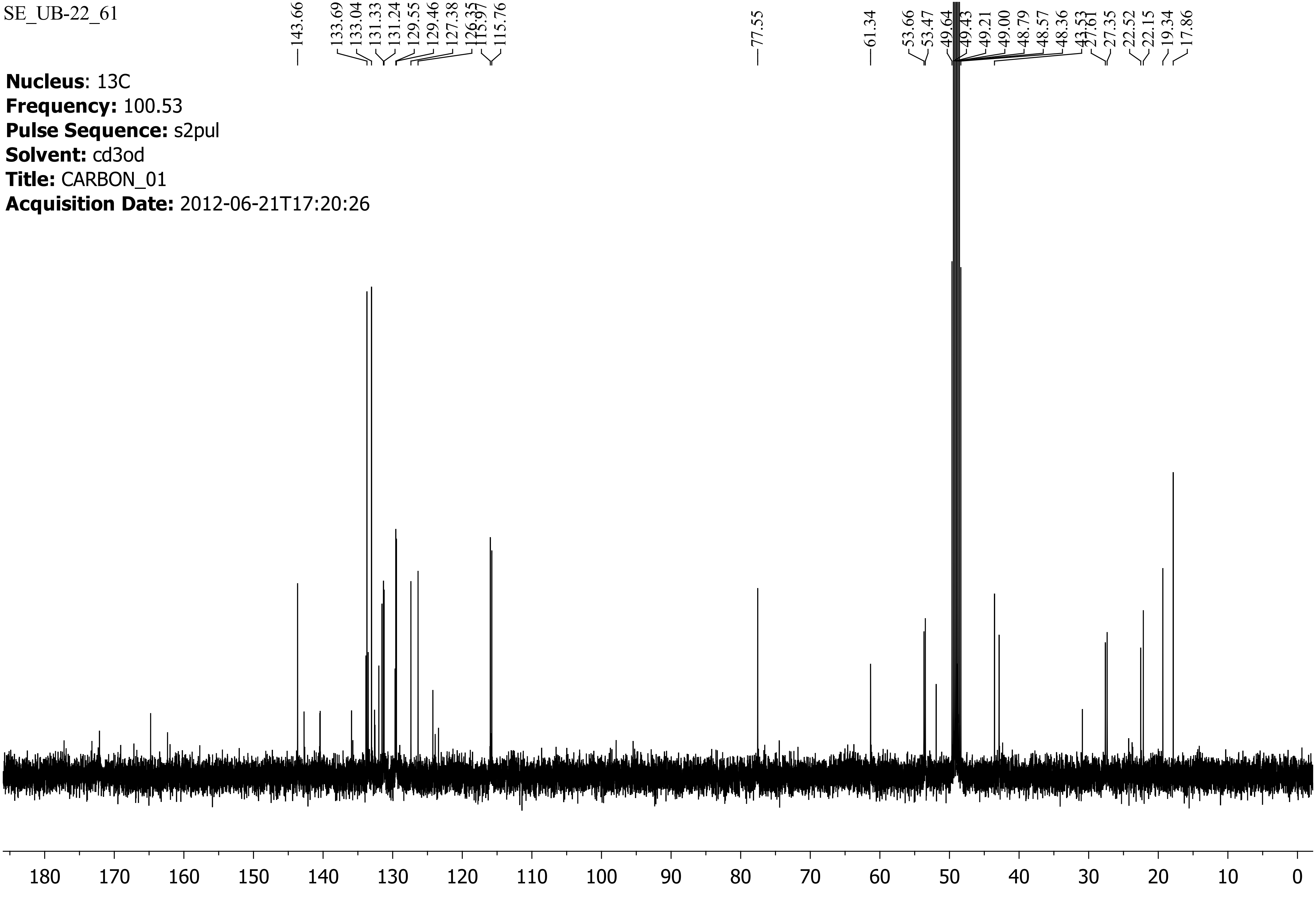

SE_UB-23_42

Nucleus: 1H
Frequency: 399.75
Pulse Sequence: s2pul
Solvent: dmso
Title: PROTON_01
Acquisition Date: 2012-06-22T17:38:30

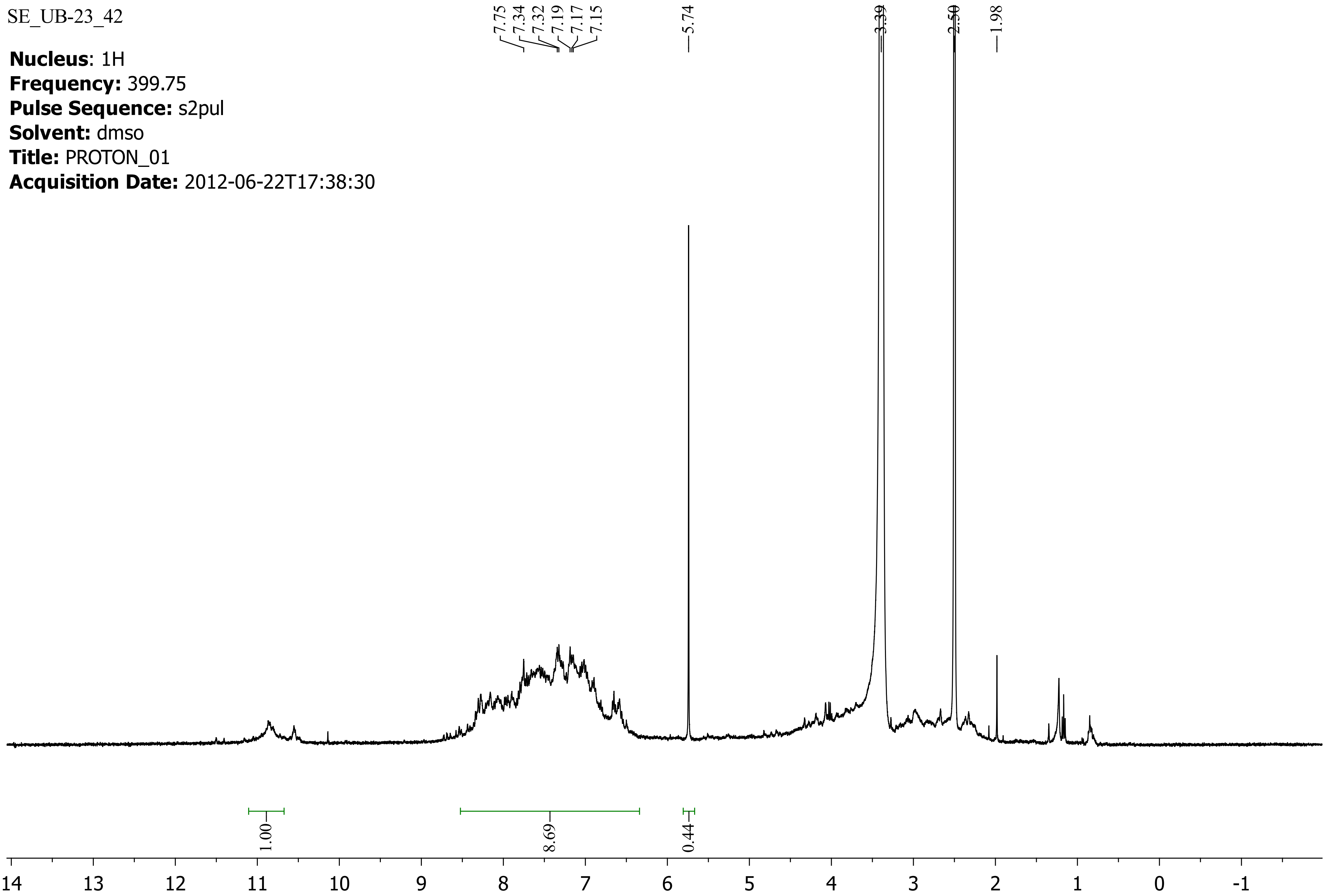

SE_UB-23_43

Nucleus: 1H
Frequency: 399.75
Pulse Sequence: s2pul
Solvent: cdcl3
Title: PROTON_01
Acquisition Date: 2012-06-19T13:33:57

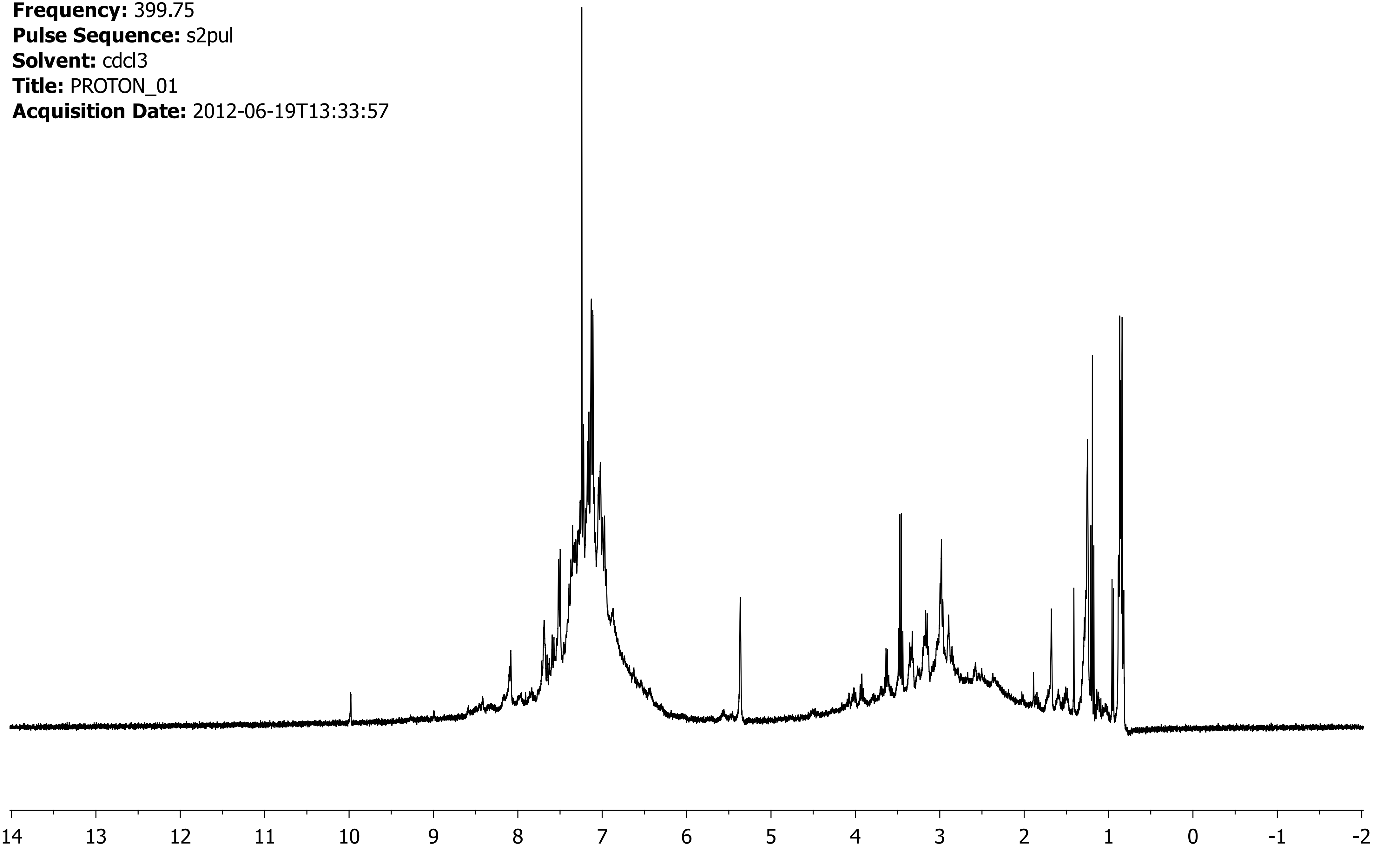

SE_UB-23_44

Nucleus: 1H
Frequency: 399.75
Pulse Sequence: s2pul
Solvent: cdcl3
Title: PROTON_01
Acquisition Date: 2012-06-19T13:48:09

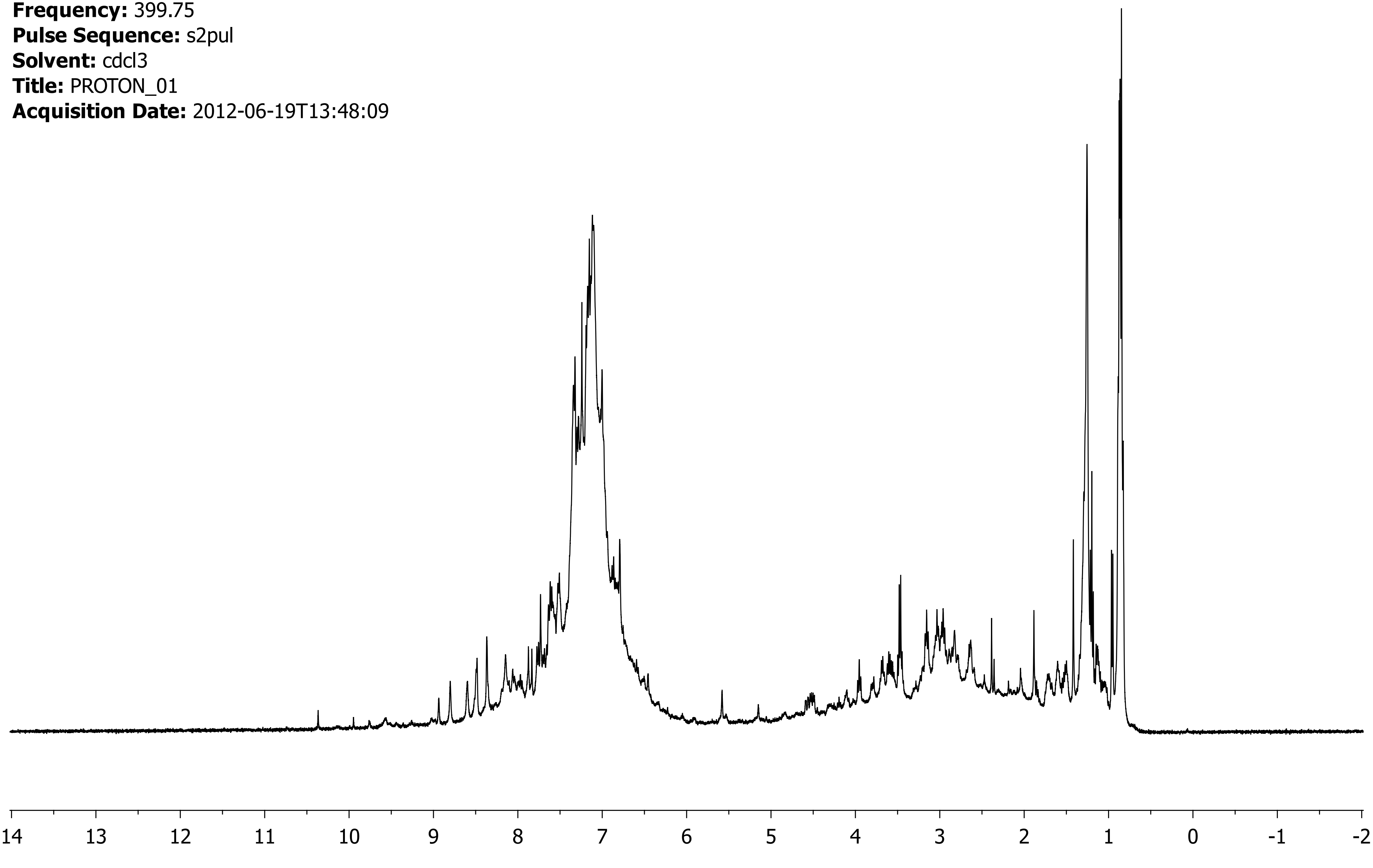

SE_UB-23_45

Nucleus: 1H
Frequency: 399.75
Pulse Sequence: s2pul
Solvent: cdcl3
Title: PROTON_01
Acquisition Date: 2012-06-19T14:02:33

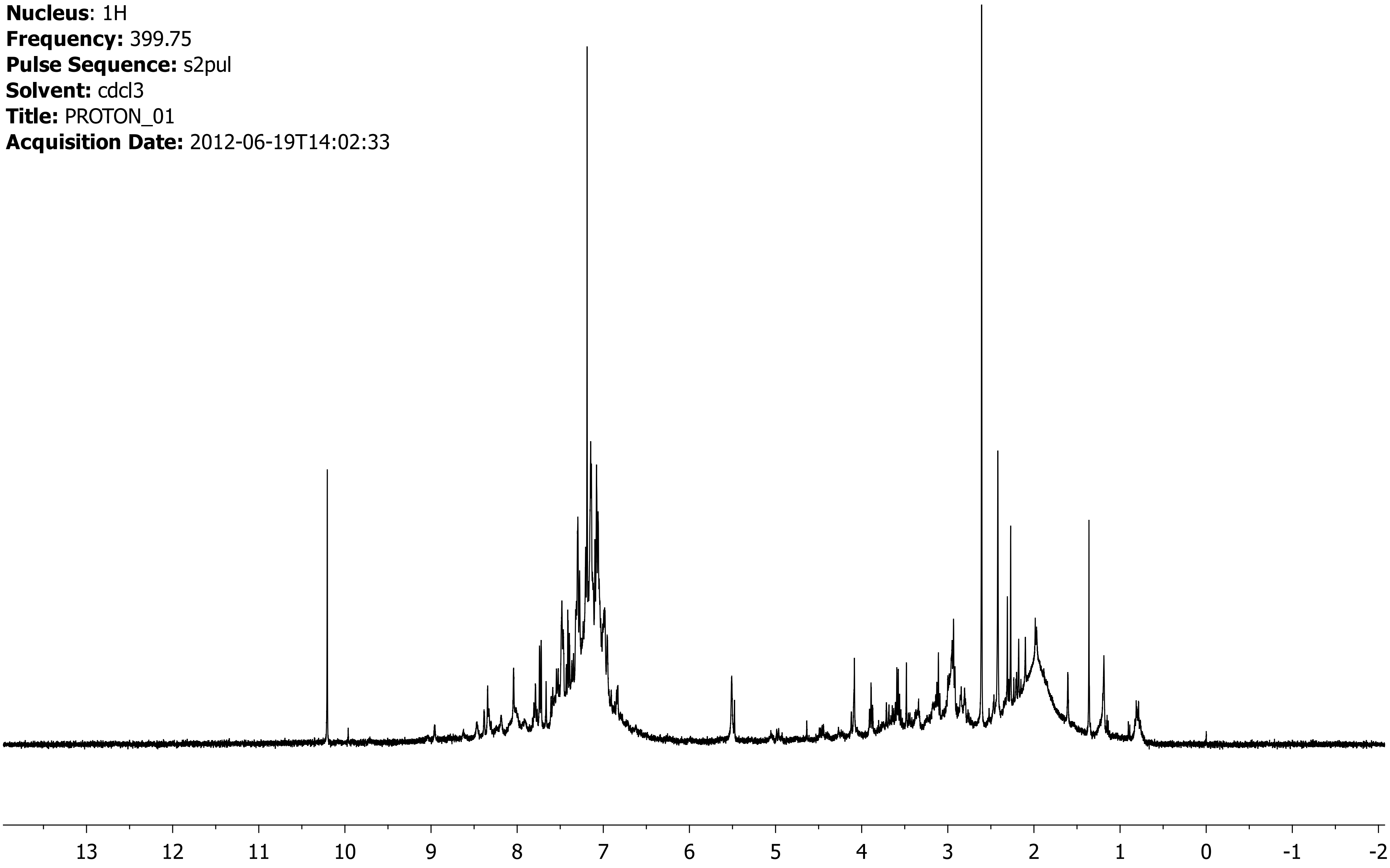

SE_UB-23-46

Nucleus: 1H
Frequency: 399.75
Pulse Sequence: s2pul
Solvent: cdcl3
Title: PROTON_01
Acquisition Date: 2012-06-19T14:06:51

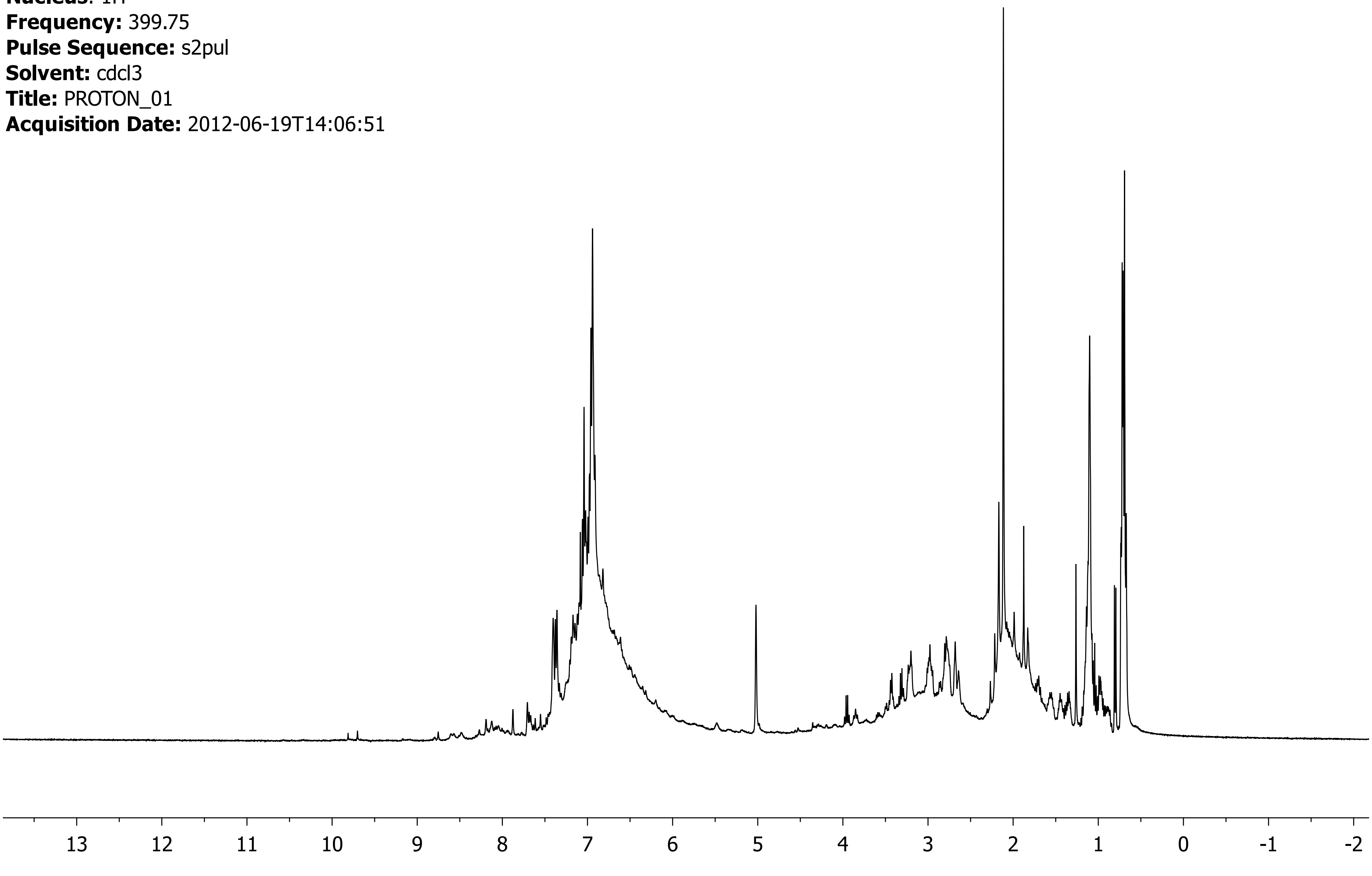

SE_UB-23_46

Nucleus: 1H
Frequency: 399.75
Pulse Sequence: s2pul
Solvent: cd3od
Title: PROTON_01
Acquisition Date: 2012-06-21T18:39:37

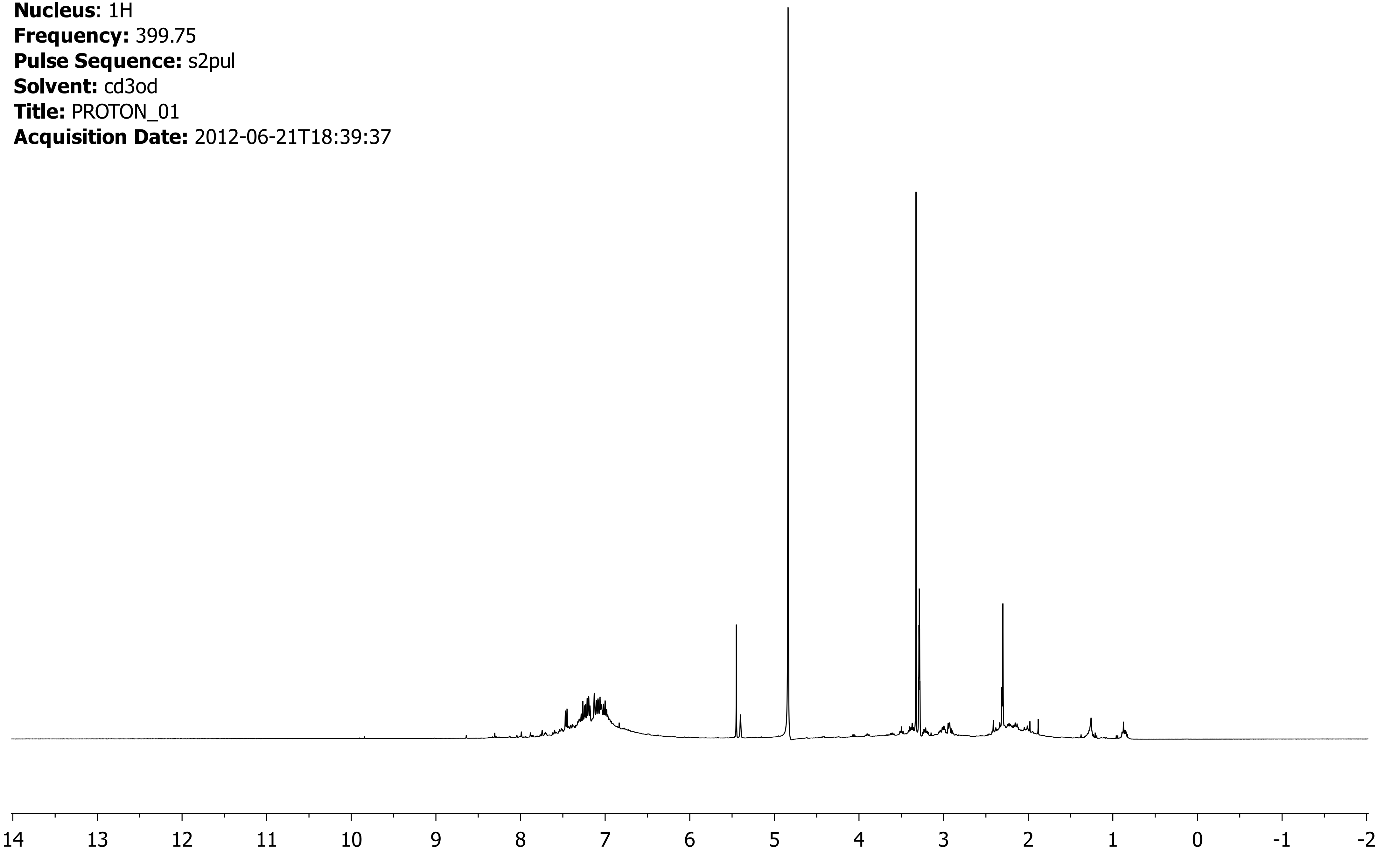

Supplement: Supplementary file 3 — 10.1186/s13065-016-0200-1 NMR data for synthesized compounds (part 2). [file 13065_2016_200_MOESM3_ESM.pdf]
